# Family-focused intervention to promote adolescent mental health and well-being in Moldova and North Macedonia (FLOURISH): feasibility study protocol

**DOI:** 10.1136/bmjopen-2023-080400

**Published:** 2023-12-10

**Authors:** Yulia Shenderovich, Antonio Piolanti, Viorel Babii, Nevena Calovska-Hertzog, Rhiannon E Evans, Nina Heinrichs, Anita Burgund Isakov, Galina Lesco, Graham Moore, Janina Mueller, Marija Raleva, Bojan Shimbov, Judit Simon, Franziska Waller, Dennis Wienand, Heather M Foran

**Affiliations:** 1 Centre for the Development, Evaluation, Complexity and Implementation in Public Health Improvement (DECIPHer), School of Social Sciences, Cardiff University, Cardiff, UK; 2 Wolfson Centre for Young People's Mental Health, Cardiff University, Cardiff, UK; 3 Institute of Psychology, University of Klagenfurt, Klagenfurt, Austria; 4 Asociatia Obsteasca Sanatate Pentru Tineri (Health for Youth Association), Chisinau, Moldova; 5 Department for Psychology, Faculty for Media and Communication, Singidunum University, Belgrade, Serbia; 6 AST Centre for Education, Belgrade, Serbia; 7 Department of Psychology, Bielefeld University, Bielefeld, Germany; 8 Faculty of Political Sciences, Department of Social Policy and Social Work, University of Belgrade, Belgrade, Serbia; 9 Institute for Marriage, Family and Systemic Practice—ALTERNATIVA, Skopje, North Macedonia; 10 Department of Child and Adolescent Psychiatry, Ss Cyril and Methodius University in Skopje, Skopje, North Macedonia; 11 Instituto de Economía Internacional, Department of Economics, University Jaume I Castellon, Castellón de la Plana, Spain; 12 Department of Health Economics, Center for Public Health, Medical University of Vienna, Vienna, Austria; 13 Department of Psychiatry, University of Oxford, Warneford Hospital, Oxford University, Oxford, Oxfordshire, UK

**Keywords:** adolescents, public health, mental health

## Abstract

**Introduction:**

Family-Focused Adolescent & Lifelong Health Promotion (FLOURISH) project will adapt, implement and evaluate a programme to support adolescent mental health and well-being through strategies, such as strengthening parenting practices, adolescent-caregiver relationships, adolescent and parent socioemotional skills, and social support.

**Methods and analysis:**

The project will focus on adolescents aged 10–14 years and their caregivers in North Macedonia and Moldova. The countries were selected based on implementation readiness of two organisations and a need for accessible evidence-informed services to help mitigate health risks due to economic, social and political challenges. Parenting for Lifelong Health (PLH) for Parents and Teens is a family-based programme developed for low-resource settings. PLH has been adapted with input from advisory groups. The programme includes additional components to strengthen impacts on adolescents: adolescent mental health tools, based on UNICEF’s Helping Adolescents Thrive, adolescent peer support and participation booster. This pilot is first of three study phases. The pilot will be a feasibility testing of the adapted intervention and the assessment and implementation procedures to determine further refinements. The pilot will examine if the adapted programme is acceptable for adolescents, their families and providers, explore contextual factors relevant to embedding this programme into longer-term scale-up and investigate whether the programme can be delivered with fidelity and participation; whether the participants report changes in adolescent emotional and behavioural problems, well-being and other outcomes; and whether the study tools are feasible and appropriate. Pre-post adolescent and caregiver questionnaires will provide outcome data. Process evaluation will include attendance and fidelity data, and focus groups. We will examine delivery cost and resource requirements.

**Ethics and dissemination:**

The study was approved at the University of Klagenfurt (Austria), Medical Faculty at St. Cyril and Methodius University (North Macedonia) and National Committee of Ethical Expertise for Clinical Trials (Moldova). Through stakeholder engagement and dissemination, FLOURISH will advance scale-up of open-source family interventions.

**Trial registration number:**

Trial registration: ID101095528; project page: https://www.flourish-study.org/about.html; https://www.linkedin.com/company/flourish-study/

**STRENGTHS AND LIMITATIONS OF THIS STUDY**

- This pilot study is embedded in a project designed to address a gap in evidence-informed programmes to promote adolescent mental health and well-being in North Macedonia and Moldova, at a time when migration, threat of war and economic challenges exert particular pressures on adolescents and their families.
- The study is adapting an open-access intervention to new contexts, with a focus on scalability, to identify a model for adolescent-parent programmes that can be embedded in long-term delivery to provide high-quality and accessible services in both countries.
- The pilot evaluation has a relatively small sample size and therefore cannot provide definitive information on the effects of the programme and its components; however, it will provide initial results on the feasibility of the intervention, its implementation and evaluation; effectiveness will be evaluated in further phases with a randomised design.

## Introduction

Adolescence is a period of transition and transformation when one can acquire and strengthen factors contributing to lifelong health.^1^ Mental health problems during early adolescence are a global concern as this is when about half of mental health problems emerge.^2^ Stressful experiences in early adolescence are more strongly associated with a shorter total life span than such stressors in other phases of childhood, suggesting early adolescence is a sensitive period, making it a crucial time for providing additional support.^3^

Having supportive relationships is associated with good health.^4^ The relationships adolescents have with their parents can remain influential, even as peers become increasingly important. Parents or caregivers are defined broadly to include any caregiver providing significant care to an adolescent, not limited to a biological parent.^5^ Parenting programmes focusing on the adolescent-caregiver relationships and parenting practices are recommended to support adolescent mental health.^6–8^ Furthermore, the WHO Guidelines on mental health promotive and preventive interventions for adolescents also recommend interventions for adolescents’ interpersonal and emotional regulation skills.^9^

A meta-analysis found no significant difference in effectiveness between transported and homegrown parenting interventions for reducing disruptive child behaviour.^10^ However, culturally adapted parenting interventions, both homegrown and transported, were more effective than non-adapted interventions.^11^ Adapting interventions can help maintain effectiveness while avoiding the cost of developing new interventions.^12^ Although the majority of adolescents live in low- and middle-income countries (LMICs), research with adolescents in most LMICs is very limited,^13^ so evidence often has to be adapted from elsewhere.

The Family-Focused Adolescent & Lifelong Health Promotion (FLOURISH) project (2023–2026) is focused on adapting, optimising and evaluating an intervention package for adolescents aged 10–14 years and their caregivers in the Republic of North Macedonia and the Republic of Moldova (henceforth North Macedonia and Moldova). The intervention package aims to support adolescent mental health and well-being through building adolescents’ and caregivers’ skills, such as problem-solving and emotional regulation, strengthening social support, adolescent-caregiver relationships and communication, and improving parenting practices.

Moldova and North Macedonia were selected based on high risks for adolescent mental health, including adverse childhood experiences and poverty, and lack of evidence-informed and accessible provision of prevention programmes for families with adolescents, paired with high levels of implementation readiness in two health networks. North Macedonia and Moldova are middle-income countries, among the poorest in Europe.^14^ Both have experienced rapid social, political and economic transformation since 1990s following the fall of Communism.

Studies found high rates of physical punishment, emotional violence and neglect against children in both countries.^15–17^ Work migration often leads caregivers to spend time away from children, which can hamper communication.^5^ Young people also face limited work opportunities. Existing challenges were magnified by the COVID-19 pandemic^18–20^ and the war in Ukraine. There is stigma around help seeking, particularly regarding mental health and well-being.^21^ A recent report found that in this region, support services for caregivers of adolescents, such as parenting workshops or support groups, are scarce and highlighted the need for more such services,^5^ which our project aims to help address.

This protocol describes the adapted intervention, designed to promote adolescent mental health and well-being in Moldova and North Macedonia, and introduces the FLOURISH feasibility pilot (2023–2024), which will inform the next phases of a larger study by providing information for further refinement of the intervention, its implementation and evaluation procedures.

## Methods

### Research questions and frameworks

FLOURISH is shaped by the Multiphase Optimisation Strategy (MOST) framework. MOST is designed to optimise an intervention package within the key constraints in three phases—preparation, optimisation and evaluation.^22 23^ This pilot study corresponds to the preparation phase, focused on preparing and piloting the intervention and its evaluation. Following the pilot, a factorial trial will be conducted to select the most effective and efficient treatment package (optimisation phase). In the evaluation phase, the revised programme package will be tested in an implementation-effectiveness randomised controlled trial.

Next, we discuss the research questions and additional frameworks and guidelines used in answering them (see table 1 for an overview).

**Table 1:** Overview of the study design

Data	Participants	Research question	Framework
Advisory group consultations, focus groups	Adolescents, caregivers, staff and (only for advisory groups) external professionals	1	ADAPT, process evaluation guidelines
Interviews	External professionals	2	CICI, ExpandNet
Attendance registers, observations, focus groups	Staff	3	Process evaluation guidelines
Questionnaires	Adolescents and caregivers	4	MOST
Think-aloud interviews, questionnaires	Adolescents and caregivers	5	N/A

CICI, Context and Implementation of Complex Interventions; MOST, Multiphase Optimisation Strategy; N/A, not applicable.

1. Is the adapted programme acceptable for adolescents, their caregivers and staff in North Macedonia and Moldova, and what further adaptations are needed?

Programme materials have been adapted prior to piloting and will be revised throughout the project. FLOURISH draws on ADAPT adaptation guidance, which emphasises improving intervention fit with a new context while preserving key intervention functions.^12 24^

2. What contextual factors may influence embedding of the programme into sustainable delivery and funding mechanisms in North Macedonia and Moldova?

FLOURISH is guided by the principle of ‘beginning with the end in mind’ in ExpandNET/WHO guidance.^25^ We will adapt the programme alongside developing a scaling-up strategy. This includes building capacity for scale-up and making choices to support institutionalisation and expansion to more settings.

3. Can the adapted programme be delivered with high fidelity and family participation?

The pilot includes a process evaluation, guided by UK Medical Research Council process evaluation guidance,^26^ which focuses on implementation processes, potential mechanisms of impact and contextual factors. To organise our understanding of the country context, we are using the Context and Implementation of Complex Interventions (CICI) framework.^27^

4. What are the changes, if any, reported by the adolescents and caregivers on the primary outcomes of adolescent emotional problems, behavioural problems and well-being and on the secondary outcomes for adolescents and caregivers?

Primary programme targets are adolescent mental health and well-being. The pilot study will assess pre-post changes in the intervention outcomes reported by adolescents and their caregivers, following lessons learnt in a previous pilot feasibility study, on which this project builds.^28 29^

5. Are the study tools and their translations feasible and appropriate?

The pilot will examine the feasibility and preliminary psychometric performance of the outcome measures and their translations. A rigorous translation and evaluation process will be followed. The pilot will provide insights on the feasibility of the procedures and tools and inform the next phase of the project, including which tools are retained, removed or modified.

### Study sites

The study is conducted in two country-wide health networks, Institute for Marriage, Family and Systemic Practice (ALTERNATIVA) and Health for Youth Association. ALTERNATIVA is a network of psychologists, social workers and family therapists in North Macedonia. Health for Youth Association in Moldova is an organisation that supports the activity of publicly funded youth-friendly clinics offering prevention and treatment in youth sexual and reproductive health, mental health, substance use and violence prevention. Both organisations work with caregivers and adolescents and have delivered Parenting for Lifelong Health (PLH) programmes and participated in evaluations.^28^

### Study design

#### Intervention programme

The core programme examined in FLOURISH is PLH for Parents and Teens. It is a group behavioural programme,^30 31^ based on social learning theory.^32^ PLH was developed for LMICs in collaboration with UNICEF, WHO and other international organisations and universities. Programme materials are focused on strengthening psychosocial skills and relationships and are freely available online: https://www.who.int/‍teams/‍social-‍determinants-of-health/‍parenting-for-lifelong-health.

PLH programmes, primarily for caregivers with young children (aged 2–9 years), have been evaluated in multiple studies, including completed and ongoing trials in South Africa, the Philippines, El Salvador, Lesotho, North Macedonia, Moldova, Romania, Thailand, Uganda and Zambia. Evaluations reported high levels of engagement by families, practitioners and other stakeholders. A pilot feasibility study and two large randomised trials in North Macedonia, Moldova and Romania found improvements in parenting behaviours and child mental health,^29 33^ and further results are forthcoming (Foran *et al*; Heinrichs *et al*). PLH for Parents and Teens (aged 10–17 years) has been evaluated in South Africa in two pre-post studies and a randomised controlled trial (552 families, 40 clusters), with an embedded process evaluation.^34 35^ At 5–9 months after the 14-week intervention, caregivers in the intervention group, but not adolescents, reported lower rates of violence towards their adolescents and corporal punishment, and both caregivers and adolescents reported greater involved parenting and supervision of the adolescent by the caregiver (primary outcomes). There was no intervention effect detected on adolescent depression, suicidality and externalising behaviour—however, these secondary outcomes were not the focus of the intervention.

#### Adaptation of the intervention

**Figure 1:**
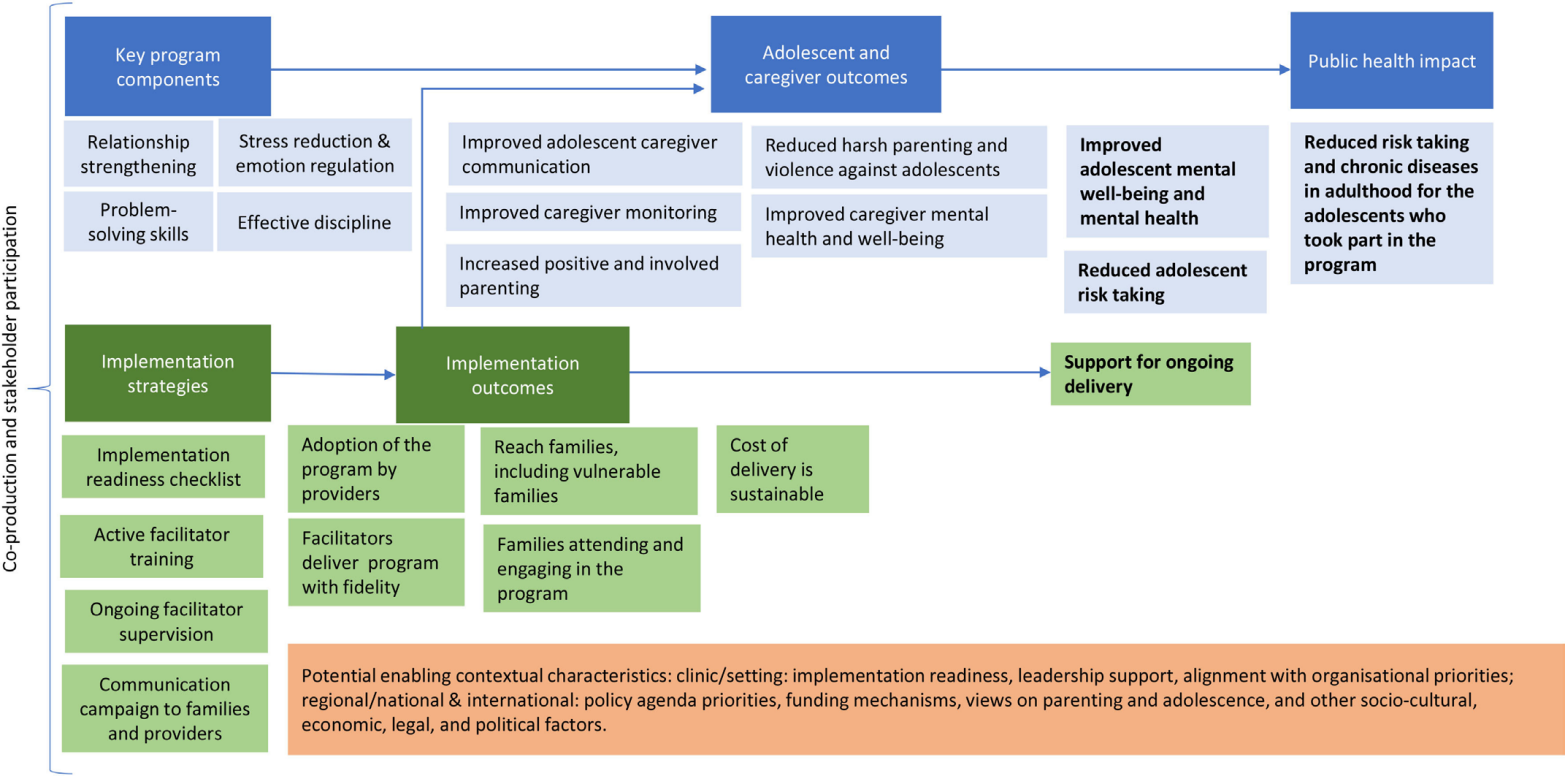
Initial programme theory.

The adapted programme is drawing on the PLH manual used in the South African evaluation and on other programme versions. The initial adaptations have been informed by the input of advisory groups and expert interviews (described below), team’s clinical expertise and input from the programme developers. The adapted programme will be tested in the pilot (see the initial programme theory in figure 1, to be revised throughout the project).

To support scalability, the programme was condensed into six group sessions, following on an introductory meeting with the caregiver and adolescent. As in the original model, sessions in FLOURISH will be delivered to a group of 10 adolescent-caregiver pairs. Each 2-hour weekly session starts jointly, with a circle share, home activity discussion, agenda overview, physical exercises and games. Next, in the core lesson, a new skill is introduced and practised, on topics developmentally relevant to adolescence. For the core lesson, the group is often split up into adolescent and caregiver parallel sessions to allow participants to share openly (see table 2). After discussing the core lesson within the respective peer groups, participants come together and are encouraged to share the summary of their separate discussions in the joint group and then further discuss lessons learnt at home. Participants are given home practice tasks to do between the sessions. For participants who miss a session, facilitators offer a brief catch-up call or meeting. Facilitators and supervisors receive brief training in the programme and facilitation skills. Supervisors are trained in supervision. The programme is delivered by two facilitators per group, coached weekly by supervisors to promote quality of delivery. Given the focus on scalability, staff are not required to have a specific degree.

**Table 2:** Adapted intervention structure using Parenting for Lifelong Health material

Week	Topic	Caregiver and adolescents
Preprogramme	Individual meeting or call with facilitators to introduce the programme	Joint
1	Introducing the programme and ground rules. Psychoeducation about transitioning from a child to an adolescent: expectations, norms and developmental stages	Separate
2	Relationship skills. Building a positive relationship through spending time together and praising each other	Joint
3	Talking about emotions and sensitive topics (sexuality, body changes and intimate relationships)	Separate
4	Coping with difficult feelings (anger and stress)	Separate
5	Solving problems together as a family. How to communicate and solve problems around disagreements without conflict	Separate
6	Establishing rules and routines	Separate

To strengthen the impacts on adolescent outcomes, the adaptation of the programme involves supplementary components. Three components have been combined with the PLH programme into an intervention package: adolescent mental health tools, adolescent peer support and adolescent participation booster.

#### Adolescent mental health tools

To strengthen adolescent mental health, we draw on the Helping Adolescents Thrive toolkit (HAT), designed to promote positive mental health, prevent mental health problems and reduce engagement in self-harm and risk behaviours.^36^ This toolkit was developed based on a systematic review of universally delivered psychosocial interventions for adolescents,^13^ conducted to inform WHO Guidelines.^9^ The HAT materials will be delivered as six comic chapters, a workbook for adolescents and leaflets for caregivers, including, respectively, tips for adolescents to share their thoughts and feelings with their caregivers and questions caregivers can ask adolescents. The aim of these materials is to support adolescents and caregivers to communicate about adolescent mental health and well-being.

#### Adolescent peer support

Even brief interventions can foster friendships and social connections.^37 38^ Since parenting programmes have traditionally focused on parents, they have demonstrated positive impacts on social support experienced by parents but not by adolescents, including in the previous trial of PLH for Parents and Teens.^30^ The facilitators will pair up adolescents in the group for facilitating peer support and social connection. The PLH programme already includes peer pairings, but its potential effects have not yet been evaluated, so more emphasis will be placed on pairing up adolescents, and this will be tested as a separate component in the optimisation phase.

#### Adolescent participation booster

Although parenting programmes have been found acceptable in diverse settings, there is incomplete attendance.^39–41^ While many studies have explored parental attendance for parents of younger children,^29^ there is limited research on enhancing engagement of adolescents and their caregivers as programmes rarely included adolescents. The South African trial found 50%–64% session attendance.^42^ We will investigate whether an adolescent participation booster promotes attendance.

### Patient and public involvement

Members of the public were involved in the design of this research. In particular, intervention adaptations have been informed by advisory group consultations conducted in May–July 2023, as described below.

#### Advisory groups

Advisory groups of adolescents, caregivers, implementation organisation staff and other professional experts (six to eight people per group) were formed in North Macedonia and Moldova to advise on programme adaptations and the study findings. Consultations will continue throughout the study, with key discussion points captured by a note taker. Adolescents are eligible if they are aged 10–14 years when joining the advisory group and provide adolescent assent and caregiver consent. For caregivers, staff and other professionals, members need to be at the age of 18 years or older and provide consent. For the caregiver advisory group, participants need to be caregivers of adolescents aged 10–17 years (extended age range to facilitate recruitment). The groups aim to include individuals with experience of PLH or other family programmes and individuals who represent diverse perspectives (eg, male and female caregivers and ethnic minorities). Implementation staff groups consist of ALTERNATIVA and Health for Youth Association staff. External professional experts include representatives from ministries of health, social services, youth, universities, research organisations, healthcare financing agencies, local non-government organisations (NGOs), community and international organisations implementing programmes for young people.

#### Advisory group analysis

Summary notes were combined into a matrix, using thematic framework and content analysis.^43 44^ We examined similarities and differences within and between stakeholder groups and countries. Advisory group participants received a summary for feedback as a means of validation.

#### Expert interviews

In addition, in each country, 10 semi-structured audio-recorded interviews with professional experts took place to map the contextual facilitators and barriers to scaling up a programme package prepared by the FLOURISH project. We drew on recommendations for qualitative sample sizes.^45 46^

#### Interview analysis

We are using thematic and framework analysis for the transcripts. The CICI framework provides initial coding domains, with country-specific domains generated from the data. The analysis is conducted in two stages—first, a rapid synthesis to inform the intervention adaptations for the pilot (completed) and, second, a detailed analysis, generating a conceptual map of key contextual factors, presented as a narrative summary (ongoing).

### Piloting of the intervention

The pilot (October 2023–January 2024) will test the adapted intervention in a pre-post uncontrolled study with an outcome and process evaluation, described below.

#### Participants and recruitment

The pilot will include three intervention groups per country, so the target sample size is 60 adolescents and 60 caregivers (60 dyads). Recruitment will involve disseminating information through online and print materials, word of mouth and via the existing clients of each network. At least one caregiver and adolescent per family will participate in the study. Inclusion criteria for caregivers are age 18 years or older at baseline assessment; primary caregiver responsible for the care of an adolescent aged 10–14 years, resident in the same household at least four nights a week in the previous month; able to speak one of the local languages, in which the programme will be offered; agreement to participate in the programme; and provision of consent for self and adolescent to participate in the study. Inclusion criteria for adolescents: age 10–14 years at baseline assessment, assent to participate and caregiver consent.

The caregiver can be any adult who is primarily responsible for looking after the adolescent and available to participate. In cases where there is more than one caregiver who is available, they will need to agree on who will be the primary and secondary participant. Similarly, if two adolescents in the same age range in a family would like to join, both can join. Everyone who joins the study, both primary and secondary participants, will be invited to complete assessments.

Taking a proportionate universalism approach,^47^ all families will be eligible, but we will make greater efforts to include more vulnerable groups. We conceptualise our approach as selected prevention, targeted to individuals whose risk is above average, without screening.^48^ FLOURISH has no exclusion criteria. While introducing the project, the researchers will guide the participants through a consent form that asks whether they are currently experiencing acute distress or a mental or physical health condition that would interfere with participation. The judgement will be made by the potential participants. If they decide they are not able to take part, the research team will follow up and provide referrals for other services (see also Ethics and dissemination section).

To deliver the programme, facilitators and supervisors will be recruited from existing staff and networks of the implementing organisations. Inclusion criteria for facilitators are age 18 years or older, participation in training, agreement to deliver the programme and provision of study consent and for supervisors, age 18 years or older, participation in training, agreement to provide supervision and provision of consent.

#### Pre-post outcomes

Outcome data collection with families will be done primarily using computer-assisted self-interviewing questionnaires on tablets. Trained assessors will check participant eligibility as part of the informed consent procedure and assist adolescents and caregivers with completing the questionnaires. See table 3 for the primary outcome measures and online supplemental table 1S for secondary outcome measures and online supplemental table 2S for other measures (preregistered in clinical trials registry: ID101095528).

10.1136/bmjopen-2023-080400.supp1 Supplementary data

**Table 3:** Primary outcomes

Construct	Respondent	Measure
Adolescent emotional problems	Adolescent	The Revised Child Anxiety and Depression Scales, anxiety and depression subscales^57–59^ Child Behaviour Checklist 6–18, internalising subscale^60^ Youth Self-Report 11–18, internalising subscale^60^
Adolescent behavioural problems	Adolescent	Youth Self-Report, rule-breaking and aggressive behaviour
	Caregivers	Child Behaviour Checklist, rule-breaking and aggressive behaviour
Adolescent well-being	Adolescent	WHO-Five Well-Being Index (WHO-5)^61 62^

Outcome measures without available official translations will be adapted and translated following best practices.^49^ First, a cultural evaluation (relevance and appropriateness) of each item and forward translation will be conducted by a professional with mental health experience. Second, a back translation to English will be carried out by another translator. Third, an additional translator will compare the translations and assess correspondence between semantic equivalence and words, highlighting any discrepancies. A final translation will be produced in a meeting between the translators. All translators will be bilingual.

Think-aloud interviews will be conducted for the primary outcome measures that have not been previously used with adolescents or in the study countries (Revised Child Anxiety and Depression Scales and WHO-5) and for the health economic measures that will be used in the optimisation and evaluation phases—European Quality of Life 5 Dimensions 5 Level Version,^50^ European Quality of Life 5 Dimensions Youth Version,^51^, Oxford CAPabilities questionnaire-Mental Health (OxCAP-MH) 52 53 and PECUNIA Resource Use Measurement (RUM) Instrument^54^. Think-aloud interviews will be conducted after post-test assessments and explore item comprehension (10 interviews per country with adolescents and caregivers in Romanian and Macedonian).

#### Pre-post outcomes analysis

We will examine descriptive and preliminary psychometric statistics, such as internal consistencies. Intent-to-treat analyses will examine pre-post changes in adolescent and caregiver outcomes. We hypothesise that adolescent emotional problems and adolescent behavioural problems, both in self-report and caregiver report, will be significantly reduced at the post-assessment; adolescent well-being will be significantly increased at the post-assessment, with medium to large effect sizes (Cohen’s d=>0.5). We also will examine pre-post change in secondary outcomes and hypothesise small to medium effect sizes. Missing data will be addressed using full information maximum likelihood or multiple imputation methods.

#### Process evaluation

Facilitators will collect weekly attendance registers. Programme supervisors will conduct fidelity assessments. One session per facilitator will be observed live or video-recorded and assessed using the recording with the Facilitator Assessment Tool.^55^ Facilitators and supervisors will complete questionnaires to provide information on demographics and previous experience and to collect pre-post data on staff well-being and parenting stress (for staff who are caregivers). The sample sizes are determined by the delivery model and the number of families in the intervention study.

#### Process evaluation analysis

We will use descriptive statistics, summarising enrolment, attendance, fidelity and facilitator characteristics. We will check variation in attendance by participant baseline characteristics (eg, gender) and by intervention delivery characteristics (eg, study site) to surface any emerging inequalities. We will explore whether attendance varied across sessions. We will explore staff responses to the pre-post well-being questionnaires using descriptives and paired t-tests. This will help inform, for example, whether additional support is needed if staff well-being had reduced.

#### Intervention costs

Facilitators and supervisors will complete questionnaires to capture the time and other resources spent preparing for, delivering and following up on intervention activities. They will complete one form for the initial training and weekly forms designed to separate the resources required for the intervention versus the study.

#### Costs analysis

We will assess if weekly surveys are feasible. We will conduct descriptive analyses, summarising information on time, money and other resources used. Resource use information will be costed with country-specific unit costs collected primarily for the study to provide overall cost data. Cost information will be used to inform scale-up planning and, in later phases, for programme cost-effectiveness analyses.

#### Focus groups

To inform further programme adaptations and answer process evaluation questions about the context, how the programme was implemented and its mechanisms of change, we will conduct participant focus groups. A subsample of the adolescents, caregivers and intervention staff will be recruited to participate in post-programme audio-recorded focus groups (six to eight people/group, minimum of three groups/country). We will aim to select participants with diverse backgrounds and experiences.

#### Focus group analysis

Focus group transcripts will be analysed using thematic and framework analysis, with codes developed both based on the initial questions and unexpected insights.

## Ethics and dissemination

An ethical self-assessment for Horizon Europe was reviewed by the European Commission, and the project was cleared for ethics. The study was approved by the Institutional Review Board for Research Ethics at the University of Klagenfurt, Austria (coordinating site); the Ethical Commission for Human Research at the Medical Faculty at St. Cyril and Methodius University, North Macedonia; and the National Committee of Ethical Expertise for Clinical Trials of the Ministry of Health, Moldova.

Participants will provide written assent (adolescents) or consent (adults). Adolescents and caregivers will receive reimbursements for transport costs and thank-you vouchers for data collection activities, worth ~€10. All staff will be trained to identify and handle potential distress in participants with detailed safety procedures and referral processes. Adverse event monitoring will help detect any potential harm caused by the research or the intervention. Any serious adverse events will be investigated and reported to the ethics committees and the data and safety monitoring board to consider potential changes in the project.

The FLOURISH project emphasises dissemination to stakeholders. Dissemination will include meetings, communication with media, and through print and online channels, including existing networks, FLOURISH website and social media. The focus of dissemination is Moldova and North Macedonia, and we will also be engaging stakeholders in the Eastern Europe region, working with family and adolescent health associations, NGOs, government and international agencies.

This pilot will provide insights on the feasibility of the intervention, its implementation and evaluation, and thus inform the factorial trial planned as the next research phase, guiding which changes need to be made to the intervention, implementation and evaluation procedures. More broadly, this project will provide insights on how best to adapt and scale-up a programme targeting family relationships and adolescent mental health at a time when economic problems, interpersonal and intergroup conflict, the threat of a wider war and other issues contribute to increased stress for both adolescents and adults. We will draw on a set of frameworks and global health best practices,^56^ such as using open-access interventions that involve stakeholders in all stages of intervention adaptation and evaluation.

## Supplementary Material

Reviewer comments

Author's manuscript
