# Supplementary material for: Family-focused intervention to promote adolescent mental health and well-being in Moldova and North Macedonia (FLOURISH): feasibility study protocol
Source: BMJ Open. 2023 Dec 10;13(12):e080400. doi: 10.1136/bmjopen-2023-080400 (PMC11148709; doi:10.1136/bmjopen-2023-080400)
Supplement: Supplementary data [file bmjopen-2023-080400supp001.pdf]

Supplementary materials

Table 1s Secondary outcome measures

Construct	Respondent	Measure
Communication	Adolescents & parents	Child-Parent Communication Apprehension scale, total score (1)
		Family Assessment Device-FAD, general functioning subscale (2)
Involved parenting	Adolescents & parents	Alabama Parenting Questionnaire (3), involved parenting and parental supervision/monitoring subscales.
Harsh parenting	Parents	Alabama Parenting Questionnaire (3), corporal punishment subscale.
Adolescent emotional problems	Adolescents	The Revised Child Anxiety and Depression Scales (RCADS), total score (4–6)
Adolescent behavior problems	Parents	Child Behavior Checklist 6-18, externalizing behavior (7)
Loneliness	Adolescents	UCLA-8 Loneliness scale, total score (8)
	Parents	Revised UCLA-6 Loneliness scale, total score (9)
Social support	Adolescents & parents	Medical Outcome Study Social Support Survey, emotional and affectionate sub-scales (10)
Parents wellbeing	Parents	WHO-5 Well-Being Index, total score (WHO-5) (11,12)
Parents psychological distress	Parents	The Patient Health Questionnaire – 9 (PHQ-9) , total score (13).
Parental stress	Parents	Parental Stress Scale, total score (14).

Shenderovich Y, *et al. BMJ Open* 2023; 13:e080400. doi: 10.1136/bmjopen-2023-080400

Table 2s. Other measures

Construct	Respondent	Measure
Enrolment	Facilitators	Percentage of participants who joined the study and have attended at least one group PLH session (individually for adolescents and caregivers, for both jointly at the dyad/family level)
Attendance	Facilitators	Percentage of group PLH sessions attended out of 6 (individually for adolescents and caregivers, for both jointly at the dyad/family level)<br>Percentage of catch-up PLH contact out of 6 (individually for adolescents and caregivers)
Adolescent healthy weight	Adolescents & parents	Body mass index (not expected to change but assessed for sample description).
Alcohol use	Parents	The Alcohol Use Disorder Identification Test (15).
Posttraumatic stress	Parents	PTSD checklist for DSM-5 (PCL-5), short form, total score
	Adolescent	Children's Revised Impact of Event Scale (16), total score and subscales
Sexual risk behaviors	Adolescents	Health Behavior in School-aged Children Study Sexual health scale, total score (17).
Adolescent-defined problems	Adolescent	The Top Problem Assessment (18–20).
Demographics	Adolescent & parents	Parent and adolescent age, gender, education, economic status.

REFERENCES

1. Lucchetti AE, Powers WG, Love DE. The Empirical Development of the Child-Parent Communication Apprehension Scale for Use With Young Adults. Journal of Family Communication [Internet]. 2002 Jul;2(3):109–31. Available from: http://www.tandfonline.com/doi/abs/10.1207/S15327698JFC0203_1

2. Epstein NB, Baldwin LM, Bishop DS. The McMaster family assessment device. Journal of Marital and Family Therapy [Internet]. 1983 Apr;9(2):171–80. Available from: https://onlinelibrary.wiley.com/doi/10.1111/j.1752-0606.1983.tb01497.x

3. Frick PJ. Alabama Parenting Questionnaire. Unpublished rating scale, University of Alabama. 1991;

4. Chorpita BF, Moffitt CE, Gray J. Psychometric properties of the Revised Child Anxiety and Depression Scale in a clinical sample. Behaviour Research and Therapy [Internet]. 2005 Mar;43(3):309–22. Available from: https://linkinghub.elsevier.com/retrieve/pii/S0005796704000695

5. Krause K, Midgley N, Edbrooke-Childs J, Wolpert M. A comprehensive mapping of outcomes following psychotherapy for adolescent depression: The perspectives of young people, their parents and therapists. European Child and Adolescent Psychiatry [Internet]. 2021;30(11):1779–91. Available from: https://doi.org/10.1007/s00787-020-01648-8

6. Krause KR, Chung S, Adewuya AO, Albano AM, Babins-Wagner R, Birkinshaw L, et al. International consensus on a standard set of outcome measures for child and youth anxiety, depression, obsessive-compulsive disorder, and post-traumatic stress disorder. The Lancet Psychiatry [Internet]. 2021 Jan;8(1):76–86. Available from: https://linkinghub.elsevier.com/retrieve/pii/S2215036620303564

7. Achenbach TM, Rescorla L. The Manual for the ASEBA School-Age Forms & Profiles. Burlington; 2001.

8. Roberts RE, Lewinsohn PM, Seeley JR. A Brief Measure of Loneliness Suitable for Use with Adolescents. Psychological Reports [Internet]. 1993 Jun;72(3_suppl):1379–91. Available from: http://journals.sagepub.com/doi/10.2466/pr0.1993.72.3c.1379

9. Wongpakaran N, Wongpakaran T, Pinyopornpanish M, Simcharoen S, Suradom C, Varnado P, et al. Development and validation of a 6-item Revised UCLA Loneliness Scale (RULS-6) using Rasch analysis. British Journal of Health Psychology [Internet]. 2020 May 30;25(2):233–56. Available from: https://onlinelibrary.wiley.com/doi/10.1111/bjhp.12404

10. Sherbourne CD, Stewart AL. The MOS social support survey. Social Science and Medicine. 1991;32(6):705–14.

11. Topp CW, Østergaard SD, Søndergaard S, Bech P. The WHO-5 Well-Being Index: A Systematic Review of the Literature. Psychotherapy and Psychosomatics [Internet]. 2015;84(3):167–76. Available from: https://www.karger.com/Article/FullText/376585

12. WHO. Wellbeing Measures in Primary Health Care/The Depcare Project. Copenhagen; 1998.

13. Kroenke K, Spitzer RL, Williams JBW. The PHQ-9: validity of a brief depression

severity measure. Journal of General Internal Medicine [Internet]. 2001 Sep;16(9):606–13. Available from: http://link.springer.com/10.1046/j.1525-1497.2001.016009606.x

14. Berry JO, Jones WH. The parental stress scale: Initial psychometric evidence. Journal of Social and Personal Relationships. 1995;12(3):463–72.

15. Saunders JB, Aasland OG, Babor TF, De la Fuente JR, Grant M. Development of the Alcohol Use Disorders Identification Test (AUDIT): WHO Collaborative Project on Early Detection of Persons with Harmful Alcohol Consumption-II. Addiction. 1993;88(6):791–804.

16. Perrin S, Meiser-Stedman R, Smith P. The Children’s Revised Impact of Event Scale (CRIES): Validity as a Screening Instrument for PTSD. Behavioural and Cognitive Psychotherapy [Internet]. 2005 Oct 14;33(4):487–98. Available from: https://www.cambridge.org/core/product/identifier/S1352465805002419/type/journal_article

17. Young H, Költo A, Reis M, Saewyc EM, Moreau N, Burke L, et al. Sexual Health questions included in the Health Behaviour in School-aged Children (HBSC) Study: An international methodological pilot investigation. BMC Medical Research Methodology. 2016;16(1):1–12.

18. Milgram L, Tonarely NA, Ehrenreich-May J. Youth top problems and early treatment response to the unified protocols for transdiagnostic treatment of emotional disorders in children and adolescents. Child Psychiatry & Human Development [Internet]. 2021 Mar 17; Available from: http://link.springer.com/10.1007/s10578-021-01151-4

19. Weisz JR, Chorpita BF, Frye A, Ng MY, Lau N, Bearman SK, et al. Youth top problems: Using idiographic, consumer-guided assessment to identify treatment needs and to track change during psychotherapy. Journal of Consulting and Clinical Psychology. 2011;79(3):369–80.

20. Weisz JR. Testing standard and modular designs for psychotherapy treating depression, anxiety, and conduct problems in youth. Archives of General Psychiatry [Internet]. 2012 Mar 1;69(3):274. Available from: http://archpsyc.jamanetwork.com/article.aspx?doi=10.1001/archgenpsychiatry.2011.147
