# Supplementary material for: Family-focused intervention to promote adolescent mental health and well-being in Moldova and North Macedonia (FLOURISH): feasibility study protocol
Source: BMJ Open. 2023 Dec 10;13(12):e080400. doi: 10.1136/bmjopen-2023-080400 (PMC11148709; doi:10.1136/bmjopen-2023-080400)
Supplement: Author's manuscript [file bmjopen-2023-080400.draft_revisions.pdf]

BMJ Open

BMJ Open is committed to open peer review. As part of this commitment we make the peer review history of every article we publish publicly available.

When an article is published we post the peer reviewers' comments and the authors' responses online. We also post the versions of the paper that were used during peer review. These are the versions that the peer review comments apply to.

The versions of the paper that follow are the versions that were submitted during the peer review process. They are not the versions of record or the final published versions. They should not be cited or distributed as the published version of this manuscript.

BMJ Open is an open access journal and the full, final, typeset and author-corrected version of record of the manuscript is available on our site with no access controls, subscription charges or pay-per-view fees (http://bmjopen.bmj.com).

If you have any questions on BMJ Open's open peer review process please email info.bmjopen@bmj.com

BMJ Open

Family-focused intervention to promote adolescent mental health and well-being in Moldova and North Macedonia (FLOURISH): Feasibility study protocol

Journal:	*BMJ Open*
Manuscript ID	bmjopen-2023-080400
Article Type:	Protocol
Date Submitted by the Author:	29-Sep-2023
Complete List of Authors:	Shenderovich, Yulia; Cardiff University, Centre for the Development, Evaluation, Complexity and Implementation in Public Health Improvement (DECIPHer), School of Social Sciences; Cardiff University, Wolfson Centre for Young People's Mental Health<br>Piolanti, Antonio; University of Klagenfurt, Institute of Psychology<br>Babii, Viorel; Asociatia Obsteasca Sanatate Pentru Tineri (Health for Youth Association)<br>Calovska-Hertzog, Nevena; Singidunum University, Department for Psychology, Faculty for Media and Communication; AST Centre for Education<br>Evans, Rhiannon; Cardiff University, Centre for the Development, Evaluation, Complexity and Implementation in Public Health Improvement (DECIPHer), School of Social Sciences<br>Heinrichs, Nina; Bielefeld University, Department of Psychology<br>Isakov, Anita Burgund; University of Belgrade, Faculty of Political Sciences, Department of Social Policy and Social Work<br>Lesco, Galina; Asociatia Obsteasca Sanatate Pentru Tineri (Health for Youth Association)<br>Moore, Graham; Cardiff University, Centre for the Development, Evaluation, Complexity and Implementation in Public Health Improvement (DECIPHer), School of Social Sciences; Cardiff University, Wolfson Centre for Young People's Mental Health<br>Mueller, Janina; University of Klagenfurt, Institute of Psychology<br>Raleva, Marija; Institute for Marriage, Family and Systemic Practice—ALTERNATIVA; Ss Cyril and Methodius University in Skopje, Department of Child and Adolescent Psychiatry<br>Shimbov, Bojan; Jaume I University Department of Economics, Instituto de Economía Internacional<br>Simon, Judith; Medical University of Vienna, Department of Health Economics, Center for Public Health; Oxford University, Department of Psychiatry, University of Oxford, Warneford Hospital<br>Waller, Franziska; University of Klagenfurt, Institute of Psychology<br>Wienand, Dennis; Medical University of Vienna, Department of Health Economics, Center for Public Health<br>Foran, Heather; University of Klagenfurt, Institute of Psychology
Keywords:	Adolescents < Adolescent, MENTAL HEALTH, PUBLIC HEALTH

SCHOLARONE™
Manuscripts

Family-focused intervention to promote adolescent mental health and well-being in Moldova and North Macedonia (FLOURISH): Feasibility study protocol

Yulia Shenderovich [1,2], Antonio Piolanti [3], Viorel Babii [4], Nevena Calovska-Hertzog [5,6], Rhiannon Evans [1], Nina Heinrichs [7], Anita Burgund Isakov [8], Galina Lesco [4], Graham Moore [1,2], Janina Mueller [3], Marija Raleva [9,10], Bojan Shimbov [11], Judit Simon [12,13], Franziska Waller [3], Dennis Wienand [12], Heather M. Foran [3]

1. Centre for the Development, Evaluation, Complexity and Implementation in Public Health Improvement (DECIPHer), School of Social Sciences, Cardiff University, Maindy Road, Cathays, Cardiff, CF24 4HQ, UK
2. Wolfson Centre for Young People's Mental Health, Cardiff University, Maindy Road, Cathays, Cardiff, CF24 4HQ, UK
3. Institute of Psychology, University of Klagenfurt, Universitaetstrasse 99, 9020 Klagenfurt am Woerthersee, Klagenfurt, Austria
4. Asociatia Obsteasca Sanatate Pentru Tineri (Health for Youth Association), Strada Socoleni 19, Chisinau 2020, Moldova
5. Department for Psychology, Faculty for Media and Communication, Singidunum University, Karadjordjeva 65, Belgrade, 11000, Serbia
6. AST Centre for Education, Djordja Kratovca 67, Belgrade, 11000, Serbia
7. Department of Psychology, Bielefeld University, Universitätsstrasse 25, 33615 Bielefeld, Germany
8. Faculty of Political Sciences, Department of Social Policy and Social Work, University of Belgrade, Jove Ilica 165, Belgrade, 11000, Serbia
9. Institute for Marriage, Family and Systemic Practice—ALTERNATIVA, Buld. Jane Sandanski 65, Skopje, 1000, North Macedonia
10. Department of Child and Adolescent Psychiatry, St. Cyril and Methodius University, Skopje, North Macedonia
11. Instituto de Economía Internacional, University Jaume I Castellon, Av. Vicent Sos Baynat, s/n 12071 Castellón de la Plana, Spain
12. Department of Health Economics, Center for Public Health, Medical University of Vienna, Kinderspitalgasse 15, 1090, Vienna, Austria
13. Department of Psychiatry, University of Oxford, Warneford Hospital, Oxford, OX3 7JX, UK

Yulia Shenderovich: ORCID 0000-0002-0254-3397; shenderovichy@cardiff.ac.uk;
Antonio Piolanti: ORCID 0000-0002-0613-0696; Antonio.Piolanti@aau.at
Viorel Babii: viorel.babii@gmail.com
Nevena Calovska-Hertzog: nevena.calovska@gmail.com
Rhiannon Evans: ORCID 0000-0002-0239-6331 ; EvansRE8@cardiff.ac.uk
Nina Heinrichs: ORCID 0000-0002-8301-5798 ; nina.heinrichs@uni-bielefeld.de
Anita Burgund Isakov: ORCID 0000-0002-4547-4934 ; anita.burgund@gmail.com
Galina Lesco: galina.lesco@gmail.com
Graham Moore: ORCID 0000-0002-6136-3978 ; MooreG@cardiff.ac.uk
Janina Mueller: ORCID 0000-0002-5005-2218 Janina.Mueller@aau.at
Marija Raleva: ORCID 0000-0002-5636-2612 ; marijaraleva@gmail.com
Bojan Shimbov: ORCID 0000-0002-0316-2430 ; shimbov@uji.es
Judit Simon : ORCID 0000-0001-9279-8627 ; judit.simon@meduniwien.ac.at
Franziska Waller: ORCID 0000-0002-4576-4325 ; Franziska.Waller@aau.at
Dennis Wienand : ORCID 0000-0002-5183-1352 ; dennis.wienand@meduniwien.ac.at

For peer review only - http://bmjopen.bmj.com/site/about/guidelines.xhtml

Heather M. Foran : ORCID 0000-0001-6659-5500; heather.foran@aau.at

Corresponding author: Yulia Shenderovich; address: spark, Maindy Road, Cathays, Cardiff, CF24 4HQ, UK; phone: +447774955977; email: shenderovichy@cardiff.ac.uk

Keywords: adolescents, parenting, mental health, adaptation, implementation science

For peer review only

ABSTRACT

Introduction

FLOURISH project will adapt, implement, and evaluate a programme to support adolescent mental health and well-being through strategies, such as strengthening parenting practices, adolescent-caregiver relationships, adolescent and parent socioemotional skills, and social support.

Methods and analysis

The project will focus on adolescents aged 10-14 and their caregivers in North Macedonia and Moldova. The countries were selected based on implementation readiness of two organisations and a need for accessible evidence-informed services to help mitigate health risks due to economic, social, and political challenges. Parenting for Lifelong Health (PLH) for Parents and Teens is a family-based programme developed for low-resource settings. PLH has been adapted with input from advisory groups. The programme includes additional components to strengthen impacts on adolescents: adolescent mental health tools, based on UNICEF's Helping Adolescents Thrive, adolescent peer support, and participation booster.

This pilot is first of three study phases. The pilot will be a feasibility testing of the adapted intervention and the assessment and implementation procedures to determine further refinements. The pilot will examine if the adapted programme is acceptable for adolescents, their families, and providers; explore contextual factors relevant to embedding this programme into longer-term scale-up; whether the programme can be delivered with fidelity and participation; whether the participants report changes in adolescent emotional and behaviour problems, well-being, and other outcomes; and whether the study tools are feasible and appropriate. Pre-post adolescent and caregiver questionnaires will provide outcome data. Process evaluation will include attendance and fidelity data, and focus groups. We will examine delivery cost and resource requirements.

Ethics and dissemination

The study was approved at the University of Klagenfurt (Austria), Medical Faculty at St. Cyril and Methodius University (North Macedonia), and National Committee of Ethical Expertise for Clinical Trials (Moldova). Through stakeholder engagement and dissemination, FLOURISH will advance scale-up of open-source family interventions.

Registration details

Trial registration: ID101095528; project page: https://www.flourish-study.org/about.html

For peer review only - http://bmjopen.bmj.com/site/about/guidelines.xhtml

ARTICLE SUMMARY

Strengths and limitations of this study

- This pilot study is embedded in a project designed to address a gap in evidence-informed programmes to promote adolescent mental health and well-being in North Macedonia and Moldova, at a time when migration, threat of war, and economic challenges exert particular pressures on adolescents and their families.
- The study is adapting an open-access intervention to new contexts, with a focus on scalability, to identify a model for adolescent-parent programmes that can be embedded in long-term delivery to provide a high-quality and accessible services in both countries.
- While the pilot evaluation has a relatively small sample size, it will provide initial results on the feasibility of the intervention, its implementation and evaluation; effectiveness will be evaluated in further phases with a randomised design.

For peer review only

For peer review only - http://bmjopen.bmj.com/site/about/guidelines.xhtml

Family-focused intervention to promote adolescent mental health and well-being in Moldova and North Macedonia (FLOURISH): Feasibility study protocol

Word count: 4,179

INTRODUCTION

Adolescence is a period of transition and transformation, when one can acquire and strengthen factors contributing to lifelong health (Ross et al., 2020). Mental health problems during early adolescence are a global concern as this is when about half of mental health problems emerge (Rice et al., 2017). Stressful experiences in early adolescence are more strongly associated with a shorter total life span than such stressors in other phases of childhood, suggesting early adolescence is a sensitive period, making it a crucial time for providing additional support (Falconi et al., 2014).

Having supportive relationships is associated with good health (Holt-Lunstad, 2018). The relationships adolescents have with their parents can remain influential, even as peers become increasingly important. Parents or caregivers are defined broadly to include any caregiver providing significant care to an adolescent, not limited to a biological parent (UNICEF, 2018). Parenting programmes focusing on the adolescent-caregiver relationships and parenting practices are recommended to support adolescent mental health (Pedersen et al., 2019; UNICEF, 2021; WHO, 2022). Furthermore, the WHO Guidelines on mental health promotive and preventive interventions for adolescents also recommend interventions for adolescents' interpersonal and emotional regulation skills (WHO, 2020).

A meta-analysis found no significant difference in effectiveness between transported and homegrown parenting interventions for reducing disruptive child behaviour (Leijten et al., 2016). However, culturally adapted parenting interventions, both homegrown and transported, were more effective than non-adapted interventions (Gardner et al., 2015). Adapting interventions can help maintain effectiveness while avoiding the cost of developing new interventions (Moore et al., 2021). Although majority of adolescents live in low- and middle-income countries (LMICs), research with adolescents in most LMICs is very limited (Skeen et al., 2019), so evidence often has to be adapted from elsewhere.

The Family-Focused Adolescent & Lifelong Health Promotion (FLOURISH) project (2023-2026) is focused on adapting, optimising, and evaluating an intervention package for adolescents aged 10-14 and their caregivers in the Republic of North Macedonia and the Republic of Moldova (henceforth North Macedonia and Moldova). The intervention package aims to support adolescent mental health and well-being through building adolescents' and caregivers' skills, such as problem-solving and emotional regulation, strengthening social support, adolescent-caregiver relationships and communication, and improving parenting practices.

Moldova and North Macedonia were selected based on high risks for adolescent mental health, including adverse childhood experiences and poverty; lack of evidence-informed and accessible provision of prevention programmes for families with adolescents, paired with high levels of implementation readiness in two health networks. North Macedonia and Moldova are middle-income countries, among the poorest in Europe (World Bank, 2022). Both have experienced rapid social, political, and economic transformation since 1990s following the fall of Communism.

Studies found high rates of physical punishment, emotional violence and neglect against children in both countries (Sebre et al., 2004; WHO, 2013, 2018). Work migration often leads caregivers to spend time away from children, which can hamper communication (UNICEF, 2018). Young people also face limited work opportunities. Existing challenges

For peer review only - http://bmjopen.bmj.com/site/about/guidelines.xhtml

were magnified by the coronavirus pandemic (Bhatia et al., 2020, 2021; Katz et al., 2021), and the war in Ukraine. There is stigma around help-seeking, particularly regarding mental health and well-being (Dlouhy, 2014). A recent report found that in this region support services for caregivers of adolescents, such as parenting workshops or support groups, are scarce, and highlighted the need for more such services (UNICEF, 2018), which our project aims to help address.

This protocol describes the adapted intervention, designed to promote adolescent mental health and well-being in Moldova and North Macedonia, and introduces the FLOURISH feasibility pilot (2023-24), which will inform the next phases of a larger study by providing information for further refinement of the intervention, and its implementation and evaluation procedures.

METHODS

Research questions and frameworks

FLOURISH is shaped by the Multiphase Optimization Strategy (MOST) framework. MOST is designed to optimise an intervention package within the key constraints in three phases – Preparation, Optimization, and Evaluation (Collins, 2018; Guastaferro & Collins, 2021). This pilot study corresponds to the Preparation phase, focused on preparing and piloting the intervention and its evaluation. Following the pilot, a factorial trial will be conducted to select the most effective and efficient treatment package (Optimization phase). In the Evaluation phase, the revised programme package will be tested in an implementation-effectiveness randomised controlled trial.

Next, we discuss the research questions and additional frameworks and guidelines used in answering them (see Table 1 for an overview).

1. Is the adapted programme acceptable for adolescents, their caregivers, and staff in North Macedonia and Moldova, and what further adaptation are needed?

Programme materials have been adapted prior to piloting and will be revised throughout the project. FLOURISH draws on ADAPT adaptation guidance, which emphasises improving intervention fit with a new context while preserving key intervention functions (Evans et al., 2021; Moore et al., 2021).

2. What contextual factors may influence embedding of the programme into sustainable delivery and funding mechanisms in North Macedonia and Moldova?

FLOURISH is guided by the principle of “beginning with the end in mind” in ExpandNET/ World Health Organization (WHO) guidance (Ghiron et al., 2021). We will adapt the programme alongside developing a scaling-up strategy. This includes building capacity for scale-up and making choices to support institutionalization and expansion to more settings.

3. Can the adapted programme be delivered with high fidelity and family participation?

The pilot includes a process evaluation, guided by UK MRC process evaluation guidance (Moore et al., 2015), which focuses on implementation processes, potential mechanisms of impact, and contextual factors. To organise our understanding of the country context, we are using the Context and Implementation of Complex Interventions (CICI) framework (Pfadenhauer et al., 2017).

For peer review only - http://bmjopen.bmj.com/site/about/guidelines.xhtml

4. What are the changes, if any, reported by the adolescents and caregivers on the primary outcomes of adolescent emotional problems, behaviour problems, and well-being, and on the secondary outcomes for adolescents and caregivers?

Primary programme targets are adolescent mental health and well-being. The pilot study will assess pre-post changes in the intervention outcomes reported by adolescents and their caregivers, following lessons learned in a previous pilot feasibility study, on which this project builds (Frantz et al., 2019; Jansen et al., 2021).

5. Are the study tools and their translations feasible and appropriate?

The pilot will examine the feasibility and preliminary psychometric performance of the outcome measures and their translations. A rigorous translation and evaluation process will be followed. The pilot will provide insights on the feasibility of the procedures and tools, and inform the next phase of the project, including which tools are retained, removed, or modified.

Table 1. Overview of the study design

Data	Participants	Research question	Framework
Advisory group consultations; focus groups	Adolescents, caregivers, staff, and (only for advisory groups) external professionals	1	ADAPT; process evaluation guidelines
Interviews	External professionals	2	CICI; ExpandNet
Attendance registers; observations; focus groups	Staff	3	Process evaluation guidelines
Questionnaires	Adolescents and caregivers	4	MOST
Think-aloud interviews; questionnaires	Adolescents and caregivers	5	N/A

Study sites

The study is conducted in two country-wide health networks, Institute for Marriage, Family and Systemic Practice (ALTERNATIVA) and Health for Youth Association. ALTERNATIVA is a network of psychologists, social workers, and family therapists in North Macedonia. Health for Youth Association in Moldova is an organisation which supports the activity of publicly funded youth-friendly clinics offering prevention and treatment in youth sexual and reproductive health, mental health, substance use, and violence prevention. Both organisations work with caregivers and adolescents, have delivered Parenting for Lifelong Health (PLH) programs, and participated in evaluations (Frantz et al., 2019).

Study design

For peer review only - http://bmjopen.bmj.com/site/about/guidelines.xhtml
For peer review only

Intervention programme

The core programme examined in FLOURISH is PLH for Parents and Teens. It is a group behavioural programme (Cluver et al., 2018; Lachman et al., 2019), based on social learning theory (Bandura, 1977). PLH was developed for LMICs in collaboration with UNICEF, WHO, and other international organisations and universities. Programme materials are focused on strengthening psychosocial skills and relationships, and are freely available online: https://www.who.int/teams/social-determinants-of-health/parenting-for-lifelong-health.

PLH programmes, primarily for caregivers with young children (aged 2-9), have been evaluated in multiple studies, including completed and ongoing trials in South Africa, Philippines, El Salvador, Lesotho, North Macedonia, Moldova, Romania, Thailand, Uganda, and Zambia. Evaluations reported high levels of engagement by families, practitioners, and other stakeholders. A pilot feasibility study and two large randomised trials in North Macedonia, Moldova, and Romania found improvements in parenting behaviours and child mental health (Jansen et al., 2021; Lachman et al., 2019; Foran et al. forthcoming; Heinrichs et al. forthcoming). PLH for Parents and Teens (aged 10-17) has been evaluated in South Africa in two pre-post studies and an RCT (552 families, 40 clusters), with an embedded process evaluation (Cluver et al., 2018; Shenderovich et al., 2019). At 5-9 months after the 14-week intervention, caregivers in the intervention group, but not adolescents, reported lower rates of violence towards their adolescents and corporal punishment, and both caregivers and adolescents reported greater involved parenting and supervision of the adolescent by the caregiver (primary outcomes). There was no intervention effect detected on adolescent depression, suicidality, and externalising behaviour – however, these secondary outcomes were not the focus of the intervention.

Adaptation of the intervention

The adapted programme is drawing on the PLH manual used in the South African evaluation and on other programme versions. The initial adaptations have been informed by the input of advisory groups and expert interviews (described below), team's clinical expertise, and input from the programme developers. The adapted programme will be tested in the pilot (see the initial programme theory in Figure 1, to be revised throughout the project).

To support scalability, the programme was condensed into six group sessions, following on an introductory meeting with the caregiver and adolescent. As in the original model, sessions in FLOURISH will be delivered to a group of 10 adolescent-caregiver pairs. Each two-hour weekly session starts jointly, with a circle share, home activity discussion, agenda overview, physical exercises and games. Next, in the core lesson, a new skill is introduced and practiced, on topics developmentally relevant to adolescence. For the core lesson, the group is often split up into adolescent and caregiver parallel sessions to allow participants to share openly (see Table 2). After discussing the core lesson within the respective peer groups, participants come together and are encouraged to share the summary of their separate discussions in the joint group, and then further discuss lessons learned at home. Participants are given home practice tasks to do between the sessions. For participants who miss a session, facilitators offer a brief catch-up call or meeting. Facilitators and supervisors receive brief training in the programme and facilitation skills. Supervisors are trained in supervision. The programme is delivered by two facilitators per group, coached weekly by supervisors to promote quality of delivery. Given the focus on scalability, staff are not required to have a specific degree.

For peer review only - http://bmjopen.bmj.com/site/about/guidelines.xhtml

Table 2. *Adapted intervention structure using PLH material*

Week	Topic	Caregiver & adolescents
Pre-program	Individual meeting or call with facilitators to introduce the programme	Joint
1	Introducing the programme & ground rules. Psychoeducation about transitioning from a child to an adolescent: expectations, norms, and developmental stages	Separate
2	Relationship skills. Building a positive relationship through spending time together & praising each other	Joint
3	Talking about emotions and sensitive topics (sexuality, body changes, and intimate relationships)	Separate
4	Coping with difficult feelings (anger and stress)	Separate
5	Solving problems together as a family. How to communicate and solve problems around disagreements without conflict	Separate
6	Establishing rules and routines	Separate

To strengthen the impacts on adolescent outcomes, the adaptation of the programme involves supplementary components. Three components have been combined with the PLH programme into an intervention package: adolescent mental health tools, adolescent peer support, and adolescent participation booster.

Adolescent mental health tools. To strengthen adolescent mental health, we draw on the Helping Adolescents Thrive toolkit (HAT), designed to promote positive mental health, prevent mental health problems, and reduce engagement in self-harm and risk behaviours (UNICEF and WHO, 2021). This toolkit was developed based on a systematic review of universally delivered psychosocial interventions for adolescents (Skeen et al., 2019), conducted to inform WHO Guidelines (WHO, 2020). The HAT materials will be delivered as six comics chapters, a workbook for adolescents and leaflets for caregivers, including, respectively, tips for adolescents to share their thoughts and feelings with their caregivers and questions caregivers can ask adolescents. The aim of these materials is to support adolescents and caregivers to communicate about adolescent mental health and well-being.

Adolescent peer support. Even brief interventions can foster friendships and social connections (Echols & Ivanich, 2021; Veenstra & Laninga-Wijnen, 2022). Since parenting programmes have traditionally focused on parents, they have demonstrated positive impacts on social support experienced by parents but not by adolescents, including in the previous trial of PLH for Parents and Teens (Cluver et al., 2018). The facilitators will pair up adolescents in the group for facilitating peer support and social connection. The PLH programme already includes peer pairings, but its potential effects have not yet been evaluated, so more emphasis will be placed on pairing up adolescents, and this will be tested as a separate component in the Optimization phase.

Adolescent participation booster. Although parenting programmes have been found acceptable in diverse settings, there is incomplete attendance (Butler & Titus, 2015; Chacko et al., 2016; Gonzalez et al., 2018). While many studies have explored parental attendance for parents of younger children (Jansen et al., 2022; Foran et al., forthcoming), there is limited research on enhancing engagement of adolescents and their caregivers as programmes rarely included adolescents. The South African trial found 50-64% session attendance (Shenderovich et al., 2018). We will investigate whether an adolescent participation booster promotes attendance.

Advisory groups. Intervention adaptations have been informed by advisory group consultations and interviews. Advisory groups of adolescents, caregivers, implementation

For peer review only - http://bmjopen.bmj.com/site/about/guidelines.xhtml

organisation staff, and other professional experts (6-8 people per group) were formed in North Macedonia and Moldova to advise on programme adaptations and the study findings. Consultations will continue throughout the study, with key discussion points captured by a note-taker. Adolescents are eligible if they are aged 10-14 when joining the advisory group and provide adolescent assent and caregiver consent. For caregivers, staff, and other professionals, members need to be 18 or older and provide consent. For the caregiver advisory group, participants need to be caregivers of adolescents aged 10-17 (extended age range to facilitate recruitment). The groups aim to include individuals with experience of PLH or other family programmes and individuals who represent diverse perspectives (e.g., male and female caregivers, ethnic minorities). Implementation staff groups consist of ALTERNATIVA and Health for Youth Association staff. External professional experts include representatives from Ministries of Health, social services, youth, universities, research organisations, healthcare financing agencies, local NGOs, community, and international organisations implementing programmes for young people.

Advisory group analysis. Summary notes were combined into a matrix, using thematic framework and content analysis (Gale et al., 2013; Heath et al., 2012). We examined similarities and differences within and between stakeholder groups and countries. Advisory group participants received a summary for feedback as a means of validation.

Expert interviews. In each country, ten semi-structured audio-recorded interviews with professional experts took place to map the contextual facilitators, and barriers to scaling up a programme package prepared by the FLOURISH project. We drew on recommendations for qualitative sample sizes in Hennink & Kaiser (2022), Krueger (2014).

Interview analysis. We are using thematic and framework analysis for the transcripts. The CICI framework provides initial coding domains, with country-specific domains generated from the data. The analysis is conducted in two stages – first, a rapid synthesis to inform the intervention adaptations for the pilot (completed); second, a detailed analysis, generating a conceptual map of key contextual factors, presented as a narrative summary (ongoing).

Insert Figure 1 here

Figure 1. Initial programme theory

Piloting of the intervention

The pilot will test the adapted intervention in a pre-post uncontrolled study with an outcome and process evaluation, described below.

Participants and recruitment. The pilot will include three intervention groups per country, so the target sample size is 60 adolescents and 60 caregivers (60 dyads). Recruitment will involve disseminating information through online and print materials, word-of-mouth, and via the existing clients of each network. At least one caregiver and adolescent per family will participate in the study. Inclusion criteria for caregivers are age 18 or older at baseline assessment; primary caregiver responsible for the care of an adolescent 10-14, resident in the same household at least four nights a week in the previous month; able to speak one of the local languages, in which the programme will be offered; agreement to participate in the programme; provision of consent for self and adolescent to participate in the study. Inclusion criteria for adolescents: age 10-14 at baseline assessment; assent to participate; caregiver consent.

The caregiver can be any adult who is primarily responsible for looking after the adolescent and available to participate. In cases where there is more than one caregiver who is available, they will need to agree who will be the primary and secondary participant. Similarly, if two adolescents in the same age range in a family would like to join, both can

For peer review only - http://bmjopen.bmj.com/site/about/guidelines.xhtml

join. Everyone who joins the study, both primary and secondary participants, will be invited to complete assessments.

Taking a proportionate universalism approach (Segrott et al., 2022), all families will be eligible, but we will make greater efforts to include more vulnerable groups. We conceptualise our approach as selected prevention, targeted to individuals whose risk is above average, without screening (National Research Council and Institute of Medicine, 2009). FLOURISH has no exclusion criteria. While introducing the project, the researchers will guide the participants through a consent form that asks whether they are currently experiencing acute distress, or a mental or physical health condition that would interfere with participation. The judgement will be made by the potential participants. If they decide they are not able to take part, the research team will follow up and provide referrals for other services (see also Ethics section).

To deliver the programme, facilitators and supervisors will be recruited from existing staff and networks of the implementing organisations. Inclusion criteria for facilitators are age 18 or older; participation in training; agreement to deliver the programme; provision of study consent. For supervisors: age 18 or older; participation in training; agreement to provide supervision; provision of consent.

Pre-post outcomes. Outcome data collection with families will be done primarily using computer-assisted self-interviewing questionnaires on tablets. Trained assessors will check participant eligibility as part of the informed consent procedure and assist adolescents and caregivers with completing the questionnaires. See Table 2 for the primary outcome measures and Table 1s (supplementary) for secondary outcome measures (pre-registered in clinical trials registry; ID101095528).

Outcome measures without available official translations will be adapted and translated following best practices (Reichenheim & Bastos, 2021). First, a cultural evaluation (relevance, appropriateness) of each item and forward-translation will be conducted by a professional with mental health experience. Second, a back-translation to English will be carried out by another translator. Third, an additional translator will compare the translations and assess correspondence between semantic equivalence and words, highlighting any discrepancies. A final translation will be produced in a meeting between the translators. All translators will be bilingual.

Think-aloud interviews will be conducted for the primary outcome measures that have not been previously used with adolescents or in the study countries (RCADS, WHO-5), and for the health economic measures that will be used in the Optimization and Evaluation phases – EQ-5D-5L (Herdman et al., 2011), EQ-5D-Y (Wille et al., 2010), OxCAP-MH (Simon et al., 2018; Vergunst et al., 2017), PECUNIA RUM (Pokhilenko et al., 2023). Think-aloud interviews will be conducted after post-test assessments and explore item comprehension (10 interviews per country with adolescents and caregivers in Romanian and Macedonian).

Table 2. Primary Outcomes

Construct	Respondent	Measure
Adolescent emotional problems	Adolescent	- The Revised Child Anxiety and Depression Scales (RCADS), anxiety and depression subscales (Ebetsuani et al., 2012; Krause et al., 2021)<br>- Child Behaviour Checklist 6-18, internalising subscale (Achenbach & Rescorla, 2001)<br>- Youth Self-Report 11-18, internalising subscale (Achenbach & Rescorla, 2001)
Adolescent	Adolescent	Youth Self-Report, rule-breaking and aggressive behaviour

For peer review only - http://bmjopen.bmj.com/site/about/guidelines.xhtml

Construct	Respondent	Measure
behaviour problems	Caregivers	Child Behaviour Checklist, rule-breaking and aggressive behaviour
Adolescent well-being	Adolescent	World Health Organization-Five Well-Being Index (WHO-5) (Topp et al., 2015; WHO, 1998)

Pre-post outcomes analysis. We will examine descriptive and preliminary psychometric statistics, such as internal consistencies. Intent-to-treat analyses will examine pre-post changes in adolescent and caregiver outcomes. We hypothesise that adolescent emotional problems and adolescent behaviour problems, both in self- and caregiver report, will be significantly reduced at the post-assessment; adolescent well-being will be significantly increased at the post-assessment, with medium to large effect sizes (Cohen's d = > .5). We also will examine pre-post change in secondary outcomes and hypothesise small to medium effect sizes. Missing data will be addressed using full information maximum likelihood or multiple imputation methods.

Process evaluation. Facilitators will collect weekly attendance registers. Programme supervisors will conduct fidelity assessments. One session per facilitator will be observed live or video-recorded and assessed using the recording with the Facilitator Assessment Tool (Martin et al., 2023). Facilitators and supervisors will complete questionnaires to provide information on demographics and previous experience, and to collect pre-post data on staff well-being and parenting stress (for staff who are caregivers). The sample sizes are determined by the delivery model and the number of families in the intervention study.

Process evaluation analysis. We will use descriptive statistics, summarizing enrolment, attendance, fidelity, and facilitator characteristics. We will check variation in attendance by participant baseline characteristics (e.g., gender) and by intervention delivery characteristics (e.g., study site) to surface any emerging inequalities. We will explore whether attendance varied across sessions. We will explore staff responses to the pre-post well-being questionnaires using descriptives and paired t-tests. This will help inform, for example, whether additional support is needed if staff well-being had reduced.

Intervention costs. Facilitators and supervisors will complete questionnaires to capture the time and other resources spent preparing for, delivering, and following up on intervention activities. They will complete one form for the initial training and weekly forms designed to separate the resources required for the intervention versus the study.

Costs analysis. We will assess if weekly surveys are feasible. We will conduct descriptive analyses, summarizing information on time, money, and other resources used. Resource use information will be costed with country-specific unit costs collected primarily for the study to provide overall cost data. Cost information will be used to inform scale-up planning, and, in later phases, for programme cost-effectiveness analyses.

Focus groups. To inform further programme adaptations and answer process evaluation questions about the context, how the programme was implemented, and its mechanisms of change, we will conduct participant focus groups. A sub-sample of the adolescents, caregivers, and intervention staff will be recruited to participate in post-program audio-recorded focus groups (6-8 people/group, minimum 3 groups/country). We will aim to select participants with diverse backgrounds and experiences.

Focus group analysis. Focus group transcripts will be analysed using thematic and framework analysis, with codes developed both based on the initial questions and unexpected insights.

For peer review only - http://bmjopen.bmj.com/site/about/guidelines.xhtml

ETHICS

An ethical self-assessment for Horizon Europe was reviewed by the European Commission, and the project was cleared for ethics. The study was approved by the Institutional Review Board for Research Ethics at the University of Klagenfurt, Austria (coordinating site), the Ethical Commission for Human Research at the Medical Faculty at St. Cyril and Methodius University, North Macedonia, and National Committee of Ethical Expertise for Clinical Trials of the Ministry of Health, Moldova.

Participants will provide written assent (adolescents) or consent (adults). Adolescents and caregivers will receive reimbursements for transport costs, and thank-you vouchers for data collection activities, worth ~ €10. All staff will be trained to identify and handle potential distress in participants with detailed safety procedures and referral processes. Adverse event monitoring will help detect any potential harm caused by the research or the intervention. Any serious adverse events will be investigated and reported to the ethics committees and the data and safety monitoring board to consider potential changes in the project.

DISSEMINATION AND EXPECTED RESULTS

The FLOURISH project emphasises dissemination to stakeholders. Dissemination will include meetings, communication with media, and through print and online channels, including existing networks, FLOURISH website and social media. The focus of dissemination is Moldova and North Macedonia, and we will also be engaging stakeholders in the Eastern Europe region, working with family and adolescent health associations, NGOs, government, and international agencies.

This pilot will provide insights on the feasibility of the intervention, its implementation and evaluation, and thus inform the factorial trial planned as the next research phase, guiding which changes need to be made to the intervention, implementation, and evaluation procedures. More broadly, this project will provide insights on how best to adapt and scale up a programme targeting family relationships and adolescent mental health at a time when economic problems, interpersonal and intergroup conflict, the threat of a wider war, and other issues contribute to increased stress for both adolescents and adults. We will draw on a set of frameworks and global health best practices (Patel et al., 2018), such as using open-access interventions that involve stakeholders in all stages of intervention adaptation and evaluation.

For peer review only - http://bmjopen.bmj.com/site/about/guidelines.xhtml

REFERENCES

Achenbach, T. M., & Rescorla, L. (2001). *The Manual for the ASEBA School-Age Forms & Profiles.*

Bandura, A. (1977). *Social learning theory*. General Learning Press.

Bhatia, A., Fabbri, C., Cerna-Turoff, I., Tanton, C., Knight, L., Turner, E., Lokot, M., Lees, S., Cislaghi, B., Peterman, A., Guedes, A., & Devries, K. (2020). COVID-19 response measures and violence against children. *Bulletin of the World Health Organization*, *98*(9), 583-583A. https://doi.org/10.2471/BLT.20.263467

Bhatia, A., Fabbri, C., Cerna-Turoff, I., Turner, E., Lokot, M., Warria, A., Tuladhar, S., Tanton, C., Knight, L., Lees, S., Cislaghi, B., Bhabha, J., Peterman, A., Guedes, A., & Devries, K. (2021). Violence against children during the COVID-19 pandemic. *Bulletin of the World Health Organization*, *99*(10), 730–738. https://doi.org/10.2471/BLT.20.283051

Butler, A. M., & Titus, C. (2015). Systematic Review of Engagement in Culturally Adapted Parent Training for Disruptive Behavior. *Journal of Early Intervention*, *37*(4), 300–318. https://doi.org/10.1177/1053815115620210

Carlsen, B., & Glenton, C. (2011). What about N? A methodological study of sample-size reporting in focus group studies. *BMC Medical Research Methodology*, *11*(1), 26. https://doi.org/10.1186/1471-2288-11-26

Chacko, A., Jensen, S. A., Lowry, L. S., Cornwell, M., Chimklis, A., Chan, E., Lee, D., & Pulgarin, B. (2016). Engagement in behavioral parent training: Review of the literature and implications for practice. *Clinical Child and Family Psychology Review*, *19*(3), 204–215. https://doi.org/10.1007/s10567-016-0205-2

Chorpita, B. F., Moffitt, C. E., & Gray, J. (2005). Psychometric properties of the Revised Child Anxiety and Depression Scale in a clinical sample. *Behaviour Research and Therapy*, *43*(3), 309–322. https://doi.org/10.1016/j.brat.2004.02.004

Cluver, L. D., Meinck, F., Steinert, J. I., Shenderovich, Y., Doubt, J., Herrero Romero, R., Lombard, C. J., Redfern, A., Ward, C. L., Tsoanyane, S., Nzima, D., Sibanda, N., Wittesaele, C., De Stone, S., Boyes, M. E., Catanho, R., Lachman, J. M., Salah, N., Nocuza, M., & Gardner, F. (2018). Parenting for Lifelong Health: A pragmatic cluster randomised controlled trial of a non-commercialised parenting programme for adolescents and their families in South Africa. *BMJ Global Health*, *3*(1), e000539. https://doi.org/10.1136/bmjgh-2017-000539

Collins, L. M. (2018). *Optimization of Behavioral, Biobehavioral, and Biomedical Interventions* (Vol. 22, Issue 1). Springer International Publishing. https://doi.org/10.1007/978-3-319-72206-1

Dlouhy, M. (2014). Mental health policy in Eastern Europe: A comparative analysis of seven mental health systems. *BMC Health Services Research*, *14*. https://doi.org/10.1186/1472-6963-14-42

Echols, L., & Ivanich, J. (2021). From “Fast Friends” to True Friends: Can a Contact Intervention Promote Friendships in Middle School? *Journal of Research on Adolescence*, *31*(4), 1152–1171. https://doi.org/10.1111/jora.12622

Evans, R. E., Moore, G., Movsisyan, A., & Rehfuess, E. (2021). How can we adapt complex population health interventions for new contexts? Progressing debates and research priorities. *Journal of Epidemiology and Community Health*, *75*(1), 40–45. https://doi.org/10.1136/jech-2020-214468

Falconi, A., Gemmill, A., Dahl, R. E., & Catalano, R. (2014). Adolescent experience predicts longevity: Evidence from historical epidemiology. *Journal of Developmental Origins of Health and Disease*, *5*(3), 171–177. https://doi.org/10.1017/S2040174414000105

For peer review only - http://bmjopen.bmj.com/site/about/guidelines.xhtml

Frantz, I., Foran, H. M., Lachman, J. M., Jansen, E., Hutchings, J., Băban, A., Fang, X., Gardner, F., Lesco, G., Raleva, M., Ward, C. L., Williams, M. E., & Heinrichs, N. (2019). Prevention of child mental health problems in Southeastern Europe: A multicentre sequential study to adapt, optimise and test the parenting programme "Parenting for Lifelong Health for Young Children", protocol for stage 1, the feasibility study. *BMJ Open*, *9*(1). https://doi.org/10.1136/bmjopen-2018-026684

Gale, N. K., Heath, G., Cameron, E., Rashid, S., & Redwood, S. (2013). Using the framework method for the analysis of qualitative data in multi-disciplinary health research. *BMC Medical Research Methodology*, *13*(1), 117. https://doi.org/10.1186/1471-2288-13-117

Gardner, F., Montgomery, P., & Knerr, W. (2015). Transporting evidence-based parenting programs for child problem behavior (age 3–10) between countries: Systematic review and meta-analysis. *Journal of Clinical Child & Adolescent Psychology*, *45*, 749–762. https://doi.org/10.1080/15374416.2015.1015134

Ghiron, L., Ramirez-Ferrero, E., Badiani, R., Benevides, R., Ntabona, A., Fajans, P., & Simmons, R. (2021). Promoting Scale-Up Across a Global Project Platform: Lessons from the Evidence to Action Project. *Global Implementation Research and Applications*, *1*(2), 69–76. https://doi.org/10.1007/s43477-021-00013-4

Gonzalez, C., Morawska, A., & Haslam, D. M. (2018). Enhancing Initial Parental Engagement in Interventions for Parents of Young Children: A Systematic Review of Experimental Studies. *Clinical Child and Family Psychology Review*, *21*(3), 415–432. https://doi.org/10.1007/s10567-018-0259-4

Guastaferro, K., & Collins, L. M. (2021). Optimization Methods and Implementation Science: An Opportunity for Behavioral and Biobehavioral Interventions. *Implementation Research and Practice*, *2*, 263348952110543. https://doi.org/10.1177/26334895211054363

Heath, G., Cameron, E., Cummins, C., Greenfield, S., Pattison, H., Kelly, D., & Redwood, S. (2012). Paediatric "care closer to home": Stakeholder views and barriers to implementation. *Health and Place*, *18*(5), 1068–1073. https://doi.org/10.1016/j.healthplace.2012.05.003

Hennink, M., & Kaiser, B. N. (2022). Sample sizes for saturation in qualitative research: A systematic review of empirical tests. *Social Science and Medicine*, *292*, 114523. https://doi.org/10.1016/j.socscimed.2021.114523

Herdman, M., Gudex, C., Lloyd, A., Janssen, M., Kind, P., Parkin, D., Bonsel, G., & Badia, X. (2011). Development and preliminary testing of the new five-level version of EQ-5D (EQ-5D-5L). *Quality of Life Research*, *20*(10), 1727–1736. https://doi.org/10.1007/s11136-011-9903-x

Holt-Lunstad, J. (2018). Why Social Relationships Are Important for Physical Health: A Systems Approach to Understanding and Modifying Risk and Protection. *Annual Review of Psychology*, *69*, 437–458. https://doi.org/10.1146/annurev-psych-122216-011902

Ivanova, M. Y., Achenbach, T. M., Rescorla, L. A., Dumenci, L., Almqvist, F., Bilenberg, N., Bird, H., Broberg, A. G., Dobrean, A., Döpfner, M., Erol, N., Forns, M., Hannesdottir, H., Kanbayashi, Y., Lambert, M. C., Leung, P., Minaei, A., Mulatu, M. S., Novik, T., … Verhulst, F. C. (2007). The generalizability of the Youth Self-Report syndrome structure in 23 societies. *Journal of Consulting and Clinical Psychology*, *75*(5), 729–738. https://doi.org/10.1037/0022-006X.75.5.729

Jansen, E., Frantz, I., Hutchings, J., Lachman, J., Williams, M., Taut, D., Baban, A., Raleva, M., Lesco, G., Ward, C., Gardner, F., Fang, X., Heinrichs, N., & Foran, H. M. (2021). Preventing child mental health problems in southeastern Europe: Feasibility study (phase 1 of MOST framework). *Family Process*, *August 2020*, 1–18.

For peer review only - http://bmjopen.bmj.com/site/about/guidelines.xhtml

https://doi.org/10.1111/famp.12720

Katz, I., Katz, C., Andresen, S., Bérubé, A., Collin-Vezina, D., Fallon, B., Fouché, A., Haffejee, S., Masrawa, N., Muñoz, P., Priolo Filho, S. R., Tarabulsy, G., Truter, E., Varela, N., & Wekerle, C. (2021). Child maltreatment reports and child protection service responses during COVID-19: Knowledge exchange among Australia, Brazil, Canada, Colombia, Germany, Israel, and South Africa. *Child Abuse and Neglect*, *116*(August 2020), 105078. https://doi.org/10.1016/j.chiabu.2021.105078

Krause, K. R., Chung, S., Adewuya, A. O., Albano, A. M., Babins-Wagner, R., Birkinshaw, L., Brann, P., Creswell, C., Delaney, K., Falissard, B., Forrest, C. B., Hudson, J. L., Ishikawa, S., Khatwani, M., Kieling, C., Krause, J., Malik, K., Martínez, V., Mughal, F., … Wolpert, M. (2021). International consensus on a standard set of outcome measures for child and youth anxiety, depression, obsessive-compulsive disorder, and post-traumatic stress disorder. *The Lancet Psychiatry*, *8*(1), 76–86. https://doi.org/10.1016/S2215-0366(20)30356-4

Krueger, R. A. (2014). *Focus groups: A practical guide for applied research*. Sage publications.

Lachman, J., Heinrichs, N., Jansen, E., Brühl, A., Taut, D., Fang, X., Gardner, F., Hutchings, J., Ward, C. L., Williams, M. E., Raleva, M., Båban, A., Lesco, G., & Foran, H. M. (2019). Preventing child mental health problems through parenting interventions in Southeastern Europe (RISE): Protocol for a multi-country cluster randomized factorial study. *Contemporary Clinical Trials*, *86*, 105855. https://doi.org/10.1016/j.cct.2019.105855

Lachman, J. M., Martin, M., Booij, A., Tsoanyane, S., & Majenga, N. (2019). *Parenting for Lifelong Health for Teens: Facilitator Assessment Tool Manual*.

Leijten, P., Melendez-Torres, G. J., Knerr, W., & Gardner, F. (2016). Transported versus homegrown parenting interventions for reducing disruptive child behavior: A multilevel meta-regression study. *Journal of the American Academy of Child and Adolescent Psychiatry*, *55*(7), 610–617. https://doi.org/10.1016/j.jaac.2016.05.003

Martin, M., Lachman, J. M., Murphy, H., Gardner, F., & Foran, H. (2023). The development, reliability, and validity of the Facilitator Assessment Tool: An implementation fidelity measure used in Parenting for Lifelong Health for Young Children. *Child: Care, Health and Development*, *49*(3), 591–604. https://doi.org/10.1111/cch.13075

Moore, G., Audrey, S., Barker, M., Bond, L., Bonell, C., Hardeman, W., Moore, L., O'Cathain, A., Tinati, T., Wight, D., & Baird, J. (2015). Process evaluation of complex interventions: Medical Research Council guidance. *British Medical Journal*, *350*, h1258. https://doi.org/10.1136/bmj.h1258

Moore, G., Campbell, M., Copeland, L., Craig, P., Movsisyan, A., Hoddinott, P., Littlecott, H., O'Cathain, A., Pfadenhauer, L., Rehfuess, E., Segrott, J., Hawe, P., Kee, F., Couturiaux, D., Hallingberg, B., & Evans, R. (2021). Adapting interventions to new contexts-the ADAPT guidance. *The BMJ*, *374*(fig 1). https://doi.org/10.1136/bmj.n1679

National Research Council and Institute of Medicine. (2009). Preventing Mental, Emotional, and Behavioral Disorders Among Young People. In *Preventing Mental, Emotional, and Behavioral Disorders Among Young People: Progress and Possibilities.* National Academies Press. https://doi.org/10.17226/12480

Patel, V., Saxena, S., Lund, C., Thornicroft, G., Baingana, F., Bolton, P., Chisholm, D., Collins, P. Y., Cooper, J. L., Eaton, J., Herrman, H., Herzallah, M. M., Huang, Y., Jordans, M. J. D., Kleinman, A., Medina-Mora, M. E., Morgan, E., Niaz, U., Omigbodun, O., … UnÜtzer, Jü. (2018). The Lancet Commission on global mental health and sustainable development. *The Lancet*, *392*(10157), 1553–1598. https://doi.org/10.1016/S0140-6736(18)31612-X

For peer review only - http://bmjopen.bmj.com/site/about/guidelines.xhtml

Pedersen, G. A., Smallegange, E., Coetzee, A., Hartog, K., Turner, J., Jordans, M. J. D., & Brown, F. L. (2019). A Systematic Review of the Evidence for Family and Parenting Interventions in Low- and Middle-Income Countries: Child and Youth Mental Health Outcomes. *Journal of Child and Family Studies*, *28*(8), 2036–2055. https://doi.org/10.1007/s10826-019-01399-4

Pfadenhauer, L. M., Gerhardus, A., Mozygemba, K., Lysdahl, K. B., Booth, A., Hofmann, B., Wahlster, P., Polus, S., Burns, J., Brereton, L., & Rehfuess, E. (2017). Making sense of complexity in context and implementation: The Context and Implementation of Complex Interventions (CICI) framework. *Implementation Science*, *12*(1), 1–17. https://doi.org/10.1186/s13012-017-0552-5

Pokhilenko, I., Janssen, L. M. M., Paulus, A. T. G., Drost, R. M. W. A., Hollingworth, W., Thorn, J. C., Noble, S., Simon, J., Fischer, C., Mayer, S., Salvador-Carulla, L., Konnopka, A., Hakkaart van Roijen, L., Brodszky, V., Park, A.-L., Evers, S. M. A. A., Berger, M., Łaszewska, A., Perić, N., … Thorn, J. (2023). Development of an Instrument for the Assessment of Health-Related Multi-sectoral Resource Use in Europe: The PECUNIA RUM. *Applied Health Economics and Health Policy*, *21*(2), 155–166. https://doi.org/10.1007/s40258-022-00780-7

Reichenheim, M., & Bastos, J. L. (2021). What, what for and how? Developing measurement instruments in epidemiology. *Rev Saude Publica*, *55*, 40. https://doi.org/10.11606/s1518-8787.2021055002813

Rice, F., Sellers, R., Hammerton, G., Eyre, O., Bevan-Jones, R., Thapar, A. K., Collishaw, S., Harold, G. T., & Thapar, A. (2017). Antecedents of new-onset major depressive disorder in children and adolescents at high familial risk. *JAMA Psychiatry*, *74*(2), 153–160. https://doi.org/10.1001/jamapsychiatry.2016.3140

Ross, D. A., Hinton, R., Melles-Brewer, M., Engel, D., Zeck, W., Fagan, L., Herat, J., Phaladi, G., Imbago-Jácome, D., Anyona, P., Sanchez, A., Damji, N., Terki, F., Baltag, V., Patton, G., Silverman, A., Fogstad, H., Banerjee, A., & Mohan, A. (2020). Adolescent Well-Being: A Definition and Conceptual Framework. *Journal of Adolescent Health*, *67*(4), 472–476. https://doi.org/10.1016/j.jadohealth.2020.06.042

Sebre, S., Sprugevica, I., Novotni, A., Bonevski, D., Pakalniskiene, V., Popescu, D., Turchina, T., Friedrich, W., & Lewis, O. (2004). Cross-cultural comparisons of child-reported emotional and physical abuse: Rates, risk factors and psychosocial symptoms. *Child Abuse and Neglect*, *28*(1), 113–127. https://doi.org/10.1016/j.chiabu.2003.06.004

Segrott, J., Gillespie, D., Lau, M., Holliday, J., Murphy, S., Foxcroft, D., Hood, K., Scourfield, J., Phillips, C., Roberts, Z., Rothwell, H., Hurlow, C., & Moore, L. (2022). Effectiveness of the Strengthening Families Programme in the UK at preventing substance misuse in 10–14 year-olds: a pragmatic randomised controlled trial. *BMJ Open*, *12*(2), e049647. https://doi.org/10.1136/bmjopen-2021-049647

Shenderovich, Y., Eisner, M., Cluver, L., Doubt, J., Berezin, M. K., Majokweni, S., & Murray, A. L. (2018). What affects attendance and engagement in a parenting program in South Africa? *Prevention Science*, *19*(7), 977–986. https://doi.org/10.1007/s11121-018-0941-2

Shenderovich, Y., Eisner, M., Cluver, L., Doubt, J., Berezin, M., Majokweni, S., & Murray, A. L. A. L. (2019). Delivering a Parenting Program in South Africa: The Impact of Implementation on Outcomes. *Journal of Child and Family Studies*, *28*(4). https://doi.org/10.1007/s10826-018-01319-y

Simon, J., Łaszewska, A., Leutner, E., Spiel, G., Churchman, D., & Mayer, S. (2018). Cultural and linguistic transferability of the multi-dimensional OxCAP-MH capability instrument for outcome measurement in mental health: the German language version. *BMC Psychiatry*, *18*(1), 173. https://doi.org/10.1186/s12888-018-1762-3

For peer review only - http://bmjopen.bmj.com/site/about/guidelines.xhtml

Skeen, S., Laurenzi, C. A., Gordon, S. L., Du Toit, S., Tomlinson, M., Dua, T., Fleischmann, A., Kohl, K., Ross, D., Servili, C., Brand, A. S., Dowdall, N., Lund, C., Van Der Westhuizen, C., Carvajal-Aguirre, L., De Carvalho, C. E., & Melendez-Torres, G. J. (2019). Adolescent mental health program components and behavior risk reduction: A Meta-analysis. *Pediatrics*, *144*(2). https://doi.org/10.1542/peds.2018-3488

Topp, C. W., Østergaard, S. D., Søndergaard, S., & Bech, P. (2015). The WHO-5 Well-Being Index: A Systematic Review of the Literature. *Psychotherapy and Psychosomatics*, *84*(3), 167–176. https://doi.org/10.1159/000376585

UNICEF. (2018). *Parenting adolescents. A Regional Study on Parenting Adolescents and Parenting Support Programmes in Belarus, Bulgaria, Georgia, Moldova, Montenegro and Romania*.

UNICEF. (2021). *Programming guidance for parenting of adolescents*. https://www.unicef.org/reports/parenting-adolescents

UNICEF and WHO. (2021). *Helping adolescents thrive toolkit: strategies to promote and protect adolescent mental health and reduce self-harm and other risk behaviours*.

Veenstra, R., & Laninga-Wijnen, L. (2022). Peer network studies and interventions in adolescence. *Current Opinion in Psychology*, *44*, 157–163. https://doi.org/10.1016/j.copsyc.2021.09.015

Vergunst, F., Jenkinson, C., Burns, T., Anand, P., Gray, A., Rugkåsa, J., & Simon, J. (2017). Psychometric validation of a multi-dimensional capability instrument for outcome measurement in mental health research (OxCAP-MH). *Health and Quality of Life Outcomes*, *15*(1), 250. https://doi.org/10.1186/s12955-017-0825-3

WHO. (1998). *Wellbeing Measures in Primary Health Care/The Depcare Project.*

WHO. (2013). *Survey of adverse childhood experiences in the former Yugolav Republic of Macedonia*. https://www.euro.who.int/en/countries/north-macedonia/publications/more-publications/publications-archive/survey-of-adverse-childhood-experiences-in-the-former-yugoslav-republic-of-macedonia

WHO. (2018). *Adverse childhood experiences and associated health-risk behaviours in university students from the Republic of Moldova*. https://www.euro.who.int/en/countries/republic-of-moldova/publications/adverse-childhood-experiences-and-associated-health-risk-behaviours-in-university-students-from-the-republic-of-moldova-2018

WHO. (2020). *Guidelines on mental health promotive and preventive interventions for adolescents*. 1–120. https://www.who.int/publications/i/item/9789240011854

WHO. (2022). *WHO guidelines on parenting interventions to prevent maltreatment and enhance parent–child relationships with children aged 0–17 years.* https://www.who.int/teams/social-determinants-of-health/violence-prevention/parenting-guidelines

Wille, N., Badia, X., Bonsel, G., Burström, K., Cavrini, G., Devlin, N., Egmar, A.-C., Greiner, W., Gusi, N., Herdman, M., Jelsma, J., Kind, P., Scalone, L., & Ravens-Sieberer, U. (2010). Development of the EQ-5D-Y: a child-friendly version of the EQ-5D. *Quality of Life Research*, *19*(6), 875–886. https://doi.org/10.1007/s11136-010-9648-y

World Bank. (2022). *DataBank world development indicators.* https://databank.worldbank.org/source/world-development-indicators

For peer review only

For peer review only - http://bmjopen.bmj.com/site/about/guidelines.xhtml

Funding
Funded by the European Union Horizon Europe and UK Research and Innovation. Views and opinions expressed are however those of the author(s) only and do not necessarily reflect those of the European Union or UK Research and Innovation (UKRI). Neither the European Union nor the granting authorities can be held responsible for them. This work was funded by the European Union Horizon Europe research and innovation programme under grant agreement number 101095528 and by the UKRI under the UK government's Horizon Europe funding guarantee, grant number 10050850. The funders were not and will not be involved in the design of the study, the collection, analysis and interpretation of data or the writing of the protocol.

Acknowledgements
We thank the FLOURISH research and implementation teams: facilitators, supervisors, data assessors, coordinators, as well as all adolescents, parents, staff and external experts taking part in the project. We are thankful for the support of the local organisations and stakeholders. We thank the DSMB & ethics advisers Laura Ghiron and Patty Leijten, Data Protection Officer Peter Schartner, Christian Kubb for the project website, Barbara Poecher for coordination and project management support, Joanna Lai at UNICEF, Sabine Rakotomalala at WHO, PLH development team – especially Jenny Doubt for her work on the programme adaptation as well as Jamie Lachman and Lucie Cluver. We thank Slavica Gajdadzis-Khezhevikj, Maite Alguacil Marí as study co-investigators. For research assistance, we thank Lara, Barg, Katharina Buss, Mackenzie Martin, Bethan Pell, Abbey Rowe, Eve Ruet, Swetha Sampathkumar and other researchers.
RE, GM & YS are supported by DECIPHer, which is funded by Welsh Government through Health and Care Research Wales. GM and YS are also funded by the Wolfson Centre for Young People's Mental Health. The Wolfson Centre for Young People's Mental Health has been established with support from the Wolfson Foundation.

Author statement
HMF and YS led the preparation of the grant proposal and the design of the study. NC, RE, HMF, NC, NH, GL, GM, MR, JS, BS and YS, are PIs on the study and contributed to the conception and implementation of the study, including data collection. AB, VB, SG-K, JM, AP, DW, FW are co-Is and contributed to the conception and implementation of the study, including data collection. NH has been leading the adaptation of the intervention programme, with support from team members, in particular VB, AB, SG-K and MR. MR and SG-K have designed and delivered training in the intervention programme. HMF, NH, JM, AP, YS, FW have designed and delivered training in data collection and safety monitoring. HMF, JM, AP, and FW are key contributors to the design of the adolescent and caregiver data collection, analysis, and management, and oversee the project data management and ethical processes. RE, GM,YS are key contributors to the design of the process evaluation and advisory group data collection, analysis, and management. JS and DW designed the health economic analysis, oversaw the relevant toolkit development, assessment, analysis and cultural adaptation, and collaborated in the intervention cost evaluation. HMF is the study Coordinator. All authors have revised the manuscript, have approved the final version of the manuscript to be published, and have agreed to be accountable for all aspects of the work.

Data sharing statement
The project will make some anonymized quantitative datasets available via an open-access repository following the FAIR (Findable, Accessible, Interoperable, Reusable) principles. The research team will ensure that results will be published in open access peer-reviewed

For peer review only - http://bmjopen.bmj.com/site/about/guidelines.xhtml

publications or are made available in other ways, such as through pre-prints. Contact heather.foran@aau.at for queries related to overall data management plan and access. The research team will ensure that results will be published in open access peer-reviewed publications or are made available in other ways, such as through pre-prints.

Competing interests (conflicts of interest)
VB, HMF, NH, SG-K, GL, JM, MR, AP, YS, FW report participating in other research studies involving the PLH programme as investigators and team members. NH reports personal fees from academic institutions offering continued education for psychotherapy education in Germany, outside the submitted work; and NH has attended trainings in Triple P and PCIT. YS was involved in the South African trial, which is the previous large-scale evaluation of the PLH for Parents and Teens. HMF, NH, GL, and MR were principal investigators on the previous Horizon European project, RISE, evaluating the PLH for Young Children in Moldova and North Macedonia. JS is developer of the OxCAP-MH and PECUNIA RUM instruments. DW has no competing interest to declare.

Patient and public involvement
Patients and/or the public were involved in the design, or conduct, or reporting, or dissemination plans of this research. Refer to the Methods section for further details on the work of the advisory groups.

For peer review only

Figure 1. Initial programme theory

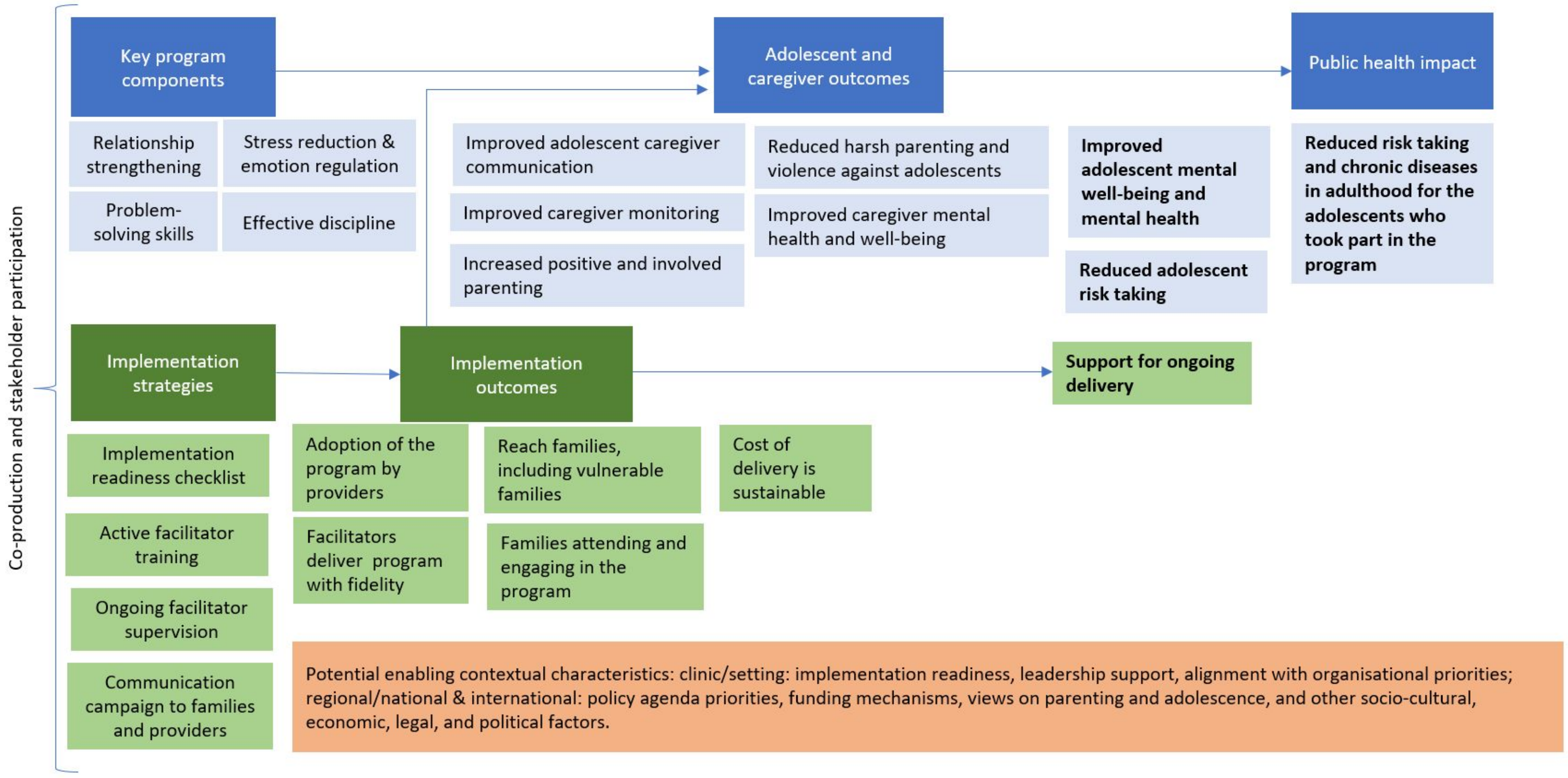

Supplementary materials

Table 1s Secondary outcome measures

Construct	Respondent	Measure
Communication	Adolescents & parents	Child-Parent Communication Apprehension scale, total score (Lucchetti et al., 2002)
		Family Assessment Device-FAD, general functioning subscale (Epstein et al., 1983)
Involved parenting	Adolescents & parents	Alabama Parenting Questionnaire (Frick, 1991), involved parenting and parental supervision/monitoring subscales.
Harsh parenting	Parents	Alabama Parenting Questionnaire (Frick, 1991), corporal punishment subscale.
Adolescent emotional problems	Adolescents	The Revised Child Anxiety and Depression Scales (RCADS), total score (Ebetsuani et al., 2012; Krause et al., 2021)
Adolescent behavior problems	Parents	Child Behavior Checklist 6-18, externalizing behavior (Achenbach & Rescorla, 2001)
Loneliness	Adolescents	UCLA-8 Loneliness scale, total score (Roberts et al., 1993)
	Parents	Revised UCLA-6 Loneliness scale, total score (Wongpakaran et al., 2020)
Social support	Adolescents & parents	Medical Outcome Study Social Support Survey, emotional and affectionate sub-scales (Sherbourne & Stewart, 1991).
Parents wellbeing	Parents	WHO-5 Well-Being Index, total score (WHO-5; Topp et al., 2015; WHO, 1998).
Parents psychological distress	Parents	The Patient Health Questionnaire – 9 (PHQ-9) , total score (Kroenke et al., 2001).
Parental stress	Parents	Parental Stress Scale, total score (Berry & Jones, 1995).

For peer review only

Table 2s. Other measures

Construct	Respondent	Measure
Enrolment	Facilitators	Percentage of participants who joined the study and have attended at least one group PLH session (individually for adolescents and caregivers, for both jointly at the dyad/family level)
Attendance	Facilitators	Percentage of group PLH sessions attended out of 6 (individually for adolescents and caregivers, for both jointly at the dyad/family level)<br>Percentage of catch-up PLH contact out of 6 (individually for adolescents and caregivers)
Adolescent healthy weight	Adolescents & parents	Body mass index (not expected to change but assessed for sample description).
Alcohol use	Parents	The Alcohol Use Disorder Identification Test (Saunders et al., 1993).
Posttraumatic stress	Parents	PTSD checklist for DSM-5 (PCL-5), short form, total score
	Adolescent	Children's Revised Impact of Event Scale (Perrin et al., 2005), total score and subscales
Sexual risk behaviors	Adolescents	Health Behavior in School-aged Children Study Sexual health scale, total score (Young et al., 2016).
Adolescent-defined problems	Adolescent	The Top Problem Assessment (Milgram et al., 2021; Weisz, 2012; Weisz et al., 2011).
Demographics	Adolescent & parents	Parent and adolescent age, gender, education, economic status.

For peer review only

For peer review only - http://bmjopen.bmj.com/site/about/guidelines.xhtml

REFERENCES

Achenbach, T. M., & Rescorla, L. (2001). *The Manual for the ASEBA School-Age Forms & Profiles.*

Berry, J. O., & Jones, W. H. (1995). The parental stress scale: Initial psychometric evidence. *Journal of Social and Personal Relationships*, *12*(3), 463–472. https://doi.org/10.1177/0265407595123009

Chorpita, B. F., Moffitt, C. E., & Gray, J. (2005). Psychometric properties of the Revised Child Anxiety and Depression Scale in a clinical sample. *Behaviour Research and Therapy*, *43*(3), 309–322. https://doi.org/10.1016/j.brat.2004.02.004

Epstein, N. B., Baldwin, L. M., & Bishop, D. S. (1983). The McMaster family assessment device. *Journal of Marital and Family Therapy*, *9*(2), 171–180. https://doi.org/10.1111/j.1752-0606.1983.tb01497.x

Frick, P. J. (1991). Alabama Parenting Questionnaire. *Unpublished Rating Scale, University of Alabama.*

Krause, K. R., Chung, S., Adewuya, A. O., Albano, A. M., Babins-Wagner, R., Birkinshaw, L., Brann, P., Creswell, C., Delaney, K., Falissard, B., Forrest, C. B., Hudson, J. L., Ishikawa, S., Khatwani, M., Kieling, C., Krause, J., Malik, K., Martínez, V., Mughal, F., … Wolpert, M. (2021). International consensus on a standard set of outcome measures for child and youth anxiety, depression, obsessive-compulsive disorder, and post-traumatic stress disorder. *The Lancet Psychiatry*, *8*(1), 76–86. https://doi.org/10.1016/S2215-0366(20)30356-4

Kroenke, K., Spitzer, R. L., & Williams, J. B. W. (2001). The PHQ-9: validity of a brief depression severity measure. *Journal of General Internal Medicine*, *16*(9), 606–613. https://doi.org/10.1046/j.1525-1497.2001.016009606.x

Lucchetti, A. E., Powers, W. G., & Love, D. E. (2002). The Empirical Development of the Child-Parent Communication Apprehension Scale for Use With Young Adults. *Journal of Family Communication*, *2*(3), 109–131. https://doi.org/10.1207/S15327698JFC0203_1

Milgram, L., Tonarely, N. A., & Ehrenreich-May, J. (2021). Youth top problems and early treatment response to the unified protocols for transdiagnostic treatment of emotional disorders in children and adolescents. *Child Psychiatry & Human Development*. https://doi.org/10.1007/s10578-021-01151-4

Perrin, S., Meiser-Stedman, R., & Smith, P. (2005). The Children's Revised Impact of Event Scale (CRIES): Validity as a Screening Instrument for PTSD. *Behavioural and Cognitive Psychotherapy*, *33*(4), 487–498. https://doi.org/10.1017/S1352465805002419

Roberts, R. E., Lewinsohn, P. M., & Seeley, J. R. (1993). A Brief Measure of Loneliness Suitable for Use with Adolescents. *Psychological Reports*, *72*(3_suppl), 1379–1391. https://doi.org/10.2466/pr0.1993.72.3c.1379

Russell, D. W. (1996). UCLA Loneliness Scale (Version 3): Reliability, Validity, and Factor Structure. *Journal of Personality Assessment*, *66*(1), 20–40. https://doi.org/10.1207/s15327752jpa6601_2

Saunders, J. B., Aasland, O. G., Babor, T. F., De la Fuente, J. R., & Grant, M. (1993).

For peer review only - http://bmjopen.bmj.com/site/about/guidelines.xhtml

Development of the Alcohol Use Disorders Identification Test (AUDIT): WHO Collaborative Project on Early Detection of Persons with Harmful Alcohol Consumption-II. *Addiction*, *88*(6), 791–804. https://doi.org/10.1111/j.1360-0443.1993.tb02093.x

Weisz, J. R. (2012). Testing standard and modular designs for psychotherapy treating depression, anxiety, and conduct problems in youth. *Archives of General Psychiatry*, *69*(3), 274. https://doi.org/10.1001/archgenpsychiatry.2011.147

Weisz, J. R., Chorpita, B. F., Frye, A., Ng, M. Y., Lau, N., Bearman, S. K., Ugueto, A. M., Langer, D. A., & Hoagwood, K. E. (2011). Youth top problems: Using idiographic, consumer-guided assessment to identify treatment needs and to track change during psychotherapy. *Journal of Consulting and Clinical Psychology*, *79*(3), 369–380. https://doi.org/10.1037/a0023307

Wongpakaran, N., Wongpakaran, T., Pinyopornpanish, M., Simcharoen, S., Suradom, C., Varnado, P., & Kuntawong, P. (2020). Development and validation of a 6-item Revised UCLA Loneliness Scale (RULS-6) using Rasch analysis. *British Journal of Health Psychology*, *25*(2), 233–256. https://doi.org/10.1111/bjhp.12404

Young, H., Költo, A., Reis, M., Saewyc, E. M., Moreau, N., Burke, L., Cosma, A., Windlin, B., Gabhainn, S. N., & Godeau, E. (2016). Sexual Health questions included in the Health Behaviour in School-aged Children (HBSC) Study: An international methodological pilot investigation. *BMC Medical Research Methodology*, *16*(1), 1–12. https://doi.org/10.1186/s12874-016-0270-8

For peer review only

For peer review only - http://bmjopen.bmj.com/site/about/guidelines.xhtml

STANDARD PROTOCOL ITEMS: RECOMMENDATIONS FOR INTERVENTIONAL TRIALS

SPIRIT 2013 Checklist: Recommended items to address in a clinical trial protocol and related documents*

Section/item	Item No	Description	Reported in the manuscript on page
**Administrative information**			
Title	1	Descriptive title identifying the study design, population, interventions, and, if applicable, trial acronym	1
Trial registration	2a	Trial identifier and registry name. If not yet registered, name of intended registry	3
	2b	All items from the World Health Organization Trial Registration Data Set	NA
Protocol version	3	Date and version identifier	NA
Funding	4	Sources and types of financial, material, and other support	19
Roles and responsibilities	5a	Names, affiliations, and roles of protocol contributors	1,19
	5b	Name and contact information for the trial sponsor	19
	5c	Role of study sponsor and funders, if any, in study design; collection, management, analysis, and interpretation of data; writing of the report; and the decision to submit the report for publication, including whether they will have ultimate authority over any of these activities	19
	5d	Composition, roles, and responsibilities of the coordinating centre, steering committee, endpoint adjudication committee, data management team, and other individuals or groups overseeing the trial, if applicable (see Item 21a for data monitoring committee)	NA
**Introduction**			
Background and rationale	6a	Description of research question and justification for undertaking the trial, including summary of relevant studies (published and unpublished) examining benefits and harms for each intervention	5,6

For peer review only

	6b	Explanation for choice of comparators	NA
Objectives	7	Specific objectives or hypotheses	6,7
Trial design	8	Description of trial design including type of trial (eg, parallel group, crossover, factorial, single group), allocation ratio, and framework (eg, superiority, equivalence, noninferiority, exploratory)	10,12
**Methods: Participants, interventions, and outcomes**			
Study setting	9	Description of study settings (eg, community clinic, academic hospital) and list of countries where data will be collected. Reference to where list of study sites can be obtained	6,7
Eligibility criteria	10	Inclusion and exclusion criteria for participants. If applicable, eligibility criteria for study centres and individuals who will perform the interventions (eg, surgeons, psychotherapists)	10,11
Interventions	11a	Interventions for each group with sufficient detail to allow replication, including how and when they will be administered	8,9
	11b	Criteria for discontinuing or modifying allocated interventions for a given trial participant (eg, drug dose change in response to harms, participant request, or improving/worsening disease)	13
	11c	Strategies to improve adherence to intervention protocols, and any procedures for monitoring adherence (eg, drug tablet return, laboratory tests)	8 (facilitator supervision)
	11d	Relevant concomitant care and interventions that are permitted or prohibited during the trial	NA
Outcomes	12	Primary, secondary, and other outcomes, including the specific measurement variable (eg, systolic blood pressure), analysis metric (eg, change from baseline, final value, time to event), method of aggregation (eg, median, proportion), and time point for each outcome. Explanation of the clinical relevance of chosen efficacy and harm outcomes is strongly recommended	11,12 and supplementary materials
Participant timeline	13	Time schedule of enrolment, interventions (including any run-ins and washouts), assessments, and visits for participants. A schematic diagram is highly recommended (see Figure)	8,9

For peer review only - http://bmjopen.bmj.com/site/about/guidelines.xhtml

Sample size	14	Estimated number of participants needed to achieve study objectives and how it was determined, including clinical and statistical assumptions supporting any sample size calculations	10
Recruitment	15	Strategies for achieving adequate participant enrolment to reach target sample size	10
**Methods: Assignment of interventions (for controlled trials)**			
Allocation:			
Sequence generation	16a	Method of generating the allocation sequence (eg, computer-generated random numbers), and list of any factors for stratification. To reduce predictability of a random sequence, details of any planned restriction (eg, blocking) should be provided in a separate document that is unavailable to those who enrol participants or assign interventions	NA
Allocation concealment mechanism	16b	Mechanism of implementing the allocation sequence (eg, central telephone; sequentially numbered, opaque, sealed envelopes), describing any steps to conceal the sequence until interventions are assigned	NA
Implementation	16c	Who will generate the allocation sequence, who will enrol participants, and who will assign participants to interventions	NA
Blinding (masking)	17a	Who will be blinded after assignment to interventions (eg, trial participants, care providers, outcome assessors, data analysts), and how	NA
	17b	If blinded, circumstances under which unblinding is permissible, and procedure for revealing a participant's allocated intervention during the trial	NA
**Methods: Data collection, management, and analysis**			
Data collection methods	18a	Plans for assessment and collection of outcome, baseline, and other trial data, including any related processes to promote data quality (eg, duplicate measurements, training of assessors) and a description of study instruments (eg, questionnaires, laboratory tests) along with their reliability and validity, if known. Reference to where data collection forms can be found, if not in the protocol	11,12

For peer review only - http://bmjopen.bmj.com/site/about/guidelines.xhtml

	18b	Plans to promote participant retention and complete follow-up, including list of any outcome data to be collected for participants who discontinue or deviate from intervention protocols	NA
Data management	19	Plans for data entry, coding, security, and storage, including any related processes to promote data quality (eg, double data entry; range checks for data values). Reference to where details of data management procedures can be found, if not in the protocol	11,12; 19, 20
Statistical methods	20a	Statistical methods for analysing primary and secondary outcomes. Reference to where other details of the statistical analysis plan can be found, if not in the protocol	11,12
	20b	Methods for any additional analyses (eg, subgroup and adjusted analyses)	11,12
	20c	Definition of analysis population relating to protocol non-adherence (eg, as randomised analysis), and any statistical methods to handle missing data (eg, multiple imputation)	NA
**Methods: Monitoring**			
Data monitoring	21a	Composition of data monitoring committee (DMC); summary of its role and reporting structure; statement of whether it is independent from the sponsor and competing interests; and reference to where further details about its charter can be found, if not in the protocol. Alternatively, an explanation of why a DMC is not needed	NA
	21b	Description of any interim analyses and stopping guidelines, including who will have access to these interim results and make the final decision to terminate the trial	NA
Harms	22	Plans for collecting, assessing, reporting, and managing solicited and spontaneously reported adverse events and other unintended effects of trial interventions or trial conduct	13
Auditing	23	Frequency and procedures for auditing trial conduct, if any, and whether the process will be independent from investigators and the sponsor	NA
**Ethics and dissemination**			

Research ethics approval	24	Plans for seeking research ethics committee/institutional review board (REC/IRB) approval	13
Protocol amendments	25	Plans for communicating important protocol modifications (eg, changes to eligibility criteria, outcomes, analyses) to relevant parties (eg, investigators, REC/IRBs, trial participants, trial registries, journals, regulators)	13
Consent or assent	26a	Who will obtain informed consent or assent from potential trial participants or authorised surrogates, and how (see Item 32)	10, 11, 13
	26b	Additional consent provisions for collection and use of participant data and biological specimens in ancillary studies, if applicable	NA
Confidentiality	27	How personal information about potential and enrolled participants will be collected, shared, and maintained in order to protect confidentiality before, during, and after the trial	13, 19, 20
Declaration of interests	28	Financial and other competing interests for principal investigators for the overall trial and each study site	20
Access to data	29	Statement of who will have access to the final trial dataset, and disclosure of contractual agreements that limit such access for investigators	19, 20
Ancillary and post-trial care	30	Provisions, if any, for ancillary and post-trial care, and for compensation to those who suffer harm from trial participation	NA
Dissemination policy	31a	Plans for investigators and sponsor to communicate trial results to participants, healthcare professionals, the public, and other relevant groups (eg, via publication, reporting in results databases, or other data sharing arrangements), including any publication restrictions	13, 20
	31b	Authorship eligibility guidelines and any intended use of professional writers	NA
	31c	Plans, if any, for granting public access to the full protocol, participant-level dataset, and statistical code	20

Appendices

For peer review only

For peer review only - http://bmjopen.bmj.com/site/about/guidelines.xhtml

Informed consent materials	32	Model consent form and other related documentation given to participants and authorised surrogates	NA We plan to post these on the study website
Biological specimens	33	Plans for collection, laboratory evaluation, and storage of biological specimens for genetic or molecular analysis in the current trial and for future use in ancillary studies, if applicable	NA

*It is strongly recommended that this checklist be read in conjunction with the SPIRIT 2013 Explanation & Elaboration for important clarification on the items. Amendments to the protocol should be tracked and dated. The SPIRIT checklist is copyrighted by the SPIRIT Group under the Creative Commons "Attribution-NonCommercial-NoDerivs 3.0 Unported" license.

For peer review only

BMJ Open

Family-focused intervention to promote adolescent mental health and well-being in Moldova and North Macedonia (FLOURISH): Feasibility study protocol

Journal:	*BMJ Open*
Manuscript ID	bmjopen-2023-080400.R1
Article Type:	Protocol
Date Submitted by the Author:	20-Oct-2023
Complete List of Authors:	Shenderovich, Yulia; Cardiff University, Centre for the Development, Evaluation, Complexity and Implementation in Public Health Improvement (DECIPHer), School of Social Sciences; Cardiff University, Wolfson Centre for Young People's Mental Health<br>Piolanti, Antonio; University of Klagenfurt, Institute of Psychology<br>Babii, Viorel; Asociatia Obsteasca Sanatate Pentru Tineri (Health for Youth Association)<br>Calovska-Hertzog, Nevena; Singidunum University, Department for Psychology, Faculty for Media and Communication; AST Centre for Education<br>Evans, Rhiannon; Cardiff University, Centre for the Development, Evaluation, Complexity and Implementation in Public Health Improvement (DECIPHer), School of Social Sciences<br>Heinrichs, Nina; Bielefeld University, Department of Psychology<br>Isakov, Anita Burgund; University of Belgrade, Faculty of Political Sciences, Department of Social Policy and Social Work<br>Lesco, Galina; Asociatia Obsteasca Sanatate Pentru Tineri (Health for Youth Association)<br>Moore, Graham; Cardiff University, Centre for the Development, Evaluation, Complexity and Implementation in Public Health Improvement (DECIPHer), School of Social Sciences; Cardiff University, Wolfson Centre for Young People's Mental Health<br>Mueller, Janina; University of Klagenfurt, Institute of Psychology<br>Raleva, Marija; Institute for Marriage, Family and Systemic Practice—ALTERNATIVA; Ss Cyril and Methodius University in Skopje, Department of Child and Adolescent Psychiatry<br>Shimbov, Bojan; Jaume I University Department of Economics, Instituto de Economía Internacional<br>Simon, Judith; Medical University of Vienna, Department of Health Economics, Center for Public Health; Oxford University, Department of Psychiatry, University of Oxford, Warneford Hospital<br>Waller, Franziska; University of Klagenfurt, Institute of Psychology<br>Wienand, Dennis; Medical University of Vienna, Department of Health Economics, Center for Public Health<br>Foran, Heather; University of Klagenfurt, Institute of Psychology
**Primary Subject Heading**:	Mental health

Secondary Subject Heading:	Global health, Mental health, Health services research, Public health
Keywords:	Adolescents < Adolescent, PUBLIC HEALTH, MENTAL HEALTH

SCHOLARONE™
Manuscripts

Family-focused intervention to promote adolescent mental health and well-being in Moldova and North Macedonia (FLOURISH): Feasibility study protocol

Yulia Shenderovich [1,2], Antonio Piolanti [3], Viorel Babii [4], Nevena Calovska-Hertzog [5,6], Rhiannon Evans [1], Nina Heinrichs [7], Anita Burgund Isakov [8], Galina Lesco [4], Graham Moore [1,2], Janina Mueller [3], Marija Raleva [9,10], Bojan Shimbov [11], Judit Simon [12,13], Franziska Waller [3], Dennis Wienand [12], Heather M. Foran [3]

1. Centre for the Development, Evaluation, Complexity and Implementation in Public Health Improvement (DECIPHer), School of Social Sciences, Cardiff University, Maindy Road, Cathays, Cardiff, CF24 4HQ, UK
2. Wolfson Centre for Young People's Mental Health, Cardiff University, Maindy Road, Cathays, Cardiff, CF24 4HQ, UK
3. Institute of Psychology, University of Klagenfurt, Universitaetstrasse 99, 9020 Klagenfurt am Woerthersee, Klagenfurt, Austria
4. Asociatia Obsteasca Sanatate Pentru Tineri (Health for Youth Association), Strada Socoleni 19, Chisinau 2020, Moldova
5. Department for Psychology, Faculty for Media and Communication, Singidunum University, Karadjordjeva 65, Belgrade, 11000, Serbia
6. AST Centre for Education, Djordja Kratovca 67, Belgrade, 11000, Serbia
7. Department of Psychology, Bielefeld University, Universitätsstrasse 25, 33615 Bielefeld, Germany
8. Faculty of Political Sciences, Department of Social Policy and Social Work, University of Belgrade, Jove Ilica 165, Belgrade, 11000, Serbia
9. Institute for Marriage, Family and Systemic Practice—ALTERNATIVA, Buld. Jane Sandanski 65, Skopje, 1000, North Macedonia
10. Department of Child and Adolescent Psychiatry, St. Cyril and Methodius University, Skopje, North Macedonia
11. Instituto de Economía Internacional, University Jaume I Castellon, Av. Vicent Sos Baynat, s/n 12071 Castellón de la Plana, Spain
12. Department of Health Economics, Center for Public Health, Medical University of Vienna, Kinderspitalgasse 15, 1090, Vienna, Austria
13. Department of Psychiatry, University of Oxford, Warneford Hospital, Oxford, OX3 7JX, UK

Yulia Shenderovich: ORCID 0000-0002-0254-3397; shenderovichy@cardiff.ac.uk;
Antonio Piolanti: ORCID 0000-0002-0613-0696; Antonio.Piolanti@aau.at
Viorel Babii: viorel.babii@gmail.com
Nevena Calovska-Hertzog: nevena.calovska@gmail.com
Rhiannon Evans: ORCID 0000-0002-0239-6331 ; EvansRE8@cardiff.ac.uk
Nina Heinrichs: ORCID 0000-0002-8301-5798 ; nina.heinrichs@uni-bielefeld.de
Anita Burgund Isakov: ORCID 0000-0002-4547-4934 ; anita.burgund@gmail.com
Galina Lesco: galina.lesco@gmail.com
Graham Moore: ORCID 0000-0002-6136-3978 ; MooreG@cardiff.ac.uk
Janina Mueller: ORCID 0000-0002-5005-2218 Janina.Mueller@aau.at
Marija Raleva: ORCID 0000-0002-5636-2612 ; marijaraleva@gmail.com
Bojan Shimbov: ORCID 0000-0002-0316-2430 ; shimbov@uji.es
Judit Simon : ORCID 0000-0001-9279-8627 ; judit.simon@meduniwien.ac.at
Franziska Waller: ORCID 0000-0002-4576-4325 ; Franziska.Waller@aau.at
Dennis Wienand : ORCID 0000-0002-5183-1352 ; dennis.wienand@meduniwien.ac.at

For peer review only - http://bmjopen.bmj.com/site/about/guidelines.xhtml

Heather M. Foran : ORCID 0000-0001-6659-5500; heather.foran@aau.at

Corresponding author: Yulia Shenderovich; address: spark, Maindy Road, Cathays, Cardiff, CF24 4HQ, UK; phone: +447774955977; email: shenderovichy@cardiff.ac.uk

Keywords: adolescents, parenting, mental health, adaptation, implementation science

For peer review only

ABSTRACT

Introduction

FLOURISH project will adapt, implement, and evaluate a programme to support adolescent mental health and well-being through strategies, such as strengthening parenting practices, adolescent-caregiver relationships, adolescent and parent socioemotional skills, and social support.

Methods and analysis

The project will focus on adolescents aged 10-14 and their caregivers in North Macedonia and Moldova. The countries were selected based on implementation readiness of two organisations and a need for accessible evidence-informed services to help mitigate health risks due to economic, social, and political challenges. Parenting for Lifelong Health (PLH) for Parents and Teens is a family-based programme developed for low-resource settings. PLH has been adapted with input from advisory groups. The programme includes additional components to strengthen impacts on adolescents: adolescent mental health tools, based on UNICEF's Helping Adolescents Thrive, adolescent peer support, and participation booster.

This pilot is first of three study phases. The pilot will be a feasibility testing of the adapted intervention and the assessment and implementation procedures to determine further refinements. The pilot will examine if the adapted programme is acceptable for adolescents, their families, and providers; explore contextual factors relevant to embedding this programme into longer-term scale-up; whether the programme can be delivered with fidelity and participation; whether the participants report changes in adolescent emotional and behaviour problems, well-being, and other outcomes; and whether the study tools are feasible and appropriate. Pre-post adolescent and caregiver questionnaires will provide outcome data. Process evaluation will include attendance and fidelity data, and focus groups. We will examine delivery cost and resource requirements.

Ethics and dissemination

The study was approved at the University of Klagenfurt (Austria), Medical Faculty at St. Cyril and Methodius University (North Macedonia), and National Committee of Ethical Expertise for Clinical Trials (Moldova). Through stakeholder engagement and dissemination, FLOURISH will advance scale-up of open-source family interventions.

Registration details

Trial registration: ID101095528; project page: https://www.flourish-study.org/about.html

For peer review only - http://bmjopen.bmj.com/site/about/guidelines.xhtml

ARTICLE SUMMARY

Strengths and limitations of this study

- This pilot study is embedded in a project designed to address a gap in evidence-informed programmes to promote adolescent mental health and well-being in North Macedonia and Moldova, at a time when migration, threat of war, and economic challenges exert particular pressures on adolescents and their families.
- The study is adapting an open-access intervention to new contexts, with a focus on scalability, to identify a model for adolescent-parent programmes that can be embedded in long-term delivery to provide a high-quality and accessible services in both countries.
- The pilot evaluation has a relatively small sample size and therefore cannot provide definitive information on the effects of the programme and its components; however, it will provide initial results on the feasibility of the intervention, its implementation and evaluation; effectiveness will be evaluated in further phases with a randomised design.

For peer review only

Family-focused intervention to promote adolescent mental health and well-being in Moldova and North Macedonia (FLOURISH): Feasibility study protocol

Word count: 4,014

INTRODUCTION

Adolescence is a period of transition and transformation, when one can acquire and strengthen factors contributing to lifelong health (1). Mental health problems during early adolescence are a global concern as this is when about half of mental health problems emerge (2). Stressful experiences in early adolescence are more strongly associated with a shorter total life span than such stressors in other phases of childhood, suggesting early adolescence is a sensitive period, making it a crucial time for providing additional support (3).

Having supportive relationships is associated with good health (4). The relationships adolescents have with their parents can remain influential, even as peers become increasingly important. Parents or caregivers are defined broadly to include any caregiver providing significant care to an adolescent, not limited to a biological parent (5). Parenting programmes focusing on the adolescent-caregiver relationships and parenting practices are recommended to support adolescent mental health (6–8). Furthermore, the WHO Guidelines on mental health promotive and preventive interventions for adolescents also recommend interventions for adolescents' interpersonal and emotional regulation skills (9).

A meta-analysis found no significant difference in effectiveness between transported and homegrown parenting interventions for reducing disruptive child behaviour (10). However, culturally adapted parenting interventions, both homegrown and transported, were more effective than non-adapted interventions (11). Adapting interventions can help maintain effectiveness while avoiding the cost of developing new interventions (12). Although majority of adolescents live in low- and middle-income countries (LMICs), research with adolescents in most LMICs is very limited (13), so evidence often has to be adapted from elsewhere.

The Family-Focused Adolescent & Lifelong Health Promotion (FLOURISH) project (2023-2026) is focused on adapting, optimising, and evaluating an intervention package for adolescents aged 10-14 and their caregivers in the Republic of North Macedonia and the Republic of Moldova (henceforth North Macedonia and Moldova). The intervention package aims to support adolescent mental health and well-being through building adolescents' and caregivers' skills, such as problem-solving and emotional regulation, strengthening social support, adolescent-caregiver relationships and communication, and improving parenting practices.

Moldova and North Macedonia were selected based on high risks for adolescent mental health, including adverse childhood experiences and poverty; lack of evidence-informed and accessible provision of prevention programmes for families with adolescents, paired with high levels of implementation readiness in two health networks. North Macedonia and Moldova are middle-income countries, among the poorest in Europe (14). Both have experienced rapid social, political, and economic transformation since 1990s following the fall of Communism.

Studies found high rates of physical punishment, emotional violence and neglect against children in both countries (15–17). Work migration often leads caregivers to spend time away from children, which can hamper communication (5). Young people also face limited work opportunities. Existing challenges were magnified by the coronavirus pandemic (18–20), and the war in Ukraine. There is stigma around help-seeking, particularly regarding mental health and well-being (21). A recent report found that in this region support services

For peer review only - http://bmjopen.bmj.com/site/about/guidelines.xhtml

for caregivers of adolescents, such as parenting workshops or support groups, are scarce, and highlighted the need for more such services (5), which our project aims to help address.

This protocol describes the adapted intervention, designed to promote adolescent mental health and well-being in Moldova and North Macedonia, and introduces the FLOURISH feasibility pilot (2023-24), which will inform the next phases of a larger study by providing information for further refinement of the intervention, and its implementation and evaluation procedures.

METHODS

Research questions and frameworks

FLOURISH is shaped by the Multiphase Optimization Strategy (MOST) framework. MOST is designed to optimise an intervention package within the key constraints in three phases – Preparation, Optimization, and Evaluation (22,23). This pilot study corresponds to the Preparation phase, focused on preparing and piloting the intervention and its evaluation. Following the pilot, a factorial trial will be conducted to select the most effective and efficient treatment package (Optimization phase). In the Evaluation phase, the revised programme package will be tested in an implementation-effectiveness randomised controlled trial.

Next, we discuss the research questions and additional frameworks and guidelines used in answering them (see Table 1 for an overview).

1. Is the adapted programme acceptable for adolescents, their caregivers, and staff in North Macedonia and Moldova, and what further adaptation are needed?

Programme materials have been adapted prior to piloting and will be revised throughout the project. FLOURISH draws on ADAPT adaptation guidance, which emphasises improving intervention fit with a new context while preserving key intervention functions (12,24).

2. What contextual factors may influence embedding of the programme into sustainable delivery and funding mechanisms in North Macedonia and Moldova?

FLOURISH is guided by the principle of “beginning with the end in mind” in ExpandNET/ World Health Organization (WHO) guidance (25). We will adapt the programme alongside developing a scaling-up strategy. This includes building capacity for scale-up and making choices to support institutionalization and expansion to more settings.

3. Can the adapted programme be delivered with high fidelity and family participation?

The pilot includes a process evaluation, guided by UK MRC process evaluation guidance (26), which focuses on implementation processes, potential mechanisms of impact, and contextual factors. To organise our understanding of the country context, we are using the Context and Implementation of Complex Interventions (CICI) framework (27).

4. What are the changes, if any, reported by the adolescents and caregivers on the primary outcomes of adolescent emotional problems, behaviour problems, and well-being, and on the secondary outcomes for adolescents and caregivers?

Primary programme targets are adolescent mental health and well-being. The pilot study will assess pre-post changes in the intervention outcomes reported by adolescents and their caregivers, following lessons learned in a previous pilot feasibility study, on which this project builds (28,29).

5. Are the study tools and their translations feasible and appropriate?

For peer review only - http://bmjopen.bmj.com/site/about/guidelines.xhtml

The pilot will examine the feasibility and preliminary psychometric performance of the outcome measures and their translations. A rigorous translation and evaluation process will be followed. The pilot will provide insights on the feasibility of the procedures and tools, and inform the next phase of the project, including which tools are retained, removed, or modified.

Table 1. Overview of the study design

Data	Participants	Research question	Framework
Advisory group consultations; focus groups	Adolescents, caregivers, staff, and (only for advisory groups) external professionals	1	ADAPT; process evaluation guidelines
Interviews	External professionals	2	CICI; ExpandNet
Attendance registers; observations; focus groups	Staff	3	Process evaluation guidelines
Questionnaires	Adolescents and caregivers	4	MOST
Think-aloud interviews; questionnaires	Adolescents and caregivers	5	N/A

Study sites

The study is conducted in two country-wide health networks, Institute for Marriage, Family and Systemic Practice (ALTERNATIVA) and Health for Youth Association. ALTERNATIVA is a network of psychologists, social workers, and family therapists in North Macedonia. Health for Youth Association in Moldova is an organisation which supports the activity of publicly funded youth-friendly clinics offering prevention and treatment in youth sexual and reproductive health, mental health, substance use, and violence prevention. Both organisations work with caregivers and adolescents, have delivered Parenting for Lifelong Health (PLH) programs, and participated in evaluations (28).

Study design

Intervention programme

The core programme examined in FLOURISH is PLH for Parents and Teens. It is a group behavioural programme (30,31), based on social learning theory (32). PLH was developed for LMICs in collaboration with UNICEF, WHO, and other international organisations and universities. Programme materials are focused on strengthening psychosocial skills and relationships, and are freely available online: https://www.who.int/teams/social-determinants-of-health/parenting-for-lifelong-health.

PLH programmes, primarily for caregivers with young children (aged 2-9), have been evaluated in multiple studies, including completed and ongoing trials in South Africa, Philippines, El Salvador, Lesotho, North Macedonia, Moldova, Romania, Thailand, Uganda, and Zambia. Evaluations reported high levels of engagement by families, practitioners, and other stakeholders. A pilot feasibility study and two large randomised trials in North Macedonia, Moldova, and Romania found improvements in parenting behaviours and child mental health (29,33), and further results are forthcoming (Foran et al.; Heinrichs et al.). PLH

For peer review only - http://bmjopen.bmj.com/site/about/guidelines.xhtml

for Parents and Teens (aged 10-17) has been evaluated in South Africa in two pre-post studies and an RCT (552 families, 40 clusters), with an embedded process evaluation (34,35). At 5-9 months after the 14-week intervention, caregivers in the intervention group, but not adolescents, reported lower rates of violence towards their adolescents and corporal punishment, and both caregivers and adolescents reported greater involved parenting and supervision of the adolescent by the caregiver (primary outcomes). There was no intervention effect detected on adolescent depression, suicidality, and externalising behaviour – however, these secondary outcomes were not the focus of the intervention.

Adaptation of the intervention

The adapted programme is drawing on the PLH manual used in the South African evaluation and on other programme versions. The initial adaptations have been informed by the input of advisory groups and expert interviews (described below), team's clinical expertise, and input from the programme developers. The adapted programme will be tested in the pilot (see the initial programme theory in Figure 1, to be revised throughout the project).

To support scalability, the programme was condensed into six group sessions, following on an introductory meeting with the caregiver and adolescent. As in the original model, sessions in FLOURISH will be delivered to a group of 10 adolescent-caregiver pairs. Each two-hour weekly session starts jointly, with a circle share, home activity discussion, agenda overview, physical exercises and games. Next, in the core lesson, a new skill is introduced and practiced, on topics developmentally relevant to adolescence. For the core lesson, the group is often split up into adolescent and caregiver parallel sessions to allow participants to share openly (see Table 2). After discussing the core lesson within the respective peer groups, participants come together and are encouraged to share the summary of their separate discussions in the joint group, and then further discuss lessons learned at home. Participants are given home practice tasks to do between the sessions. For participants who miss a session, facilitators offer a brief catch-up call or meeting. Facilitators and supervisors receive brief training in the programme and facilitation skills. Supervisors are trained in supervision. The programme is delivered by two facilitators per group, coached weekly by supervisors to promote quality of delivery. Given the focus on scalability, staff are not required to have a specific degree.

For peer review only - http://bmjopen.bmj.com/site/about/guidelines.xhtml

Table 2. *Adapted intervention structure using PLH material*

Week	Topic	Caregiver & adolescents
Pre-program	Individual meeting or call with facilitators to introduce the programme	Joint
1	Introducing the programme & ground rules. Psychoeducation about transitioning from a child to an adolescent: expectations, norms, and developmental stages	Separate
2	Relationship skills. Building a positive relationship through spending time together & praising each other	Joint
3	Talking about emotions and sensitive topics (sexuality, body changes, and intimate relationships)	Separate
4	Coping with difficult feelings (anger and stress)	Separate
5	Solving problems together as a family. How to communicate and solve problems around disagreements without conflict	Separate
6	Establishing rules and routines	Separate

To strengthen the impacts on adolescent outcomes, the adaptation of the programme involves supplementary components. Three components have been combined with the PLH programme into an intervention package: adolescent mental health tools, adolescent peer support, and adolescent participation booster.

Adolescent mental health tools. To strengthen adolescent mental health, we draw on the Helping Adolescents Thrive toolkit (HAT), designed to promote positive mental health, prevent mental health problems, and reduce engagement in self-harm and risk behaviours (36). This toolkit was developed based on a systematic review of universally delivered psychosocial interventions for adolescents (13), conducted to inform WHO Guidelines (9). The HAT materials will be delivered as six comics chapters, a workbook for adolescents and leaflets for caregivers, including, respectively, tips for adolescents to share their thoughts and feelings with their caregivers and questions caregivers can ask adolescents. The aim of these materials is to support adolescents and caregivers to communicate about adolescent mental health and well-being.

Adolescent peer support. Even brief interventions can foster friendships and social connections (37,38). Since parenting programmes have traditionally focused on parents, they have demonstrated positive impacts on social support experienced by parents but not by adolescents, including in the previous trial of PLH for Parents and Teens (30). The facilitators will pair up adolescents in the group for facilitating peer support and social connection. The PLH programme already includes peer pairings, but its potential effects have not yet been evaluated, so more emphasis will be placed on pairing up adolescents, and this will be tested as a separate component in the Optimization phase.

Adolescent participation booster. Although parenting programmes have been found acceptable in diverse settings, there is incomplete attendance (39–41). While many studies have explored parental attendance for parents of younger children (29), there is limited research on enhancing engagement of adolescents and their caregivers as programmes rarely included adolescents. The South African trial found 50-64% session attendance (42). We will investigate whether an adolescent participation booster promotes attendance.

Patient and public involvement

Members of the public were involved in the design of this research. In particular, intervention adaptations have been informed by advisory group consultations conducted in May-July 2023, as described below.

For peer review only - http://bmjopen.bmj.com/site/about/guidelines.xhtml

Advisory groups. Advisory groups of adolescents, caregivers, implementation organisation staff, and other professional experts (6-8 people per group) were formed in North Macedonia and Moldova to advise on programme adaptations and the study findings. Consultations will continue throughout the study, with key discussion points captured by a note-taker. Adolescents are eligible if they are aged 10-14 when joining the advisory group and provide adolescent assent and caregiver consent. For caregivers, staff, and other professionals, members need to be 18 or older and provide consent. For the caregiver advisory group, participants need to be caregivers of adolescents aged 10-17 (extended age range to facilitate recruitment). The groups aim to include individuals with experience of PLH or other family programmes and individuals who represent diverse perspectives (e.g., male and female caregivers, ethnic minorities). Implementation staff groups consist of ALTERNATIVA and Health for Youth Association staff. External professional experts include representatives from Ministries of Health, social services, youth, universities, research organisations, healthcare financing agencies, local NGOs, community, and international organisations implementing programmes for young people.

Advisory group analysis. Summary notes were combined into a matrix, using thematic framework and content analysis (43,44). We examined similarities and differences within and between stakeholder groups and countries. Advisory group participants received a summary for feedback as a means of validation.

Expert interviews. In addition, in each country, ten semi-structured audio-recorded interviews with professional experts took place to map the contextual facilitators, and barriers to scaling up a programme package prepared by the FLOURISH project. We drew on recommendations for qualitative sample sizes (45,46).

Interview analysis. We are using thematic and framework analysis for the transcripts. The CICI framework provides initial coding domains, with country-specific domains generated from the data. The analysis is conducted in two stages – first, a rapid synthesis to inform the intervention adaptations for the pilot (completed); second, a detailed analysis, generating a conceptual map of key contextual factors, presented as a narrative summary (ongoing).

Insert Figure 1 here

Figure 1. Initial programme theory

Piloting of the intervention

The pilot (October 2023-January 2024) will test the adapted intervention in a pre-post uncontrolled study with an outcome and process evaluation, described below.

Participants and recruitment. The pilot will include three intervention groups per country, so the target sample size is 60 adolescents and 60 caregivers (60 dyads). Recruitment will involve disseminating information through online and print materials, word-of-mouth, and via the existing clients of each network. At least one caregiver and adolescent per family will participate in the study. Inclusion criteria for caregivers are age 18 or older at baseline assessment; primary caregiver responsible for the care of an adolescent 10-14, resident in the same household at least four nights a week in the previous month; able to speak one of the local languages, in which the programme will be offered; agreement to participate in the programme; provision of consent for self and adolescent to participate in the study. Inclusion criteria for adolescents: age 10-14 at baseline assessment; assent to participate; caregiver consent.

The caregiver can be any adult who is primarily responsible for looking after the adolescent and available to participate. In cases where there is more than one caregiver who is available, they will need to agree who will be the primary and secondary participant.

For peer review only - http://bmjopen.bmj.com/site/about/guidelines.xhtml

Similarly, if two adolescents in the same age range in a family would like to join, both can join. Everyone who joins the study, both primary and secondary participants, will be invited to complete assessments.

Taking a proportionate universalism approach (47), all families will be eligible, but we will make greater efforts to include more vulnerable groups. We conceptualise our approach as selected prevention, targeted to individuals whose risk is above average, without screening (48). FLOURISH has no exclusion criteria. While introducing the project, the researchers will guide the participants through a consent form that asks whether they are currently experiencing acute distress, or a mental or physical health condition that would interfere with participation. The judgement will be made by the potential participants. If they decide they are not able to take part, the research team will follow up and provide referrals for other services (see also Ethics section).

To deliver the programme, facilitators and supervisors will be recruited from existing staff and networks of the implementing organisations. Inclusion criteria for facilitators are age 18 or older; participation in training; agreement to deliver the programme; provision of study consent. For supervisors: age 18 or older; participation in training; agreement to provide supervision; provision of consent.

Pre-post outcomes. Outcome data collection with families will be done primarily using computer-assisted self-interviewing questionnaires on tablets. Trained assessors will check participant eligibility as part of the informed consent procedure and assist adolescents and caregivers with completing the questionnaires. See Table 3 for the primary outcome measures and Table 1s (supplementary) for secondary outcome measures and Table 2s for other measures (pre-registered in clinical trials registry; ID101095528).

Outcome measures without available official translations will be adapted and translated following best practices (49). First, a cultural evaluation (relevance, appropriateness) of each item and forward-translation will be conducted by a professional with mental health experience. Second, a back-translation to English will be carried out by another translator. Third, an additional translator will compare the translations and assess correspondence between semantic equivalence and words, highlighting any discrepancies. A final translation will be produced in a meeting between the translators. All translators will be bilingual.

Think-aloud interviews will be conducted for the primary outcome measures that have not been previously used with adolescents or in the study countries (RCADS, WHO-5), and for the health economic measures that will be used in the Optimization and Evaluation phases – EQ-5D-5L (50), EQ-5D-Y (51), OxCAP-MH (52,53), PECUNIA RUM (54). Think-aloud interviews will be conducted after post-test assessments and explore item comprehension (10 interviews per country with adolescents and caregivers in Romanian and Macedonian).

Table 3. Primary Outcomes

Construct	Respondent	Measure
Adolescent emotional problems	Adolescent	- The Revised Child Anxiety and Depression Scales (RCADS), anxiety and depression subscales (55–57)<br>- Child Behaviour Checklist 6-18, internalising subscale (58)<br>- Youth Self-Report 11-18, internalising subscale (58)
Adolescent behaviour problems	Adolescent	Youth Self-Report, rule-breaking and aggressive behaviour
	Caregivers	Child Behaviour Checklist, rule-breaking and aggressive behaviour
Adolescent well-being	Adolescent	World Health Organization-Five Well-Being Index (WHO-5) (59,60)

For peer review only - http://bmjopen.bmj.com/site/about/guidelines.xhtml

Pre-post outcomes analysis. We will examine descriptive and preliminary psychometric statistics, such as internal consistencies. Intent-to-treat analyses will examine pre-post changes in adolescent and caregiver outcomes. We hypothesise that adolescent emotional problems and adolescent behaviour problems, both in self- and caregiver report, will be significantly reduced at the post-assessment; adolescent well-being will be significantly increased at the post-assessment, with medium to large effect sizes (Cohen's d => .5). We also will examine pre-post change in secondary outcomes and hypothesise small to medium effect sizes. Missing data will be addressed using full information maximum likelihood or multiple imputation methods.

Process evaluation. Facilitators will collect weekly attendance registers. Programme supervisors will conduct fidelity assessments. One session per facilitator will be observed live or video-recorded and assessed using the recording with the Facilitator Assessment Tool (61). Facilitators and supervisors will complete questionnaires to provide information on demographics and previous experience, and to collect pre-post data on staff well-being and parenting stress (for staff who are caregivers). The sample sizes are determined by the delivery model and the number of families in the intervention study.

Process evaluation analysis. We will use descriptive statistics, summarizing enrolment, attendance, fidelity, and facilitator characteristics. We will check variation in attendance by participant baseline characteristics (e.g., gender) and by intervention delivery characteristics (e.g., study site) to surface any emerging inequalities. We will explore whether attendance varied across sessions. We will explore staff responses to the pre-post well-being questionnaires using descriptives and paired t-tests. This will help inform, for example, whether additional support is needed if staff well-being had reduced.

Intervention costs. Facilitators and supervisors will complete questionnaires to capture the time and other resources spent preparing for, delivering, and following up on intervention activities. They will complete one form for the initial training and weekly forms designed to separate the resources required for the intervention versus the study.

Costs analysis. We will assess if weekly surveys are feasible. We will conduct descriptive analyses, summarizing information on time, money, and other resources used. Resource use information will be costed with country-specific unit costs collected primarily for the study to provide overall cost data. Cost information will be used to inform scale-up planning, and, in later phases, for programme cost-effectiveness analyses.

Focus groups. To inform further programme adaptations and answer process evaluation questions about the context, how the programme was implemented, and its mechanisms of change, we will conduct participant focus groups. A sub-sample of the adolescents, caregivers, and intervention staff will be recruited to participate in post-program audio-recorded focus groups (6-8 people/group, minimum 3 groups/country). We will aim to select participants with diverse backgrounds and experiences.

Focus group analysis. Focus group transcripts will be analysed using thematic and framework analysis, with codes developed both based on the initial questions and unexpected insights.

For peer review only

ETHICS AND DISSEMINATION

An ethical self-assessment for Horizon Europe was reviewed by the European Commission, and the project was cleared for ethics. The study was approved by the Institutional Review Board for Research Ethics at the University of Klagenfurt, Austria (coordinating site), the Ethical Commission for Human Research at the Medical Faculty at St. Cyril and Methodius University, North Macedonia, and National Committee of Ethical Expertise for Clinical Trials of the Ministry of Health, Moldova.

Participants will provide written assent (adolescents) or consent (adults). Adolescents and caregivers will receive reimbursements for transport costs, and thank-you vouchers for data collection activities, worth ~ €10. All staff will be trained to identify and handle potential distress in participants with detailed safety procedures and referral processes. Adverse event monitoring will help detect any potential harm caused by the research or the intervention. Any serious adverse events will be investigated and reported to the ethics committees and the data and safety monitoring board to consider potential changes in the project.

The FLOURISH project emphasises dissemination to stakeholders. Dissemination will include meetings, communication with media, and through print and online channels, including existing networks, FLOURISH website and social media. The focus of dissemination is Moldova and North Macedonia, and we will also be engaging stakeholders in the Eastern Europe region, working with family and adolescent health associations, NGOs, government, and international agencies.

EXPECTED RESULTS

This pilot will provide insights on the feasibility of the intervention, its implementation and evaluation, and thus inform the factorial trial planned as the next research phase, guiding which changes need to be made to the intervention, implementation, and evaluation procedures. More broadly, this project will provide insights on how best to adapt and scale up a programme targeting family relationships and adolescent mental health at a time when economic problems, interpersonal and intergroup conflict, the threat of a wider war, and other issues contribute to increased stress for both adolescents and adults. We will draw on a set of frameworks and global health best practices (62), such as using open-access interventions that involve stakeholders in all stages of intervention adaptation and evaluation.

For peer review only - http://bmjopen.bmj.com/site/about/guidelines.xhtml

REFERENCES

1. Ross DA, Hinton R, Melles-Brewer M, Engel D, Zeck W, Fagan L, et al. Adolescent Well-Being: A Definition and Conceptual Framework. Journal of Adolescent Health [Internet]. 2020;67(4):472–6. Available from: https://doi.org/10.1016/j.jadohealth.2020.06.042
2. Rice F, Sellers R, Hammerton G, Eyre O, Bevan-Jones R, Thapar AK, et al. Antecedents of new-onset major depressive disorder in children and adolescents at high familial risk. JAMA Psychiatry. 2017;74(2):153–60.
3. Falconi A, Gemmill A, Dahl RE, Catalano R. Adolescent experience predicts longevity: Evidence from historical epidemiology. Journal of Developmental Origins of Health and Disease. 2014;5(3):171–7.
4. Holt-Lunstad J. Why Social Relationships Are Important for Physical Health: A Systems Approach to Understanding and Modifying Risk and Protection. Annual Review of Psychology. 2018;69:437–58.
5. UNICEF. Parenting adolescents. A Regional Study on Parenting Adolescents and Parenting Support Programmes in Belarus, Bulgaria, Georgia, Moldova, Montenegro and Romania. 2018.
6. Pedersen GA, Smallegange E, Coetzee A, Hartog K, Turner J, Jordans MJD, et al. A Systematic Review of the Evidence for Family and Parenting Interventions in Low- and Middle-Income Countries: Child and Youth Mental Health Outcomes. Journal of Child and Family Studies. 2019;28(8):2036–55.
7. UNICEF. Programming guidance for parenting of adolescents [Internet]. 2021. Available from: https://www.unicef.org/reports/parenting-adolescents
8. Butchart A. WHO Guideline on parenting to prevent child maltreatment and promote positive development in children aged 0-17 years. 2020. 1–37 p.
9. WHO. Guidelines on mental health promotive and preventive interventions for adolescents. 2020;1–120. Available from: https://www.who.int/publications/i/item/9789240011854
10. Leijten P, Melendez-Torres GJ, Knerr W, Gardner F. Transported versus homegrown parenting interventions for reducing disruptive child behavior: A multilevel meta-regression study. Journal of the American Academy of Child and Adolescent Psychiatry [Internet]. 2016;55(7):610–7. Available from: http://dx.doi.org/10.1016/j.jaac.2016.05.003
11. Gardner F, Montgomery P, Knerr W. Transporting evidence-based parenting programs for child problem behavior (age 3–10) between countries: Systematic review and meta-analysis. Journal of Clinical Child & Adolescent Psychology [Internet]. 2015;45:749–62. Available from: http://dx.doi.org/10.1080/15374416.2015.1015134
12. Moore G, Campbell M, Copeland L, Craig P, Movsisyan A, Hoddinott P, et al. Adapting interventions to new contexts-the ADAPT guidance. The BMJ. 2021;374(fig 1).
13. Skeen S, Laurenzi CA, Gordon SL, Du Toit S, Tomlinson M, Dua T, et al. Adolescent mental health program components and behavior risk reduction: A Meta-analysis. Pediatrics. 2019;144(2).
14. World Bank. DataBank world development indicators. [Internet]. 2022. Available from: https://databank.worldbank.org/source/world-development-indicators
15. Sebre S, Sprugevica I, Novotni A, Bonevski D, Pakalniskiene V, Popescu D, et al. Cross-cultural comparisons of child-reported emotional and physical abuse: Rates, risk factors and psychosocial symptoms. Child Abuse and Neglect. 2004;28(1):113–27.
16. WHO. Survey of adverse childhood experiences in the former Yugolav Republic of

For peer review only

Macedonia [Internet]. 2013. Available from: https://www.euro.who.int/en/countries/north-macedonia/publications/more-publications/publications-archive/survey-of-adverse-childhood-experiences-in-the-former-yugoslav-republic-of-macedonia
17. WHO. Adverse childhood experiences and associated health-risk behaviours in university students from the Republic of Moldova [Internet]. 2018. Available from: https://www.euro.who.int/en/countries/republic-of-moldova/publications/adverse-childhood-experiences-and-associated-health-risk-behaviours-in-university-students-from-the-republic-of-moldova-2018
18. Bhatia A, Fabbri C, Cerna-Turoff I, Tanton C, Knight L, Turner E, et al. COVID-19 response measures and violence against children. Bulletin of the World Health Organization. 2020;98(9):583-583A.
19. Bhatia A, Fabbri C, Cerna-Turoff I, Turner E, Lokot M, Warria A, et al. Violence against children during the COVID-19 pandemic. Bulletin of the World Health Organization [Internet]. 2021 Oct 1;99(10):730–8. Available from: https://www.ncbi.nlm.nih.gov/pmc/articles/PMC8477433/pdf/BLT.20.283051.pdf
20. Katz I, Katz C, Andresen S, Bérubé A, Collin-Vezina D, Fallon B, et al. Child maltreatment reports and child protection service responses during COVID-19: Knowledge exchange among Australia, Brazil, Canada, Colombia, Germany, Israel, and South Africa. Child Abuse and Neglect [Internet]. 2021 Jun;116(August 2020):105078. Available from: https://linkinghub.elsevier.com/retrieve/pii/S0145213421001514
21. Dlouhy M. Mental health policy in Eastern Europe: A comparative analysis of seven mental health systems. BMC Health Services Research. 2014;14.
22. Collins LM. Optimization of Behavioral, Biobehavioral, and Biomedical Interventions [Internet]. Cham: Springer International Publishing; 2018. (Statistics for Social and Behavioral Sciences; vol. 22). Available from: http://link.springer.com/10.1007/978-3-319-72206-1
23. Guastaferro K, Collins LM. Optimization Methods and Implementation Science: An Opportunity for Behavioral and Biobehavioral Interventions. Implementation Research and Practice. 2021;2:263348952110543.
24. Evans RE, Moore G, Movsisyan A, Rehfuess E. How can we adapt complex population health interventions for new contexts? Progressing debates and research priorities. Journal of Epidemiology and Community Health. 2021;75(1):40–5.
25. Ghiron L, Ramirez-Ferrero E, Badiani R, Benevides R, Ntabona A, Fajans P, et al. Promoting Scale-Up Across a Global Project Platform: Lessons from the Evidence to Action Project. Global Implementation Research and Applications [Internet]. 2021;1(2):69–76. Available from: https://doi.org/10.1007/s43477-021-00013-4
26. Moore G, Audrey S, Barker M, Bond L, Bonell C, Hardeman W, et al. Process evaluation of complex interventions: Medical Research Council guidance. British Medical Journal [Internet]. 2015;350:h1258. Available from: http://www.bmj.com/cgi/doi/10.1136/bmj.h1258
27. Pfadenhauer LM, Gerhardus A, Mozygemba K, Lysdahl KB, Booth A, Hofmann B, et al. Making sense of complexity in context and implementation: The Context and Implementation of Complex Interventions (CICI) framework. Implementation Science. 2017;12(1):1–17.
28. Frantz I, Foran HM, Lachman JM, Jansen E, Hutchings J, Băban A, et al. Prevention of child mental health problems in Southeastern Europe: A multicentre sequential study to adapt, optimise and test the parenting programme "Parenting for Lifelong Health for Young Children", protocol for stage 1, the feasibility study. BMJ Open.

For peer review only - http://bmjopen.bmj.com/site/about/guidelines.xhtml

2019;9(1).
29. Jansen E, Frantz I, Hutchings J, Lachman J, Williams M, Taut D, et al. Preventing child mental health problems in southeastern Europe: Feasibility study (phase 1 of MOST framework). Family Process. 2021;(August 2020):1–18.
30. Cluver LD, Meinck F, Steinert JI, Shenderovich Y, Doubt J, Herrero Romero R, et al. Parenting for Lifelong Health: a pragmatic cluster randomised controlled trial of a non-commercialised parenting programme for adolescents and their families in South Africa. BMJ Global Health [Internet]. 2018 Jan;3(1):e000539. Available from: http://
31. Lachman JM, Martin M, Booij A, Tsoanyane S, Majenga N. Parenting for Lifelong Health for Teens: Facilitator Assessment Tool Manual. Cape Town; 2019.
32. Bandura A. Social learning theory. New York: General Learning Press; 1977.
33. Lachman J, Heinrichs N, Jansen E, Brühl A, Taut D, Fang X, et al. Preventing child mental health problems through parenting interventions in Southeastern Europe (RISE): Protocol for a multi-country cluster randomized factorial study. Contemporary Clinical Trials [Internet]. 2019;86:105855. Available from: https://doi.org/10.1016/j.cct.2019.105855
34. Cluver LD, Meinck F, Steinert JI, Shenderovich Y, Doubt J, Herrero Romero R, et al. Parenting for Lifelong Health: A pragmatic cluster randomised controlled trial of a non-commercialised parenting programme for adolescents and their families in South Africa. BMJ Global Health [Internet]. 2018 Jan;3(1):e000539. Available from: http://
35. Shenderovich Y, Eisner M, Cluver L, Doubt J, Berezin M, Majokweni S, et al. Delivering a Parenting Program in South Africa: The Impact of Implementation on Outcomes. Journal of Child and Family Studies [Internet]. 2019 Feb;28(4). Available from: https://doi.org/10.1007/s10826-018-01319-y
36. UNICEF. HIV and AIDS in adolescents [Internet]. 2021 [cited 2021 Jul 15]. Available from: https://data.unicef.org/topic/adolescents/hiv-aids/
37. Echols L, Ivanich J. From "Fast Friends" to True Friends: Can a Contact Intervention Promote Friendships in Middle School? Journal of Research on Adolescence. 2021;31(4):1152–71.
38. Veenstra R, Laninga-Wijnen L. Peer network studies and interventions in adolescence. Current Opinion in Psychology [Internet]. 2022;44:157–63. Available from: https://doi.org/10.1016/j.copsyc.2021.09.015
39. Butler AM, Titus C. Systematic Review of Engagement in Culturally Adapted Parent Training for Disruptive Behavior. Journal of Early Intervention. 2015;37(4):300–18.
40. Chacko A, Jensen SA, Lowry LS, Cornwell M, Chimklis A, Chan E, et al. Engagement in behavioral parent training: Review of the literature and implications for practice. Clinical Child and Family Psychology Review. 2016;19(3):204–15.
41. Gonzalez C, Morawska A, Haslam DM. Enhancing Initial Parental Engagement in Interventions for Parents of Young Children: A Systematic Review of Experimental Studies. Clinical Child and Family Psychology Review [Internet]. 2018;21(3):415–32. Available from: https://doi.org/10.1007/s10567-018-0259-4
42. Shenderovich Y, Eisner M, Cluver L, Doubt J, Berezin MK, Majokweni S, et al. What affects attendance and engagement in a parenting program in South Africa? Prevention Science. 2018;19(7):977–86.
43. Gale NK, Heath G, Cameron E, Rashid S, Redwood S. Using the framework method for the analysis of qualitative data in multi-disciplinary health research. BMC Medical Research Methodology [Internet]. 2013 Dec 18;13(1):117. Available from: https://bmcmedresmethodol.biomedcentral.com/articles/10.1186/1471-2288-13-117
44. Heath G, Cameron E, Cummins C, Greenfield S, Pattison H, Kelly D, et al. Paediatric "care closer to home": Stakeholder views and barriers to implementation. Health and

For peer review only - http://bmjopen.bmj.com/site/about/guidelines.xhtml

Place [Internet]. 2012;18(5):1068–73. Available from: http://dx.doi.org/10.1016/j.healthplace.2012.05.003
45. Hennink M, Kaiser BN. Sample sizes for saturation in qualitative research: A systematic review of empirical tests. Social Science and Medicine [Internet]. 2022;292:114523. Available from: https://doi.org/10.1016/j.socscimed.2021.114523
46. Krueger RA. Focus groups: A practical guide for applied research. Sage publications; 2014.
47. Segrott J, Gillespie D, Lau M, Holliday J, Murphy S, Foxcroft D, et al. Effectiveness of the Strengthening Families Programme in the UK at preventing substance misuse in 10–14 year-olds: a pragmatic randomised controlled trial. BMJ Open. 2022;12(2):e049647.
48. National Research Council and Institute of Medicine. Preventing Mental, Emotional, and Behavioral Disorders Among Young People [Internet]. Preventing Mental, Emotional, and Behavioral Disorders Among Young People: Progress and Possibilities. Washington, D.C.: National Academies Press; 2009. Available from: https://www.ncbi.nlm.nih.gov/books/NBK32789/
49. Reichenheim M, Bastos JL. What, what for and how? Developing measurement instruments in epidemiology. Rev Saude Publica [Internet]. 2021 Aug 9;55:40. Available from: https://www.revistas.usp.br/rsp/article/view/189335
50. Herdman M, Gudex C, Lloyd A, Janssen M, Kind P, Parkin D, et al. Development and preliminary testing of the new five-level version of EQ-5D (EQ-5D-5L). Quality of Life Research [Internet]. 2011 Dec 9;20(10):1727–36. Available from: http://link.springer.com/10.1007/s11136-011-9903-x
51. Wille N, Badia X, Bonsel G, Burström K, Cavrini G, Devlin N, et al. Development of the EQ-5D-Y: a child-friendly version of the EQ-5D. Quality of Life Research [Internet]. 2010 Aug 20;19(6):875–86. Available from: http://link.springer.com/10.1007/s11136-010-9648-y
52. Vergunst F, Jenkinson C, Burns T, Anand P, Gray A, Rugkåsa J, et al. Psychometric validation of a multi-dimensional capability instrument for outcome measurement in mental health research (OxCAP-MH). Health and Quality of Life Outcomes [Internet]. 2017 Dec 28;15(1):250. Available from: https://hqlo.biomedcentral.com/articles/10.1186/s12955-017-0825-3
53. Simon J, Łaszewska A, Leutner E, Spiel G, Churchman D, Mayer S. Cultural and linguistic transferability of the multi-dimensional OxCAP-MH capability instrument for outcome measurement in mental health: the German language version. BMC Psychiatry [Internet]. 2018 Dec 5;18(1):173. Available from: https://bmcpsychiatry.biomedcentral.com/articles/10.1186/s12888-018-1762-3
54. Pokhilenko I, Janssen LMM, Paulus ATG, Drost RMWA, Hollingworth W, Thorn JC, et al. Development of an Instrument for the Assessment of Health-Related Multi-sectoral Resource Use in Europe: The PECUNIA RUM. Applied Health Economics and Health Policy [Internet]. 2023 Mar 9;21(2):155–66. Available from: https://link.springer.com/10.1007/s40258-022-00780-7
55. Ebesutani C, Reise SP, Chorpita BF, Ale C, Regan J, Young J, et al. The Revised Child Anxiety and Depression Scale-Short Version: Scale reduction via exploratory bifactor modeling of the broad anxiety factor. Psychological Assessment [Internet]. 2012;24(4):833–45. Available from: http://doi.apa.org/getdoi.cfm?doi=10.1037/a0027283
56. Krause K, Midgley N, Edbrooke-Childs J, Wolpert M. A comprehensive mapping of outcomes following psychotherapy for adolescent depression: The perspectives of young people, their parents and therapists. European Child and Adolescent Psychiatry

For peer review only - http://bmjopen.bmj.com/site/about/guidelines.xhtml

[Internet]. 2021;30(11):1779–91. Available from: https://doi.org/10.1007/s00787-020-01648-8
57. Krause KR, Chung S, Adewuya AO, Albano AM, Babins-Wagner R, Birkinshaw L, et al. International consensus on a standard set of outcome measures for child and youth anxiety, depression, obsessive-compulsive disorder, and post-traumatic stress disorder. The Lancet Psychiatry [Internet]. 2021 Jan;8(1):76–86. Available from: https://linkinghub.elsevier.com/retrieve/pii/S2215036620303564
58. Achenbach TM, Rescorla L. The Manual for the ASEBA School-Age Forms & Profiles. Burlington; 2001.
59. Topp CW, Østergaard SD, Søndergaard S, Bech P. The WHO-5 Well-Being Index: A Systematic Review of the Literature. Psychotherapy and Psychosomatics [Internet]. 2015;84(3):167–76. Available from: https://www.karger.com/Article/FullText/376585
60. WHO. Wellbeing Measures in Primary Health Care/The Depcare Project. Copenhagen; 1998.
61. Martin M, Lachman JM, Murphy H, Gardner F, Foran H. The development, reliability, and validity of the Facilitator Assessment Tool: An implementation fidelity measure used in Parenting for Lifelong Health for Young Children. Child: Care, Health and Development. 2023;49(3):591–604.
62. Patel V, Saxena S, Lund C, Thornicroft G, Baingana F, Bolton P, et al. The Lancet Commission on global mental health and sustainable development. The Lancet. 2018;392(10157):1553–98.

For peer review only

For peer review only - http://bmjopen.bmj.com/site/about/guidelines.xhtml

Funding
Funded by the European Union Horizon Europe and UK Research and Innovation. Views and opinions expressed are however those of the author(s) only and do not necessarily reflect those of the European Union or UK Research and Innovation (UKRI). Neither the European Union nor the granting authorities can be held responsible for them. This work was funded by the European Union Horizon Europe research and innovation programme under grant agreement number 101095528 and by the UKRI under the UK government's Horizon Europe funding guarantee, grant number 10050850. The funders were not and will not be involved in the design of the study, the collection, analysis and interpretation of data or the writing of the protocol.

Acknowledgements
We thank the FLOURISH research and implementation teams: facilitators, supervisors, data assessors, coordinators, as well as all adolescents, parents, staff and external experts taking part in the project. We are thankful for the support of the local organisations and stakeholders. We thank the DSMB & ethics advisers Laura Ghiron and Patty Leijten, Data Protection Officer Peter Schartner, Christian Kubb for the project website, Barbara Poecher for coordination and project management support, Joanna Lai at UNICEF, Sabine Rakotomalala at WHO, PLH development team – especially Jenny Doubt for her work on the programme adaptation as well as Jamie Lachman and Lucie Cluver. We thank Slavica Gajdadzis-Khezhevikj, Maite Alguacil Marí as study co-investigators. For research assistance, we thank Lara, Barg, Katharina Buss, Mackenzie Martin, Bethan Pell, Abbey Rowe, Eve Ruet, Swetha Sampathkumar and other researchers.
RE, GM & YS are supported by DECIPHer, which is funded by Welsh Government through Health and Care Research Wales. GM and YS are also funded by the Wolfson Centre for Young People's Mental Health. The Wolfson Centre for Young People's Mental Health has been established with support from the Wolfson Foundation.
For the purpose of open access, the authors have applied a CC BY public copyright licence to any Author Accepted Manuscript version arising.

Author statement
HMF and YS led the preparation of the grant proposal and the design of the study. NC, RE, HMF, NC, NH, GL, GM, MR, JS, BS and YS, are PIs on the study and contributed to the conception and implementation of the study, including data collection. AB, VB, SG-K, JM, AP, DW, FW are co-Is and contributed to the conception and implementation of the study, including data collection. NH has been leading the adaptation of the intervention programme, with support from team members, in particular VB, AB, SG-K and MR. MR and SG-K have designed and delivered training in the intervention programme. HMF, NH, JM, AP, YS, FW have designed and delivered training in data collection and safety monitoring. HMF, JM, AP, and FW are key contributors to the design of the adolescent and caregiver data collection, analysis, and management, and oversee the project data management and ethical processes. RE, GM,YS are key contributors to the design of the process evaluation and advisory group data collection, analysis, and management. JS and DW designed the health economic analysis, oversaw the relevant toolkit development, assessment, analysis and cultural adaptation, and collaborated in the intervention cost evaluation. HMF is the study Coordinator. All authors have revised the manuscript, have approved the final version of the manuscript to be published, and have agreed to be accountable for all aspects of the work.

Data sharing statement

For peer review only - http://bmjopen.bmj.com/site/about/guidelines.xhtml

The project will make some anonymized quantitative datasets available via an open-access repository following the FAIR (Findable, Accessible, Interoperable, Reusable) principles. The research team will ensure that results will be published in open access peer-reviewed publications or are made available in other ways, such as through pre-prints. Contact heather.foran@aau.at for queries related to overall data management plan and access. The research team will ensure that results will be published in open access peer-reviewed publications or are made available in other ways, such as through pre-prints.

Competing interests (conflicts of interest)

VB, HMF, NH, SG-K, GL, JM, MR, AP, YS, FW report participating in other research studies involving the PLH programme as investigators and team members. NH reports personal fees from academic institutions offering continued education for psychotherapy education in Germany, outside the submitted work; and NH has attended trainings in Triple P and PCIT. YS was involved in the South African trial, which is the previous large-scale evaluation of the PLH for Parents and Teens. HMF, NH, GL, and MR were principal investigators on the previous Horizon European project, RISE, evaluating the PLH for Young Children in Moldova and North Macedonia. JS is developer of the OxCAP-MH and PECUNIA RUM instruments. DW has no competing interest to declare.

For peer review only

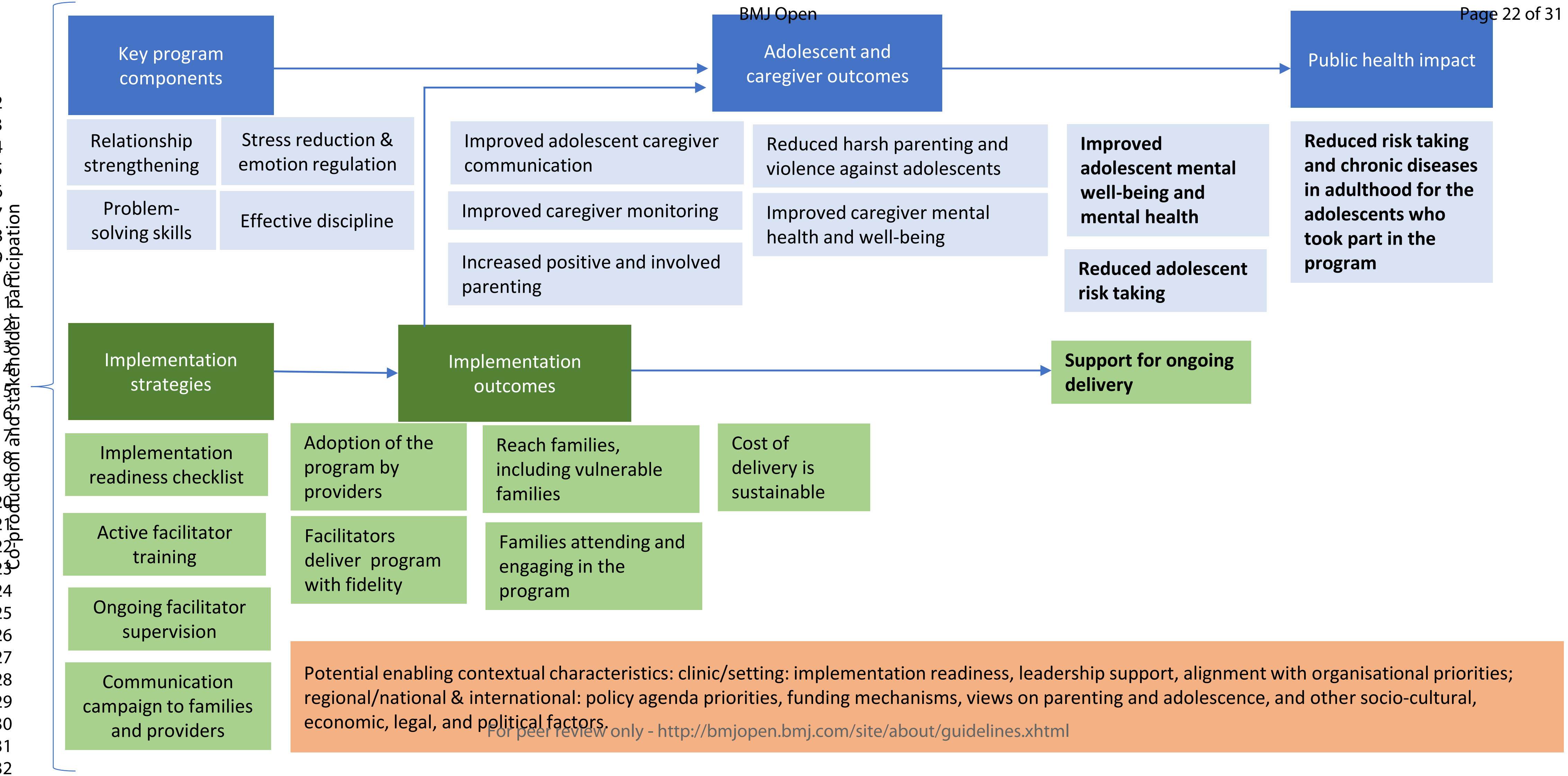
Co-production and stakeholder participation
Key program components
Adolescent and caregiver outcomes
Public health impact
Relationship strengthening
Stress reduction & emotion regulation
Problem-solving skills
Effective discipline
Improved adolescent caregiver communication
Improved caregiver monitoring
Increased positive and involved parenting
Reduced harsh parenting and violence against adolescents
Improved caregiver mental health and well-being
Improved adolescent mental well-being and mental health
Reduced adolescent risk taking
Reduced risk taking and chronic diseases in adulthood for the adolescents who took part in the program
Implementation strategies
Implementation outcomes
Support for ongoing delivery
Implementation readiness checklist
Active facilitator training
Ongoing facilitator supervision
Communication campaign to families and providers
Adoption of the program by providers
Facilitators deliver program with fidelity
Reach families, including vulnerable families
Families attending and engaging in the program
Cost of delivery is sustainable
Potential enabling contextual characteristics: clinic/setting: implementation readiness, leadership support, alignment with organisational priorities; regional/national & international: policy agenda priorities, funding mechanisms, views on parenting and adolescence, and other socio-cultural, economic, legal, and political factors.

Supplementary materials

Table 1s Secondary outcome measures

Construct	Respondent	Measure
Communication	Adolescents & parents	Child-Parent Communication Apprehension scale, total score (1)
		Family Assessment Device-FAD, general functioning subscale (2)
Involved parenting	Adolescents & parents	Alabama Parenting Questionnaire (3), involved parenting and parental supervision/monitoring subscales.
Harsh parenting	Parents	Alabama Parenting Questionnaire (3), corporal punishment subscale.
Adolescent emotional problems	Adolescents	The Revised Child Anxiety and Depression Scales (RCADS), total score (4–6)
Adolescent behavior problems	Parents	Child Behavior Checklist 6-18, externalizing behavior (7)
Loneliness	Adolescents	UCLA-8 Loneliness scale, total score (8)
	Parents	Revised UCLA-6 Loneliness scale, total score (9)
Social support	Adolescents & parents	Medical Outcome Study Social Support Survey, emotional and affectionate sub-scales (10)
Parents wellbeing	Parents	WHO-5 Well-Being Index, total score (WHO-5) (11,12)
Parents psychological distress	Parents	The Patient Health Questionnaire – 9 (PHQ-9) , total score (13).
Parental stress	Parents	Parental Stress Scale, total score (14).

For peer review only

Table 2s. Other measures

Construct	Respondent	Measure
Enrolment	Facilitators	Percentage of participants who joined the study and have attended at least one group PLH session (individually for adolescents and caregivers, for both jointly at the dyad/family level)
Attendance	Facilitators	Percentage of group PLH sessions attended out of 6 (individually for adolescents and caregivers, for both jointly at the dyad/family level)<br>Percentage of catch-up PLH contact out of 6 (individually for adolescents and caregivers)
Adolescent healthy weight	Adolescents & parents	Body mass index (not expected to change but assessed for sample description).
Alcohol use	Parents	The Alcohol Use Disorder Identification Test (15).
Posttraumatic stress	Parents	PTSD checklist for DSM-5 (PCL-5), short form, total score
	Adolescent	Children's Revised Impact of Event Scale (16), total score and subscales
Sexual risk behaviors	Adolescents	Health Behavior in School-aged Children Study Sexual health scale, total score (17).
Adolescent-defined problems	Adolescent	The Top Problem Assessment (18–20).
Demographics	Adolescent & parents	Parent and adolescent age, gender, education, economic status.

For peer review only

For peer review only - http://bmjopen.bmj.com/site/about/guidelines.xhtml

REFERENCES

1. Lucchetti AE, Powers WG, Love DE. The Empirical Development of the Child-Parent Communication Apprehension Scale for Use With Young Adults. Journal of Family Communication [Internet]. 2002 Jul;2(3):109–31. Available from: http://www.tandfonline.com/doi/abs/10.1207/S15327698JFC0203_1

2. Epstein NB, Baldwin LM, Bishop DS. The McMaster family assessment device. Journal of Marital and Family Therapy [Internet]. 1983 Apr;9(2):171–80. Available from: https://onlinelibrary.wiley.com/doi/10.1111/j.1752-0606.1983.tb01497.x

3. Frick PJ. Alabama Parenting Questionnaire. Unpublished rating scale, University of Alabama. 1991;

4. Chorpita BF, Moffitt CE, Gray J. Psychometric properties of the Revised Child Anxiety and Depression Scale in a clinical sample. Behaviour Research and Therapy [Internet]. 2005 Mar;43(3):309–22. Available from: https://linkinghub.elsevier.com/retrieve/pii/S0005796704000695

5. Krause K, Midgley N, Edbrooke-Childs J, Wolpert M. A comprehensive mapping of outcomes following psychotherapy for adolescent depression: The perspectives of young people, their parents and therapists. European Child and Adolescent Psychiatry [Internet]. 2021;30(11):1779–91. Available from: https://doi.org/10.1007/s00787-020-01648-8

6. Krause KR, Chung S, Adewuya AO, Albano AM, Babins-Wagner R, Birkinshaw L, et al. International consensus on a standard set of outcome measures for child and youth anxiety, depression, obsessive-compulsive disorder, and post-traumatic stress disorder. The Lancet Psychiatry [Internet]. 2021 Jan;8(1):76–86. Available from: https://linkinghub.elsevier.com/retrieve/pii/S2215036620303564

7. Achenbach TM, Rescorla L. The Manual for the ASEBA School-Age Forms & Profiles. Burlington; 2001.

8. Roberts RE, Lewinsohn PM, Seeley JR. A Brief Measure of Loneliness Suitable for Use with Adolescents. Psychological Reports [Internet]. 1993 Jun;72(3_suppl):1379–91. Available from: http://journals.sagepub.com/doi/10.2466/pr0.1993.72.3c.1379

9. Wongpakaran N, Wongpakaran T, Pinyopornpanish M, Simcharoen S, Suradom C, Varnado P, et al. Development and validation of a 6-item Revised UCLA Loneliness Scale (RULS-6) using Rasch analysis. British Journal of Health Psychology [Internet]. 2020 May 30;25(2):233–56. Available from: https://onlinelibrary.wiley.com/doi/10.1111/bjhp.12404

10. Sherbourne CD, Stewart AL. The MOS social support survey. Social Science and Medicine. 1991;32(6):705–14.

11. Topp CW, Østergaard SD, Søndergaard S, Bech P. The WHO-5 Well-Being Index: A Systematic Review of the Literature. Psychotherapy and Psychosomatics [Internet]. 2015;84(3):167–76. Available from: https://www.karger.com/Article/FullText/376585

12. WHO. Wellbeing Measures in Primary Health Care/The Depcare Project. Copenhagen; 1998.

13. Kroenke K, Spitzer RL, Williams JBW. The PHQ-9: validity of a brief depression

For peer review only - http://bmjopen.bmj.com/site/about/guidelines.xhtml

severity measure. Journal of General Internal Medicine [Internet]. 2001 Sep;16(9):606–13. Available from: http://link.springer.com/10.1046/j.1525-1497.2001.016009606.x

14. Berry JO, Jones WH. The parental stress scale: Initial psychometric evidence. Journal of Social and Personal Relationships. 1995;12(3):463–72.

15. Saunders JB, Aasland OG, Babor TF, De la Fuente JR, Grant M. Development of the Alcohol Use Disorders Identification Test (AUDIT): WHO Collaborative Project on Early Detection of Persons with Harmful Alcohol Consumption-II. Addiction. 1993;88(6):791–804.

16. Perrin S, Meiser-Stedman R, Smith P. The Children's Revised Impact of Event Scale (CRIES): Validity as a Screening Instrument for PTSD. Behavioural and Cognitive Psychotherapy [Internet]. 2005 Oct 14;33(4):487–98. Available from: https://www.cambridge.org/core/product/identifier/S1352465805002419/type/journal_article

17. Young H, Költo A, Reis M, Saewyc EM, Moreau N, Burke L, et al. Sexual Health questions included in the Health Behaviour in School-aged Children (HBSC) Study: An international methodological pilot investigation. BMC Medical Research Methodology. 2016;16(1):1–12.

18. Milgram L, Tonarely NA, Ehrenreich-May J. Youth top problems and early treatment response to the unified protocols for transdiagnostic treatment of emotional disorders in children and adolescents. Child Psychiatry & Human Development [Internet]. 2021 Mar 17; Available from: http://link.springer.com/10.1007/s10578-021-01151-4

19. Weisz JR, Chorpita BF, Frye A, Ng MY, Lau N, Bearman SK, et al. Youth top problems: Using idiographic, consumer-guided assessment to identify treatment needs and to track change during psychotherapy. Journal of Consulting and Clinical Psychology. 2011;79(3):369–80.

20. Weisz JR. Testing standard and modular designs for psychotherapy treating depression, anxiety, and conduct problems in youth. Archives of General Psychiatry [Internet]. 2012 Mar 1;69(3):274. Available from: http://archpsyc.jamanetwork.com/article.aspx?doi=10.1001/archgenpsychiatry.2011.147

For peer review only

SPIRIT

STANDARD PROTOCOL ITEMS: RECOMMENDATIONS FOR INTERVENTIONAL TRIALS

SPIRIT 2013 Checklist: Recommended items to address in a clinical trial protocol and related documents*

**Section/item**	**Item No**	**Description**	**Reported in the manuscript on page**
**Administrative information**			
Title	1	Descriptive title identifying the study design, population, interventions, and, if applicable, trial acronym	1
Trial registration	2a	Trial identifier and registry name. If not yet registered, name of intended registry	3
	2b	All items from the World Health Organization Trial Registration Data Set	NA
Protocol version	3	Date and version identifier	NA
Funding	4	Sources and types of financial, material, and other support	19
Roles and responsibilities	5a	Names, affiliations, and roles of protocol contributors	1,19
	5b	Name and contact information for the trial sponsor	19
	5c	Role of study sponsor and funders, if any, in study design; collection, management, analysis, and interpretation of data; writing of the report; and the decision to submit the report for publication, including whether they will have ultimate authority over any of these activities	19
	5d	Composition, roles, and responsibilities of the coordinating centre, steering committee, endpoint adjudication committee, data management team, and other individuals or groups overseeing the trial, if applicable (see Item 21a for data monitoring committee)	NA
**Introduction**			
Background and rationale	6a	Description of research question and justification for undertaking the trial, including summary of relevant studies (published and unpublished) examining benefits and harms for each intervention	5,6

For peer review only

	6b	Explanation for choice of comparators	NA
Objectives	7	Specific objectives or hypotheses	6,7
Trial design	8	Description of trial design including type of trial (eg, parallel group, crossover, factorial, single group), allocation ratio, and framework (eg, superiority, equivalence, noninferiority, exploratory)	10,12
**Methods: Participants, interventions, and outcomes**			
Study setting	9	Description of study settings (eg, community clinic, academic hospital) and list of countries where data will be collected. Reference to where list of study sites can be obtained	6,7
Eligibility criteria	10	Inclusion and exclusion criteria for participants. If applicable, eligibility criteria for study centres and individuals who will perform the interventions (eg, surgeons, psychotherapists)	10,11
Interventions	11a	Interventions for each group with sufficient detail to allow replication, including how and when they will be administered	8,9
	11b	Criteria for discontinuing or modifying allocated interventions for a given trial participant (eg, drug dose change in response to harms, participant request, or improving/worsening disease)	13
	11c	Strategies to improve adherence to intervention protocols, and any procedures for monitoring adherence (eg, drug tablet return, laboratory tests)	8 (facilitator supervision)
	11d	Relevant concomitant care and interventions that are permitted or prohibited during the trial	NA
Outcomes	12	Primary, secondary, and other outcomes, including the specific measurement variable (eg, systolic blood pressure), analysis metric (eg, change from baseline, final value, time to event), method of aggregation (eg, median, proportion), and time point for each outcome. Explanation of the clinical relevance of chosen efficacy and harm outcomes is strongly recommended	11,12 and supplementary materials
Participant timeline	13	Time schedule of enrolment, interventions (including any run-ins and washouts), assessments, and visits for participants. A schematic diagram is highly recommended (see Figure)	8,9

For peer review only - http://bmjopen.bmj.com/site/about/guidelines.xhtml

Sample size	14	Estimated number of participants needed to achieve study objectives and how it was determined, including clinical and statistical assumptions supporting any sample size calculations	10
Recruitment	15	Strategies for achieving adequate participant enrolment to reach target sample size	10
**Methods: Assignment of interventions (for controlled trials)**			
Allocation:			
Sequence generation	16a	Method of generating the allocation sequence (eg, computer-generated random numbers), and list of any factors for stratification. To reduce predictability of a random sequence, details of any planned restriction (eg, blocking) should be provided in a separate document that is unavailable to those who enrol participants or assign interventions	NA
Allocation concealment mechanism	16b	Mechanism of implementing the allocation sequence (eg, central telephone; sequentially numbered, opaque, sealed envelopes), describing any steps to conceal the sequence until interventions are assigned	NA
Implementation	16c	Who will generate the allocation sequence, who will enrol participants, and who will assign participants to interventions	NA
Blinding (masking)	17a	Who will be blinded after assignment to interventions (eg, trial participants, care providers, outcome assessors, data analysts), and how	NA
	17b	If blinded, circumstances under which unblinding is permissible, and procedure for revealing a participant's allocated intervention during the trial	NA
**Methods: Data collection, management, and analysis**			
Data collection methods	18a	Plans for assessment and collection of outcome, baseline, and other trial data, including any related processes to promote data quality (eg, duplicate measurements, training of assessors) and a description of study instruments (eg, questionnaires, laboratory tests) along with their reliability and validity, if known. Reference to where data collection forms can be found, if not in the protocol	11,12

	18b	Plans to promote participant retention and complete follow-up, including list of any outcome data to be collected for participants who discontinue or deviate from intervention protocols	NA
Data management	19	Plans for data entry, coding, security, and storage, including any related processes to promote data quality (eg, double data entry; range checks for data values). Reference to where details of data management procedures can be found, if not in the protocol	11,12; 19, 20
Statistical methods	20a	Statistical methods for analysing primary and secondary outcomes. Reference to where other details of the statistical analysis plan can be found, if not in the protocol	11,12
	20b	Methods for any additional analyses (eg, subgroup and adjusted analyses)	11,12
	20c	Definition of analysis population relating to protocol non-adherence (eg, as randomised analysis), and any statistical methods to handle missing data (eg, multiple imputation)	NA
**Methods: Monitoring**			
Data monitoring	21a	Composition of data monitoring committee (DMC); summary of its role and reporting structure; statement of whether it is independent from the sponsor and competing interests; and reference to where further details about its charter can be found, if not in the protocol. Alternatively, an explanation of why a DMC is not needed	NA
	21b	Description of any interim analyses and stopping guidelines, including who will have access to these interim results and make the final decision to terminate the trial	NA
Harms	22	Plans for collecting, assessing, reporting, and managing solicited and spontaneously reported adverse events and other unintended effects of trial interventions or trial conduct	13
Auditing	23	Frequency and procedures for auditing trial conduct, if any, and whether the process will be independent from investigators and the sponsor	NA
**Ethics and dissemination**			

Research ethics approval	24	Plans for seeking research ethics committee/institutional review board (REC/IRB) approval	13
Protocol amendments	25	Plans for communicating important protocol modifications (eg, changes to eligibility criteria, outcomes, analyses) to relevant parties (eg, investigators, REC/IRBs, trial participants, trial registries, journals, regulators)	13
Consent or assent	26a	Who will obtain informed consent or assent from potential trial participants or authorised surrogates, and how (see Item 32)	10, 11, 13
	26b	Additional consent provisions for collection and use of participant data and biological specimens in ancillary studies, if applicable	NA
Confidentiality	27	How personal information about potential and enrolled participants will be collected, shared, and maintained in order to protect confidentiality before, during, and after the trial	13, 19, 20
Declaration of interests	28	Financial and other competing interests for principal investigators for the overall trial and each study site	20
Access to data	29	Statement of who will have access to the final trial dataset, and disclosure of contractual agreements that limit such access for investigators	19, 20
Ancillary and post-trial care	30	Provisions, if any, for ancillary and post-trial care, and for compensation to those who suffer harm from trial participation	NA
Dissemination policy	31a	Plans for investigators and sponsor to communicate trial results to participants, healthcare professionals, the public, and other relevant groups (eg, via publication, reporting in results databases, or other data sharing arrangements), including any publication restrictions	13, 20
	31b	Authorship eligibility guidelines and any intended use of professional writers	NA
	31c	Plans, if any, for granting public access to the full protocol, participant-level dataset, and statistical code	20

Appendices

For peer review only

Informed consent materials	32	Model consent form and other related documentation given to participants and authorised surrogates	NA<br>We plan to post these on the study website
Biological specimens	33	Plans for collection, laboratory evaluation, and storage of biological specimens for genetic or molecular analysis in the current trial and for future use in ancillary studies, if applicable	NA

*It is strongly recommended that this checklist be read in conjunction with the SPIRIT 2013 Explanation & Elaboration for important clarification on the items. Amendments to the protocol should be tracked and dated. The SPIRIT checklist is copyrighted by the SPIRIT Group under the Creative Commons "Attribution-NonCommercial-NoDerivs 3.0 Unported" license.

For peer review only

BMJ Open

Family-focused intervention to promote adolescent mental health and well-being in Moldova and North Macedonia (FLOURISH): Feasibility study protocol

Journal:	*BMJ Open*
Manuscript ID	bmjopen-2023-080400.R2
Article Type:	Protocol
Date Submitted by the Author:	23-Oct-2023
Complete List of Authors:	Shenderovich, Yulia; Cardiff University, Centre for the Development, Evaluation, Complexity and Implementation in Public Health Improvement (DECIPHer), School of Social Sciences; Cardiff University, Wolfson Centre for Young People's Mental Health<br>Piolanti, Antonio; University of Klagenfurt, Institute of Psychology<br>Babii, Viorel; Asociatia Obsteasca Sanatate Pentru Tineri (Health for Youth Association)<br>Calovska-Hertzog, Nevena; Singidunum University, Department for Psychology, Faculty for Media and Communication; AST Centre for Education<br>Evans, Rhiannon; Cardiff University, Centre for the Development, Evaluation, Complexity and Implementation in Public Health Improvement (DECIPHer), School of Social Sciences<br>Heinrichs, Nina; Bielefeld University, Department of Psychology<br>Isakov, Anita Burgund; University of Belgrade, Faculty of Political Sciences, Department of Social Policy and Social Work<br>Lesco, Galina; Asociatia Obsteasca Sanatate Pentru Tineri (Health for Youth Association)<br>Moore, Graham; Cardiff University, Centre for the Development, Evaluation, Complexity and Implementation in Public Health Improvement (DECIPHer), School of Social Sciences; Cardiff University, Wolfson Centre for Young People's Mental Health<br>Mueller, Janina; University of Klagenfurt, Institute of Psychology<br>Raleva, Marija; Institute for Marriage, Family and Systemic Practice—ALTERNATIVA; Ss Cyril and Methodius University in Skopje, Department of Child and Adolescent Psychiatry<br>Shimbov, Bojan; Jaume I University Department of Economics, Instituto de Economía Internacional<br>Simon, Judith; Medical University of Vienna, Department of Health Economics, Center for Public Health; Oxford University, Department of Psychiatry, University of Oxford, Warneford Hospital<br>Waller, Franziska; University of Klagenfurt, Institute of Psychology<br>Wienand, Dennis; Medical University of Vienna, Department of Health Economics, Center for Public Health<br>Foran, Heather; University of Klagenfurt, Institute of Psychology
**Primary Subject Heading**:	Mental health

Secondary Subject Heading:	Global health, Mental health, Health services research, Public health
Keywords:	Adolescents < Adolescent, PUBLIC HEALTH, MENTAL HEALTH

SCHOLARONE™ Manuscripts

Family-focused intervention to promote adolescent mental health and well-being in Moldova and North Macedonia (FLOURISH): Feasibility study protocol

Yulia Shenderovich [1,2], Antonio Piolanti [3], Viorel Babii [4], Nevena Calovska-Hertzog [5,6], Rhiannon Evans [1], Nina Heinrichs [7], Anita Burgund Isakov [8], Galina Lesco [4], Graham Moore [1,2], Janina Mueller [3], Marija Raleva [9,10], Bojan Shimbov [11], Judit Simon [12,13], Franziska Waller [3], Dennis Wienand [12], Heather M. Foran [3]

1. Centre for the Development, Evaluation, Complexity and Implementation in Public Health Improvement (DECIPHer), School of Social Sciences, Cardiff University, Maindy Road, Cathays, Cardiff, CF24 4HQ, UK
2. Wolfson Centre for Young People's Mental Health, Cardiff University, Maindy Road, Cathays, Cardiff, CF24 4HQ, UK
3. Institute of Psychology, University of Klagenfurt, Universitaetstrasse 99, 9020 Klagenfurt am Woerthersee, Klagenfurt, Austria
4. Asociatia Obsteasca Sanatate Pentru Tineri (Health for Youth Association), Strada Socoleni 19, Chisinau 2020, Moldova
5. Department for Psychology, Faculty for Media and Communication, Singidunum University, Karadjordjeva 65, Belgrade, 11000, Serbia
6. AST Centre for Education, Djordja Kratovca 67, Belgrade, 11000, Serbia
7. Department of Psychology, Bielefeld University, Universitätsstrasse 25, 33615 Bielefeld, Germany
8. Faculty of Political Sciences, Department of Social Policy and Social Work, University of Belgrade, Jove Ilica 165, Belgrade, 11000, Serbia
9. Institute for Marriage, Family and Systemic Practice—ALTERNATIVA, Buld. Jane Sandanski 65, Skopje, 1000, North Macedonia
10. Department of Child and Adolescent Psychiatry, St. Cyril and Methodius University, Skopje, North Macedonia
11. Instituto de Economía Internacional, University Jaume I Castellon, Av. Vicent Sos Baynat, s/n 12071 Castellón de la Plana, Spain
12. Department of Health Economics, Center for Public Health, Medical University of Vienna, Kinderspitalgasse 15, 1090, Vienna, Austria
13. Department of Psychiatry, University of Oxford, Warneford Hospital, Oxford, OX3 7JX, UK

Yulia Shenderovich: ORCID 0000-0002-0254-3397; shenderovichy@cardiff.ac.uk;
Antonio Piolanti: ORCID 0000-0002-0613-0696; Antonio.Piolanti@aau.at
Viorel Babii: viorel.babii@gmail.com
Nevena Calovska-Hertzog: nevena.calovska@gmail.com
Rhiannon Evans: ORCID 0000-0002-0239-6331 ; EvansRE8@cardiff.ac.uk
Nina Heinrichs: ORCID 0000-0002-8301-5798 ; nina.heinrichs@uni-bielefeld.de
Anita Burgund Isakov: ORCID 0000-0002-4547-4934 ; anita.burgund@gmail.com
Galina Lesco: galina.lesco@gmail.com
Graham Moore: ORCID 0000-0002-6136-3978 ; MooreG@cardiff.ac.uk
Janina Mueller: ORCID 0000-0002-5005-2218 Janina.Mueller@aau.at
Marija Raleva: ORCID 0000-0002-5636-2612 ; marijaraleva@gmail.com
Bojan Shimbov: ORCID 0000-0002-0316-2430 ; shimbov@uji.es
Judit Simon : ORCID 0000-0001-9279-8627 ; judit.simon@meduniwien.ac.at
Franziska Waller: ORCID 0000-0002-4576-4325 ; Franziska.Waller@aau.at
Dennis Wienand : ORCID 0000-0002-5183-1352 ; dennis.wienand@meduniwien.ac.at

For peer review only

For peer review only - http://bmjopen.bmj.com/site/about/guidelines.xhtml

Heather M. Foran : ORCID 0000-0001-6659-5500; heather.foran@aau.at

Corresponding author: Yulia Shenderovich; address: spark, Maindy Road, Cathays, Cardiff, CF24 4HQ, UK; phone: +447774955977; email: shenderovichy@cardiff.ac.uk

Keywords: adolescents, parenting, mental health, adaptation, implementation science

For peer review only

For peer review only - http://bmjopen.bmj.com/site/about/guidelines.xhtml

ABSTRACT

Introduction
FLOURISH project will adapt, implement, and evaluate a programme to support adolescent mental health and well-being through strategies, such as strengthening parenting practices, adolescent-caregiver relationships, adolescent and parent socioemotional skills, and social support.

Methods and analysis
The project will focus on adolescents aged 10-14 and their caregivers in North Macedonia and Moldova. The countries were selected based on implementation readiness of two organisations and a need for accessible evidence-informed services to help mitigate health risks due to economic, social, and political challenges. Parenting for Lifelong Health (PLH) for Parents and Teens is a family-based programme developed for low-resource settings. PLH has been adapted with input from advisory groups. The programme includes additional components to strengthen impacts on adolescents: adolescent mental health tools, based on UNICEF's Helping Adolescents Thrive, adolescent peer support, and participation booster.

This pilot is first of three study phases. The pilot will be a feasibility testing of the adapted intervention and the assessment and implementation procedures to determine further refinements. The pilot will examine if the adapted programme is acceptable for adolescents, their families, and providers; explore contextual factors relevant to embedding this programme into longer-term scale-up; whether the programme can be delivered with fidelity and participation; whether the participants report changes in adolescent emotional and behaviour problems, well-being, and other outcomes; and whether the study tools are feasible and appropriate. Pre-post adolescent and caregiver questionnaires will provide outcome data. Process evaluation will include attendance and fidelity data, and focus groups. We will examine delivery cost and resource requirements.

Ethics and dissemination
The study was approved at the University of Klagenfurt (Austria), Medical Faculty at St. Cyril and Methodius University (North Macedonia), and National Committee of Ethical Expertise for Clinical Trials (Moldova). Through stakeholder engagement and dissemination, FLOURISH will advance scale-up of open-source family interventions.

Registration details
Trial registration: ID101095528; project page: https://www.flourish-study.org/about.html

For peer review only

For peer review only - http://bmjopen.bmj.com/site/about/guidelines.xhtml

ARTICLE SUMMARY

Strengths and limitations of this study

- This pilot study is embedded in a project designed to address a gap in evidence-informed programmes to promote adolescent mental health and well-being in North Macedonia and Moldova, at a time when migration, threat of war, and economic challenges exert particular pressures on adolescents and their families.
- The study is adapting an open-access intervention to new contexts, with a focus on scalability, to identify a model for adolescent-parent programmes that can be embedded in long-term delivery to provide a high-quality and accessible services in both countries.
- The pilot evaluation has a relatively small sample size and therefore cannot provide definitive information on the effects of the programme and its components; however, it will provide initial results on the feasibility of the intervention, its implementation and evaluation; effectiveness will be evaluated in further phases with a randomised design.

For peer review only

Family-focused intervention to promote adolescent mental health and well-being in Moldova and North Macedonia (FLOURISH): Feasibility study protocol

Word count: 4,014

INTRODUCTION

Adolescence is a period of transition and transformation, when one can acquire and strengthen factors contributing to lifelong health (1). Mental health problems during early adolescence are a global concern as this is when about half of mental health problems emerge (2). Stressful experiences in early adolescence are more strongly associated with a shorter total life span than such stressors in other phases of childhood, suggesting early adolescence is a sensitive period, making it a crucial time for providing additional support (3).

Having supportive relationships is associated with good health (4). The relationships adolescents have with their parents can remain influential, even as peers become increasingly important. Parents or caregivers are defined broadly to include any caregiver providing significant care to an adolescent, not limited to a biological parent (5). Parenting programmes focusing on the adolescent-caregiver relationships and parenting practices are recommended to support adolescent mental health (6–8). Furthermore, the WHO Guidelines on mental health promotive and preventive interventions for adolescents also recommend interventions for adolescents' interpersonal and emotional regulation skills (9).

A meta-analysis found no significant difference in effectiveness between transported and homegrown parenting interventions for reducing disruptive child behaviour (10). However, culturally adapted parenting interventions, both homegrown and transported, were more effective than non-adapted interventions (11). Adapting interventions can help maintain effectiveness while avoiding the cost of developing new interventions (12). Although majority of adolescents live in low- and middle-income countries (LMICs), research with adolescents in most LMICs is very limited (13), so evidence often has to be adapted from elsewhere.

The Family-Focused Adolescent & Lifelong Health Promotion (FLOURISH) project (2023-2026) is focused on adapting, optimising, and evaluating an intervention package for adolescents aged 10-14 and their caregivers in the Republic of North Macedonia and the Republic of Moldova (henceforth North Macedonia and Moldova). The intervention package aims to support adolescent mental health and well-being through building adolescents' and caregivers' skills, such as problem-solving and emotional regulation, strengthening social support, adolescent-caregiver relationships and communication, and improving parenting practices.

Moldova and North Macedonia were selected based on high risks for adolescent mental health, including adverse childhood experiences and poverty; lack of evidence-informed and accessible provision of prevention programmes for families with adolescents, paired with high levels of implementation readiness in two health networks. North Macedonia and Moldova are middle-income countries, among the poorest in Europe (14). Both have experienced rapid social, political, and economic transformation since 1990s following the fall of Communism.

Studies found high rates of physical punishment, emotional violence and neglect against children in both countries (15–17). Work migration often leads caregivers to spend time away from children, which can hamper communication (5). Young people also face limited work opportunities. Existing challenges were magnified by the coronavirus pandemic (18–20), and the war in Ukraine. There is stigma around help-seeking, particularly regarding mental health and well-being (21). A recent report found that in this region support services

For peer review only - http://bmjopen.bmj.com/site/about/guidelines.xhtml

for caregivers of adolescents, such as parenting workshops or support groups, are scarce, and highlighted the need for more such services (5), which our project aims to help address.

This protocol describes the adapted intervention, designed to promote adolescent mental health and well-being in Moldova and North Macedonia, and introduces the FLOURISH feasibility pilot (2023-24), which will inform the next phases of a larger study by providing information for further refinement of the intervention, and its implementation and evaluation procedures.

METHODS

Research questions and frameworks

FLOURISH is shaped by the Multiphase Optimization Strategy (MOST) framework. MOST is designed to optimise an intervention package within the key constraints in three phases – Preparation, Optimization, and Evaluation (22,23). This pilot study corresponds to the Preparation phase, focused on preparing and piloting the intervention and its evaluation. Following the pilot, a factorial trial will be conducted to select the most effective and efficient treatment package (Optimization phase). In the Evaluation phase, the revised programme package will be tested in an implementation-effectiveness randomised controlled trial.

Next, we discuss the research questions and additional frameworks and guidelines used in answering them (see Table 1 for an overview).

1. Is the adapted programme acceptable for adolescents, their caregivers, and staff in North Macedonia and Moldova, and what further adaptation are needed?

Programme materials have been adapted prior to piloting and will be revised throughout the project. FLOURISH draws on ADAPT adaptation guidance, which emphasises improving intervention fit with a new context while preserving key intervention functions (12,24).

2. What contextual factors may influence embedding of the programme into sustainable delivery and funding mechanisms in North Macedonia and Moldova?

FLOURISH is guided by the principle of "beginning with the end in mind" in ExpandNET/ World Health Organization (WHO) guidance (25). We will adapt the programme alongside developing a scaling-up strategy. This includes building capacity for scale-up and making choices to support institutionalization and expansion to more settings.

3. Can the adapted programme be delivered with high fidelity and family participation?

The pilot includes a process evaluation, guided by UK MRC process evaluation guidance (26), which focuses on implementation processes, potential mechanisms of impact, and contextual factors. To organise our understanding of the country context, we are using the Context and Implementation of Complex Interventions (CICI) framework (27).

4. What are the changes, if any, reported by the adolescents and caregivers on the primary outcomes of adolescent emotional problems, behaviour problems, and well-being, and on the secondary outcomes for adolescents and caregivers?

Primary programme targets are adolescent mental health and well-being. The pilot study will assess pre-post changes in the intervention outcomes reported by adolescents and their caregivers, following lessons learned in a previous pilot feasibility study, on which this project builds (28,29).

5. Are the study tools and their translations feasible and appropriate?

For peer review only - http://bmjopen.bmj.com/site/about/guidelines.xhtml

The pilot will examine the feasibility and preliminary psychometric performance of the outcome measures and their translations. A rigorous translation and evaluation process will be followed. The pilot will provide insights on the feasibility of the procedures and tools, and inform the next phase of the project, including which tools are retained, removed, or modified.

Table 1. Overview of the study design

Data	Participants	Research question	Framework
Advisory group consultations; focus groups	Adolescents, caregivers, staff, and (only for advisory groups) external professionals	1	ADAPT; process evaluation guidelines
Interviews	External professionals	2	CICI; ExpandNet
Attendance registers; observations; focus groups	Staff	3	Process evaluation guidelines
Questionnaires	Adolescents and caregivers	4	MOST
Think-aloud interviews; questionnaires	Adolescents and caregivers	5	N/A

Study sites

The study is conducted in two country-wide health networks, Institute for Marriage, Family and Systemic Practice (ALTERNATIVA) and Health for Youth Association. ALTERNATIVA is a network of psychologists, social workers, and family therapists in North Macedonia. Health for Youth Association in Moldova is an organisation which supports the activity of publicly funded youth-friendly clinics offering prevention and treatment in youth sexual and reproductive health, mental health, substance use, and violence prevention. Both organisations work with caregivers and adolescents, have delivered Parenting for Lifelong Health (PLH) programs, and participated in evaluations (28).

Study design

Intervention programme

The core programme examined in FLOURISH is PLH for Parents and Teens. It is a group behavioural programme (30,31), based on social learning theory (32). PLH was developed for LMICs in collaboration with UNICEF, WHO, and other international organisations and universities. Programme materials are focused on strengthening psychosocial skills and relationships, and are freely available online: https://www.who.int/teams/social-determinants-of-health/parenting-for-lifelong-health.

PLH programmes, primarily for caregivers with young children (aged 2-9), have been evaluated in multiple studies, including completed and ongoing trials in South Africa, Philippines, El Salvador, Lesotho, North Macedonia, Moldova, Romania, Thailand, Uganda, and Zambia. Evaluations reported high levels of engagement by families, practitioners, and other stakeholders. A pilot feasibility study and two large randomised trials in North Macedonia, Moldova, and Romania found improvements in parenting behaviours and child mental health (29,33), and further results are forthcoming (Foran et al.; Heinrichs et al.). PLH

For peer review only - http://bmjopen.bmj.com/site/about/guidelines.xhtml

for Parents and Teens (aged 10-17) has been evaluated in South Africa in two pre-post studies and an RCT (552 families, 40 clusters), with an embedded process evaluation (34,35). At 5-9 months after the 14-week intervention, caregivers in the intervention group, but not adolescents, reported lower rates of violence towards their adolescents and corporal punishment, and both caregivers and adolescents reported greater involved parenting and supervision of the adolescent by the caregiver (primary outcomes). There was no intervention effect detected on adolescent depression, suicidality, and externalising behaviour – however, these secondary outcomes were not the focus of the intervention.

Adaptation of the intervention

The adapted programme is drawing on the PLH manual used in the South African evaluation and on other programme versions. The initial adaptations have been informed by the input of advisory groups and expert interviews (described below), team's clinical expertise, and input from the programme developers. The adapted programme will be tested in the pilot (see the initial programme theory in Figure 1, to be revised throughout the project).

To support scalability, the programme was condensed into six group sessions, following on an introductory meeting with the caregiver and adolescent. As in the original model, sessions in FLOURISH will be delivered to a group of 10 adolescent-caregiver pairs. Each two-hour weekly session starts jointly, with a circle share, home activity discussion, agenda overview, physical exercises and games. Next, in the core lesson, a new skill is introduced and practiced, on topics developmentally relevant to adolescence. For the core lesson, the group is often split up into adolescent and caregiver parallel sessions to allow participants to share openly (see Table 2). After discussing the core lesson within the respective peer groups, participants come together and are encouraged to share the summary of their separate discussions in the joint group, and then further discuss lessons learned at home. Participants are given home practice tasks to do between the sessions. For participants who miss a session, facilitators offer a brief catch-up call or meeting. Facilitators and supervisors receive brief training in the programme and facilitation skills. Supervisors are trained in supervision. The programme is delivered by two facilitators per group, coached weekly by supervisors to promote quality of delivery. Given the focus on scalability, staff are not required to have a specific degree.

For peer review only

Table 2. *Adapted intervention structure using PLH material*

Week	Topic	Caregiver & adolescents
Pre-program	Individual meeting or call with facilitators to introduce the programme	Joint
1	Introducing the programme & ground rules. Psychoeducation about transitioning from a child to an adolescent: expectations, norms, and developmental stages	Separate
2	Relationship skills. Building a positive relationship through spending time together & praising each other	Joint
3	Talking about emotions and sensitive topics (sexuality, body changes, and intimate relationships)	Separate
4	Coping with difficult feelings (anger and stress)	Separate
5	Solving problems together as a family. How to communicate and solve problems around disagreements without conflict	Separate
6	Establishing rules and routines	Separate

To strengthen the impacts on adolescent outcomes, the adaptation of the programme involves supplementary components. Three components have been combined with the PLH programme into an intervention package: adolescent mental health tools, adolescent peer support, and adolescent participation booster.

Adolescent mental health tools. To strengthen adolescent mental health, we draw on the Helping Adolescents Thrive toolkit (HAT), designed to promote positive mental health, prevent mental health problems, and reduce engagement in self-harm and risk behaviours (36). This toolkit was developed based on a systematic review of universally delivered psychosocial interventions for adolescents (13), conducted to inform WHO Guidelines (9). The HAT materials will be delivered as six comics chapters, a workbook for adolescents and leaflets for caregivers, including, respectively, tips for adolescents to share their thoughts and feelings with their caregivers and questions caregivers can ask adolescents. The aim of these materials is to support adolescents and caregivers to communicate about adolescent mental health and well-being.

Adolescent peer support. Even brief interventions can foster friendships and social connections (37,38). Since parenting programmes have traditionally focused on parents, they have demonstrated positive impacts on social support experienced by parents but not by adolescents, including in the previous trial of PLH for Parents and Teens (30). The facilitators will pair up adolescents in the group for facilitating peer support and social connection. The PLH programme already includes peer pairings, but its potential effects have not yet been evaluated, so more emphasis will be placed on pairing up adolescents, and this will be tested as a separate component in the Optimization phase.

Adolescent participation booster. Although parenting programmes have been found acceptable in diverse settings, there is incomplete attendance (39–41). While many studies have explored parental attendance for parents of younger children (29), there is limited research on enhancing engagement of adolescents and their caregivers as programmes rarely included adolescents. The South African trial found 50-64% session attendance (42). We will investigate whether an adolescent participation booster promotes attendance.

Patient and public involvement

Members of the public were involved in the design of this research. In particular, intervention adaptations have been informed by advisory group consultations conducted in May-July 2023, as described below.

For peer review only - http://bmjopen.bmj.com/site/about/guidelines.xhtml

Advisory groups. Advisory groups of adolescents, caregivers, implementation organisation staff, and other professional experts (6-8 people per group) were formed in North Macedonia and Moldova to advise on programme adaptations and the study findings. Consultations will continue throughout the study, with key discussion points captured by a note-taker. Adolescents are eligible if they are aged 10-14 when joining the advisory group and provide adolescent assent and caregiver consent. For caregivers, staff, and other professionals, members need to be 18 or older and provide consent. For the caregiver advisory group, participants need to be caregivers of adolescents aged 10-17 (extended age range to facilitate recruitment). The groups aim to include individuals with experience of PLH or other family programmes and individuals who represent diverse perspectives (e.g., male and female caregivers, ethnic minorities). Implementation staff groups consist of ALTERNATIVA and Health for Youth Association staff. External professional experts include representatives from Ministries of Health, social services, youth, universities, research organisations, healthcare financing agencies, local NGOs, community, and international organisations implementing programmes for young people.

Advisory group analysis. Summary notes were combined into a matrix, using thematic framework and content analysis (43,44). We examined similarities and differences within and between stakeholder groups and countries. Advisory group participants received a summary for feedback as a means of validation.

Expert interviews. In addition, in each country, ten semi-structured audio-recorded interviews with professional experts took place to map the contextual facilitators, and barriers to scaling up a programme package prepared by the FLOURISH project. We drew on recommendations for qualitative sample sizes (45,46).

Interview analysis. We are using thematic and framework analysis for the transcripts. The CICI framework provides initial coding domains, with country-specific domains generated from the data. The analysis is conducted in two stages – first, a rapid synthesis to inform the intervention adaptations for the pilot (completed); second, a detailed analysis, generating a conceptual map of key contextual factors, presented as a narrative summary (ongoing).

Insert Figure 1 here

Figure 1. Initial programme theory

Piloting of the intervention

The pilot (October 2023-January 2024) will test the adapted intervention in a pre-post uncontrolled study with an outcome and process evaluation, described below.

Participants and recruitment. The pilot will include three intervention groups per country, so the target sample size is 60 adolescents and 60 caregivers (60 dyads). Recruitment will involve disseminating information through online and print materials, word-of-mouth, and via the existing clients of each network. At least one caregiver and adolescent per family will participate in the study. Inclusion criteria for caregivers are age 18 or older at baseline assessment; primary caregiver responsible for the care of an adolescent 10-14, resident in the same household at least four nights a week in the previous month; able to speak one of the local languages, in which the programme will be offered; agreement to participate in the programme; provision of consent for self and adolescent to participate in the study. Inclusion criteria for adolescents: age 10-14 at baseline assessment; assent to participate; caregiver consent.

The caregiver can be any adult who is primarily responsible for looking after the adolescent and available to participate. In cases where there is more than one caregiver who is available, they will need to agree who will be the primary and secondary participant.

For peer review only - http://bmjopen.bmj.com/site/about/guidelines.xhtml

Similarly, if two adolescents in the same age range in a family would like to join, both can join. Everyone who joins the study, both primary and secondary participants, will be invited to complete assessments.

Taking a proportionate universalism approach (47), all families will be eligible, but we will make greater efforts to include more vulnerable groups. We conceptualise our approach as selected prevention, targeted to individuals whose risk is above average, without screening (48). FLOURISH has no exclusion criteria. While introducing the project, the researchers will guide the participants through a consent form that asks whether they are currently experiencing acute distress, or a mental or physical health condition that would interfere with participation. The judgement will be made by the potential participants. If they decide they are not able to take part, the research team will follow up and provide referrals for other services (see also Ethics section).

To deliver the programme, facilitators and supervisors will be recruited from existing staff and networks of the implementing organisations. Inclusion criteria for facilitators are age 18 or older; participation in training; agreement to deliver the programme; provision of study consent. For supervisors: age 18 or older; participation in training; agreement to provide supervision; provision of consent.

Pre-post outcomes. Outcome data collection with families will be done primarily using computer-assisted self-interviewing questionnaires on tablets. Trained assessors will check participant eligibility as part of the informed consent procedure and assist adolescents and caregivers with completing the questionnaires. See Table 3 for the primary outcome measures and Table 1s (supplementary) for secondary outcome measures and Table 2s for other measures (pre-registered in clinical trials registry; ID101095528).

Outcome measures without available official translations will be adapted and translated following best practices (49). First, a cultural evaluation (relevance, appropriateness) of each item and forward-translation will be conducted by a professional with mental health experience. Second, a back-translation to English will be carried out by another translator. Third, an additional translator will compare the translations and assess correspondence between semantic equivalence and words, highlighting any discrepancies. A final translation will be produced in a meeting between the translators. All translators will be bilingual.

Think-aloud interviews will be conducted for the primary outcome measures that have not been previously used with adolescents or in the study countries (RCADS, WHO-5), and for the health economic measures that will be used in the Optimization and Evaluation phases – EQ-5D-5L (50), EQ-5D-Y (51), OxCAP-MH (52,53), PECUNIA RUM (54). Think-aloud interviews will be conducted after post-test assessments and explore item comprehension (10 interviews per country with adolescents and caregivers in Romanian and Macedonian).

Table 3. Primary Outcomes

Construct	Respondent	Measure
Adolescent emotional problems	Adolescent	- The Revised Child Anxiety and Depression Scales (RCADS), anxiety and depression subscales (55–57)<br>- Child Behaviour Checklist 6-18, internalising subscale (58)<br>- Youth Self-Report 11-18, internalising subscale (58)
Adolescent behaviour problems	Adolescent	Youth Self-Report, rule-breaking and aggressive behaviour
	Caregivers	Child Behaviour Checklist, rule-breaking and aggressive behaviour
Adolescent well-being	Adolescent	World Health Organization-Five Well-Being Index (WHO-5) (59,60)

For peer review only - http://bmjopen.bmj.com/site/about/guidelines.xhtml

Pre-post outcomes analysis. We will examine descriptive and preliminary psychometric statistics, such as internal consistencies. Intent-to-treat analyses will examine pre-post changes in adolescent and caregiver outcomes. We hypothesise that adolescent emotional problems and adolescent behaviour problems, both in self- and caregiver report, will be significantly reduced at the post-assessment; adolescent well-being will be significantly increased at the post-assessment, with medium to large effect sizes (Cohen's d => .5). We also will examine pre-post change in secondary outcomes and hypothesise small to medium effect sizes. Missing data will be addressed using full information maximum likelihood or multiple imputation methods.

Process evaluation. Facilitators will collect weekly attendance registers. Programme supervisors will conduct fidelity assessments. One session per facilitator will be observed live or video-recorded and assessed using the recording with the Facilitator Assessment Tool (61). Facilitators and supervisors will complete questionnaires to provide information on demographics and previous experience, and to collect pre-post data on staff well-being and parenting stress (for staff who are caregivers). The sample sizes are determined by the delivery model and the number of families in the intervention study.

Process evaluation analysis. We will use descriptive statistics, summarizing enrolment, attendance, fidelity, and facilitator characteristics. We will check variation in attendance by participant baseline characteristics (e.g., gender) and by intervention delivery characteristics (e.g., study site) to surface any emerging inequalities. We will explore whether attendance varied across sessions. We will explore staff responses to the pre-post well-being questionnaires using descriptives and paired t-tests. This will help inform, for example, whether additional support is needed if staff well-being had reduced.

Intervention costs. Facilitators and supervisors will complete questionnaires to capture the time and other resources spent preparing for, delivering, and following up on intervention activities. They will complete one form for the initial training and weekly forms designed to separate the resources required for the intervention versus the study.

Costs analysis. We will assess if weekly surveys are feasible. We will conduct descriptive analyses, summarizing information on time, money, and other resources used. Resource use information will be costed with country-specific unit costs collected primarily for the study to provide overall cost data. Cost information will be used to inform scale-up planning, and, in later phases, for programme cost-effectiveness analyses.

Focus groups. To inform further programme adaptations and answer process evaluation questions about the context, how the programme was implemented, and its mechanisms of change, we will conduct participant focus groups. A sub-sample of the adolescents, caregivers, and intervention staff will be recruited to participate in post-program audio-recorded focus groups (6-8 people/group, minimum 3 groups/country). We will aim to select participants with diverse backgrounds and experiences.

Focus group analysis. Focus group transcripts will be analysed using thematic and framework analysis, with codes developed both based on the initial questions and unexpected insights.

For peer review only - http://bmjopen.bmj.com/site/about/guidelines.xhtml

ETHICS AND DISSEMINATION

An ethical self-assessment for Horizon Europe was reviewed by the European Commission, and the project was cleared for ethics. The study was approved by the Institutional Review Board for Research Ethics at the University of Klagenfurt, Austria (coordinating site), the Ethical Commission for Human Research at the Medical Faculty at St. Cyril and Methodius University, North Macedonia, and National Committee of Ethical Expertise for Clinical Trials of the Ministry of Health, Moldova.

Participants will provide written assent (adolescents) or consent (adults). Adolescents and caregivers will receive reimbursements for transport costs, and thank-you vouchers for data collection activities, worth ~ €10. All staff will be trained to identify and handle potential distress in participants with detailed safety procedures and referral processes. Adverse event monitoring will help detect any potential harm caused by the research or the intervention. Any serious adverse events will be investigated and reported to the ethics committees and the data and safety monitoring board to consider potential changes in the project.

The FLOURISH project emphasises dissemination to stakeholders. Dissemination will include meetings, communication with media, and through print and online channels, including existing networks, FLOURISH website and social media. The focus of dissemination is Moldova and North Macedonia, and we will also be engaging stakeholders in the Eastern Europe region, working with family and adolescent health associations, NGOs, government, and international agencies.

This pilot will provide insights on the feasibility of the intervention, its implementation and evaluation, and thus inform the factorial trial planned as the next research phase, guiding which changes need to be made to the intervention, implementation, and evaluation procedures. More broadly, this project will provide insights on how best to adapt and scale up a programme targeting family relationships and adolescent mental health at a time when economic problems, interpersonal and intergroup conflict, the threat of a wider war, and other issues contribute to increased stress for both adolescents and adults. We will draw on a set of frameworks and global health best practices (62), such as using open-access interventions that involve stakeholders in all stages of intervention adaptation and evaluation.

For peer review only

REFERENCES

1. Ross DA, Hinton R, Melles-Brewer M, Engel D, Zeck W, Fagan L, et al. Adolescent Well-Being: A Definition and Conceptual Framework. Journal of Adolescent Health [Internet]. 2020;67(4):472–6. Available from: https://doi.org/10.1016/j.jadohealth.2020.06.042
2. Rice F, Sellers R, Hammerton G, Eyre O, Bevan-Jones R, Thapar AK, et al. Antecedents of new-onset major depressive disorder in children and adolescents at high familial risk. JAMA Psychiatry. 2017;74(2):153–60.
3. Falconi A, Gemmill A, Dahl RE, Catalano R. Adolescent experience predicts longevity: Evidence from historical epidemiology. Journal of Developmental Origins of Health and Disease. 2014;5(3):171–7.
4. Holt-Lunstad J. Why Social Relationships Are Important for Physical Health: A Systems Approach to Understanding and Modifying Risk and Protection. Annual Review of Psychology. 2018;69:437–58.
5. UNICEF. Parenting adolescents. A Regional Study on Parenting Adolescents and Parenting Support Programmes in Belarus, Bulgaria, Georgia, Moldova, Montenegro and Romania. 2018.
6. Pedersen GA, Smallegange E, Coetzee A, Hartog K, Turner J, Jordans MJD, et al. A Systematic Review of the Evidence for Family and Parenting Interventions in Low- and Middle-Income Countries: Child and Youth Mental Health Outcomes. Journal of Child and Family Studies. 2019;28(8):2036–55.
7. UNICEF. Programming guidance for parenting of adolescents [Internet]. 2021. Available from: https://www.unicef.org/reports/parenting-adolescents
8. Butchart A. WHO Guideline on parenting to prevent child maltreatment and promote positive development in children aged 0-17 years. 2020. 1–37 p.
9. WHO. Guidelines on mental health promotive and preventive interventions for adolescents. 2020;1–120. Available from: https://www.who.int/publications/i/item/9789240011854
10. Leijten P, Melendez-Torres GJ, Knerr W, Gardner F. Transported versus homegrown parenting interventions for reducing disruptive child behavior: A multilevel meta-regression study. Journal of the American Academy of Child and Adolescent Psychiatry [Internet]. 2016;55(7):610–7. Available from: http://dx.doi.org/10.1016/j.jaac.2016.05.003
11. Gardner F, Montgomery P, Knerr W. Transporting evidence-based parenting programs for child problem behavior (age 3–10) between countries: Systematic review and meta-analysis. Journal of Clinical Child & Adolescent Psychology [Internet]. 2015;45:749–62. Available from: http://dx.doi.org/10.1080/15374416.2015.1015134
12. Moore G, Campbell M, Copeland L, Craig P, Movsisyan A, Hoddinott P, et al. Adapting interventions to new contexts-the ADAPT guidance. The BMJ. 2021;374(fig 1).
13. Skeen S, Laurenzi CA, Gordon SL, Du Toit S, Tomlinson M, Dua T, et al. Adolescent mental health program components and behavior risk reduction: A Meta-analysis. Pediatrics. 2019;144(2).
14. World Bank. DataBank world development indicators. [Internet]. 2022. Available from: https://databank.worldbank.org/source/world-development-indicators
15. Sebre S, Sprugevica I, Novotni A, Bonevski D, Pakalniskiene V, Popescu D, et al. Cross-cultural comparisons of child-reported emotional and physical abuse: Rates, risk factors and psychosocial symptoms. Child Abuse and Neglect. 2004;28(1):113–27.
16. WHO. Survey of adverse childhood experiences in the former Yugolav Republic of

For peer review only - http://bmjopen.bmj.com/site/about/guidelines.xhtml

Macedonia [Internet]. 2013. Available from: https://www.euro.who.int/en/countries/north-macedonia/publications/more-publications/publications-archive/survey-of-adverse-childhood-experiences-in-the-former-yugoslav-republic-of-macedonia
17. WHO. Adverse childhood experiences and associated health-risk behaviours in university students from the Republic of Moldova [Internet]. 2018. Available from: https://www.euro.who.int/en/countries/republic-of-moldova/publications/adverse-childhood-experiences-and-associated-health-risk-behaviours-in-university-students-from-the-republic-of-moldova-2018
18. Bhatia A, Fabbri C, Cerna-Turoff I, Tanton C, Knight L, Turner E, et al. COVID-19 response measures and violence against children. Bulletin of the World Health Organization. 2020;98(9):583-583A.
19. Bhatia A, Fabbri C, Cerna-Turoff I, Turner E, Lokot M, Warria A, et al. Violence against children during the COVID-19 pandemic. Bulletin of the World Health Organization [Internet]. 2021 Oct 1;99(10):730–8. Available from: https://www.ncbi.nlm.nih.gov/pmc/articles/PMC8477433/pdf/BLT.20.283051.pdf
20. Katz I, Katz C, Andresen S, Bérubé A, Collin-Vezina D, Fallon B, et al. Child maltreatment reports and child protection service responses during COVID-19: Knowledge exchange among Australia, Brazil, Canada, Colombia, Germany, Israel, and South Africa. Child Abuse and Neglect [Internet]. 2021 Jun;116(August 2020):105078. Available from: https://linkinghub.elsevier.com/retrieve/pii/S0145213421001514
21. Dlouhy M. Mental health policy in Eastern Europe: A comparative analysis of seven mental health systems. BMC Health Services Research. 2014;14.
22. Collins LM. Optimization of Behavioral, Biobehavioral, and Biomedical Interventions [Internet]. Cham: Springer International Publishing; 2018. (Statistics for Social and Behavioral Sciences; vol. 22). Available from: http://link.springer.com/10.1007/978-3-319-72206-1
23. Guastaferro K, Collins LM. Optimization Methods and Implementation Science: An Opportunity for Behavioral and Biobehavioral Interventions. Implementation Research and Practice. 2021;2:263348952110543.
24. Evans RE, Moore G, Movsisyan A, Rehfuess E. How can we adapt complex population health interventions for new contexts? Progressing debates and research priorities. Journal of Epidemiology and Community Health. 2021;75(1):40–5.
25. Ghiron L, Ramirez-Ferrero E, Badiani R, Benevides R, Ntabona A, Fajans P, et al. Promoting Scale-Up Across a Global Project Platform: Lessons from the Evidence to Action Project. Global Implementation Research and Applications [Internet]. 2021;1(2):69–76. Available from: https://doi.org/10.1007/s43477-021-00013-4
26. Moore G, Audrey S, Barker M, Bond L, Bonell C, Hardeman W, et al. Process evaluation of complex interventions: Medical Research Council guidance. British Medical Journal [Internet]. 2015;350:h1258. Available from: http://www.bmj.com/cgi/doi/10.1136/bmj.h1258
27. Pfadenhauer LM, Gerhardus A, Mozygemba K, Lysdahl KB, Booth A, Hofmann B, et al. Making sense of complexity in context and implementation: The Context and Implementation of Complex Interventions (CICI) framework. Implementation Science. 2017;12(1):1–17.
28. Frantz I, Foran HM, Lachman JM, Jansen E, Hutchings J, Băban A, et al. Prevention of child mental health problems in Southeastern Europe: A multicentre sequential study to adapt, optimise and test the parenting programme “Parenting for Lifelong Health for Young Children”, protocol for stage 1, the feasibility study. BMJ Open.

For peer review only - http://bmjopen.bmj.com/site/about/guidelines.xhtml

2019;9(1).
29. Jansen E, Frantz I, Hutchings J, Lachman J, Williams M, Taut D, et al. Preventing child mental health problems in southeastern Europe: Feasibility study (phase 1 of MOST framework). Family Process. 2021;(August 2020):1–18.
30. Cluver LD, Meinck F, Steinert JI, Shenderovich Y, Doubt J, Herrero Romero R, et al. Parenting for Lifelong Health: a pragmatic cluster randomised controlled trial of a non-commercialised parenting programme for adolescents and their families in South Africa. BMJ Global Health [Internet]. 2018 Jan;3(1):e000539. Available from: http://
31. Lachman JM, Martin M, Booij A, Tsoanyane S, Majenga N. Parenting for Lifelong Health for Teens: Facilitator Assessment Tool Manual. Cape Town; 2019.
32. Bandura A. Social learning theory. New York: General Learning Press; 1977.
33. Lachman J, Heinrichs N, Jansen E, Brühl A, Taut D, Fang X, et al. Preventing child mental health problems through parenting interventions in Southeastern Europe (RISE): Protocol for a multi-country cluster randomized factorial study. Contemporary Clinical Trials [Internet]. 2019;86:105855. Available from: https://doi.org/10.1016/j.cct.2019.105855
34. Cluver LD, Meinck F, Steinert JI, Shenderovich Y, Doubt J, Herrero Romero R, et al. Parenting for Lifelong Health: A pragmatic cluster randomised controlled trial of a non-commercialised parenting programme for adolescents and their families in South Africa. BMJ Global Health [Internet]. 2018 Jan;3(1):e000539. Available from: http://
35. Shenderovich Y, Eisner M, Cluver L, Doubt J, Berezin M, Majokweni S, et al. Delivering a Parenting Program in South Africa: The Impact of Implementation on Outcomes. Journal of Child and Family Studies [Internet]. 2019 Feb;28(4). Available from: https://doi.org/10.1007/s10826-018-01319-y
36. UNICEF. HIV and AIDS in adolescents [Internet]. 2021 [cited 2021 Jul 15]. Available from: https://data.unicef.org/topic/adolescents/hiv-aids/
37. Echols L, Ivanich J. From "Fast Friends" to True Friends: Can a Contact Intervention Promote Friendships in Middle School? Journal of Research on Adolescence. 2021;31(4):1152–71.
38. Veenstra R, Laninga-Wijnen L. Peer network studies and interventions in adolescence. Current Opinion in Psychology [Internet]. 2022;44:157–63. Available from: https://doi.org/10.1016/j.copsyc.2021.09.015
39. Butler AM, Titus C. Systematic Review of Engagement in Culturally Adapted Parent Training for Disruptive Behavior. Journal of Early Intervention. 2015;37(4):300–18.
40. Chacko A, Jensen SA, Lowry LS, Cornwell M, Chimklis A, Chan E, et al. Engagement in behavioral parent training: Review of the literature and implications for practice. Clinical Child and Family Psychology Review. 2016;19(3):204–15.
41. Gonzalez C, Morawska A, Haslam DM. Enhancing Initial Parental Engagement in Interventions for Parents of Young Children: A Systematic Review of Experimental Studies. Clinical Child and Family Psychology Review [Internet]. 2018;21(3):415–32. Available from: https://doi.org/10.1007/s10567-018-0259-4
42. Shenderovich Y, Eisner M, Cluver L, Doubt J, Berezin MK, Majokweni S, et al. What affects attendance and engagement in a parenting program in South Africa? Prevention Science. 2018;19(7):977–86.
43. Gale NK, Heath G, Cameron E, Rashid S, Redwood S. Using the framework method for the analysis of qualitative data in multi-disciplinary health research. BMC Medical Research Methodology [Internet]. 2013 Dec 18;13(1):117. Available from: https://bmcmedresmethodol.biomedcentral.com/articles/10.1186/1471-2288-13-117
44. Heath G, Cameron E, Cummins C, Greenfield S, Pattison H, Kelly D, et al. Paediatric "care closer to home": Stakeholder views and barriers to implementation. Health and

For peer review only - http://bmjopen.bmj.com/site/about/guidelines.xhtml

Place [Internet]. 2012;18(5):1068–73. Available from: http://dx.doi.org/10.1016/j.healthplace.2012.05.003
45. Hennink M, Kaiser BN. Sample sizes for saturation in qualitative research: A systematic review of empirical tests. Social Science and Medicine [Internet]. 2022;292:114523. Available from: https://doi.org/10.1016/j.socscimed.2021.114523
46. Krueger RA. Focus groups: A practical guide for applied research. Sage publications; 2014.
47. Segrott J, Gillespie D, Lau M, Holliday J, Murphy S, Foxcroft D, et al. Effectiveness of the Strengthening Families Programme in the UK at preventing substance misuse in 10–14 year-olds: a pragmatic randomised controlled trial. BMJ Open. 2022;12(2):e049647.
48. National Research Council and Institute of Medicine. Preventing Mental, Emotional, and Behavioral Disorders Among Young People [Internet]. Preventing Mental, Emotional, and Behavioral Disorders Among Young People: Progress and Possibilities. Washington, D.C.: National Academies Press; 2009. Available from: https://www.ncbi.nlm.nih.gov/books/NBK32789/
49. Reichenheim M, Bastos JL. What, what for and how? Developing measurement instruments in epidemiology. Rev Saude Publica [Internet]. 2021 Aug 9;55:40. Available from: https://www.revistas.usp.br/rsp/article/view/189335
50. Herdman M, Gudex C, Lloyd A, Janssen M, Kind P, Parkin D, et al. Development and preliminary testing of the new five-level version of EQ-5D (EQ-5D-5L). Quality of Life Research [Internet]. 2011 Dec 9;20(10):1727–36. Available from: http://link.springer.com/10.1007/s11136-011-9903-x
51. Wille N, Badia X, Bonsel G, Burström K, Cavrini G, Devlin N, et al. Development of the EQ-5D-Y: a child-friendly version of the EQ-5D. Quality of Life Research [Internet]. 2010 Aug 20;19(6):875–86. Available from: http://link.springer.com/10.1007/s11136-010-9648-y
52. Vergunst F, Jenkinson C, Burns T, Anand P, Gray A, Rugkåsa J, et al. Psychometric validation of a multi-dimensional capability instrument for outcome measurement in mental health research (OxCAP-MH). Health and Quality of Life Outcomes [Internet]. 2017 Dec 28;15(1):250. Available from: https://hqlo.biomedcentral.com/articles/10.1186/s12955-017-0825-3
53. Simon J, Łaszewska A, Leutner E, Spiel G, Churchman D, Mayer S. Cultural and linguistic transferability of the multi-dimensional OxCAP-MH capability instrument for outcome measurement in mental health: the German language version. BMC Psychiatry [Internet]. 2018 Dec 5;18(1):173. Available from: https://bmcpsychiatry.biomedcentral.com/articles/10.1186/s12888-018-1762-3
54. Pokhilenko I, Janssen LMM, Paulus ATG, Drost RMWA, Hollingworth W, Thorn JC, et al. Development of an Instrument for the Assessment of Health-Related Multi-sectoral Resource Use in Europe: The PECUNIA RUM. Applied Health Economics and Health Policy [Internet]. 2023 Mar 9;21(2):155–66. Available from: https://link.springer.com/10.1007/s40258-022-00780-7
55. Ebesutani C, Reise SP, Chorpita BF, Ale C, Regan J, Young J, et al. The Revised Child Anxiety and Depression Scale-Short Version: Scale reduction via exploratory bifactor modeling of the broad anxiety factor. Psychological Assessment [Internet]. 2012;24(4):833–45. Available from: http://doi.apa.org/getdoi.cfm?doi=10.1037/a0027283
56. Krause K, Midgley N, Edbrooke-Childs J, Wolpert M. A comprehensive mapping of outcomes following psychotherapy for adolescent depression: The perspectives of young people, their parents and therapists. European Child and Adolescent Psychiatry

For peer review only - http://bmjopen.bmj.com/site/about/guidelines.xhtml

[Internet]. 2021;30(11):1779–91. Available from: https://doi.org/10.1007/s00787-020-01648-8
57. Krause KR, Chung S, Adewuya AO, Albano AM, Babins-Wagner R, Birkinshaw L, et al. International consensus on a standard set of outcome measures for child and youth anxiety, depression, obsessive-compulsive disorder, and post-traumatic stress disorder. The Lancet Psychiatry [Internet]. 2021 Jan;8(1):76–86. Available from: https://linkinghub.elsevier.com/retrieve/pii/S2215036620303564
58. Achenbach TM, Rescorla L. The Manual for the ASEBA School-Age Forms & Profiles. Burlington; 2001.
59. Topp CW, Østergaard SD, Søndergaard S, Bech P. The WHO-5 Well-Being Index: A Systematic Review of the Literature. Psychotherapy and Psychosomatics [Internet]. 2015;84(3):167–76. Available from: https://www.karger.com/Article/FullText/376585
60. WHO. Wellbeing Measures in Primary Health Care/The Depcare Project. Copenhagen; 1998.
61. Martin M, Lachman JM, Murphy H, Gardner F, Foran H. The development, reliability, and validity of the Facilitator Assessment Tool: An implementation fidelity measure used in Parenting for Lifelong Health for Young Children. Child: Care, Health and Development. 2023;49(3):591–604.
62. Patel V, Saxena S, Lund C, Thornicroft G, Baingana F, Bolton P, et al. The Lancet Commission on global mental health and sustainable development. The Lancet. 2018;392(10157):1553–98.

For peer review only

For peer review only - http://bmjopen.bmj.com/site/about/guidelines.xhtml

Funding
Funded by the European Union Horizon Europe and UK Research and Innovation. Views and opinions expressed are however those of the author(s) only and do not necessarily reflect those of the European Union or UK Research and Innovation (UKRI). Neither the European Union nor the granting authorities can be held responsible for them. This work was funded by the European Union Horizon Europe research and innovation programme under grant agreement number 101095528 and by the UKRI under the UK government's Horizon Europe funding guarantee, grant number 10050850. The funders were not and will not be involved in the design of the study, the collection, analysis and interpretation of data or the writing of the protocol.

Acknowledgements
We thank the FLOURISH research and implementation teams: facilitators, supervisors, data assessors, coordinators, as well as all adolescents, parents, staff and external experts taking part in the project. We are thankful for the support of the local organisations and stakeholders. We thank the DSMB & ethics advisers Laura Ghiron and Patty Leijten, Data Protection Officer Peter Schartner, Christian Kubb for the project website, Barbara Poecher for coordination and project management support, Joanna Lai at UNICEF, Sabine Rakotomalala at WHO, PLH development team – especially Jenny Doubt for her work on the programme adaptation as well as Jamie Lachman and Lucie Cluver. We thank Slavica Gajdadzis-Khezhevikj, Maite Alguacil Marí as study co-investigators. For research assistance, we thank Lara, Barg, Katharina Buss, Mackenzie Martin, Bethan Pell, Abbey Rowe, Eve Ruet, Swetha Sampathkumar and other researchers.
RE, GM & YS are supported by DECIPHer, which is funded by Welsh Government through Health and Care Research Wales. GM and YS are also funded by the Wolfson Centre for Young People's Mental Health. The Wolfson Centre for Young People's Mental Health has been established with support from the Wolfson Foundation.
For the purpose of open access, the authors have applied a CC BY public copyright licence to any Author Accepted Manuscript version arising.

Author statement
HMF and YS led the preparation of the grant proposal and the design of the study. NC, RE, HMF, NC, NH, GL, GM, MR, JS, BS and YS, are PIs on the study and contributed to the conception and implementation of the study, including data collection. AB, VB, SG-K, JM, AP, DW, FW are co-Is and contributed to the conception and implementation of the study, including data collection. NH has been leading the adaptation of the intervention programme, with support from team members, in particular VB, AB, SG-K and MR. MR and SG-K have designed and delivered training in the intervention programme. HMF, NH, JM, AP, YS, FW have designed and delivered training in data collection and safety monitoring. HMF, JM, AP, and FW are key contributors to the design of the adolescent and caregiver data collection, analysis, and management, and oversee the project data management and ethical processes. RE, GM,YS are key contributors to the design of the process evaluation and advisory group data collection, analysis, and management. JS and DW designed the health economic analysis, oversaw the relevant toolkit development, assessment, analysis and cultural adaptation, and collaborated in the intervention cost evaluation. HMF is the study Coordinator. All authors have revised the manuscript, have approved the final version of the manuscript to be published, and have agreed to be accountable for all aspects of the work.

Data sharing statement

For peer review only - http://bmjopen.bmj.com/site/about/guidelines.xhtml

The project will make some anonymized quantitative datasets available via an open-access repository following the FAIR (Findable, Accessible, Interoperable, Reusable) principles. The research team will ensure that results will be published in open access peer-reviewed publications or are made available in other ways, such as through pre-prints. Contact heather.foran@aau.at for queries related to overall data management plan and access. The research team will ensure that results will be published in open access peer-reviewed publications or are made available in other ways, such as through pre-prints.

Competing interests (conflicts of interest)
VB, HMF, NH, SG-K, GL, JM, MR, AP, YS, FW report participating in other research studies involving the PLH programme as investigators and team members. NH reports personal fees from academic institutions offering continued education for psychotherapy education in Germany, outside the submitted work; and NH has attended trainings in Triple P and PCIT. YS was involved in the South African trial, which is the previous large-scale evaluation of the PLH for Parents and Teens. HMF, NH, GL, and MR were principal investigators on the previous Horizon European project, RISE, evaluating the PLH for Young Children in Moldova and North Macedonia. JS is developer of the OxCAP-MH and PECUNIA RUM instruments. DW has no competing interest to declare.

For peer review only - http://bmjopen.bmj.com/site/about/guidelines.xhtml

For peer review only

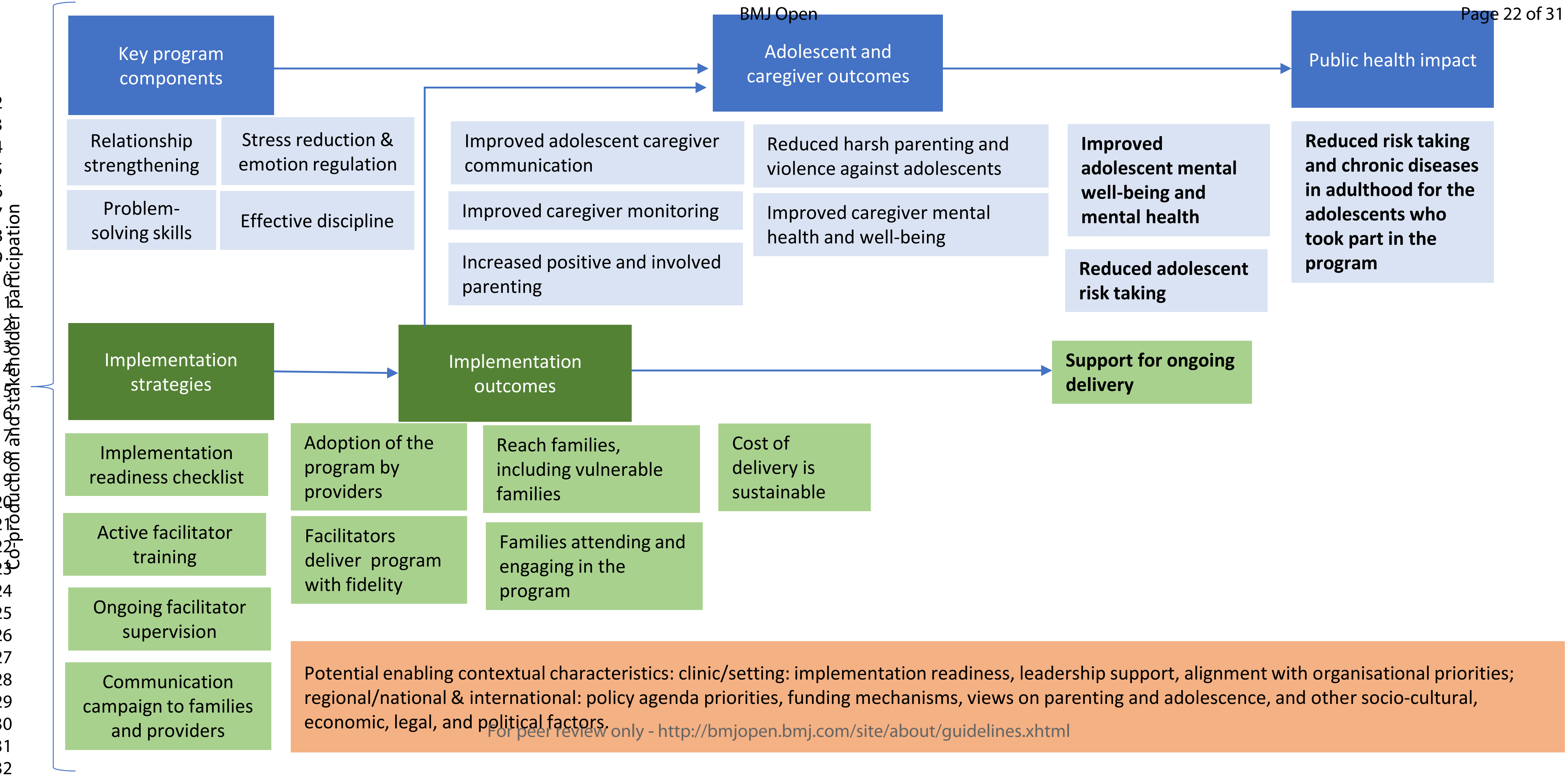

For peer review only - http://bmjopen.bmj.com/site/about/guidelines.xhtml

Supplementary materials

Table 1s Secondary outcome measures

Construct	Respondent	Measure
Communication	Adolescents & parents	Child-Parent Communication Apprehension scale, total score (1)
		Family Assessment Device-FAD, general functioning subscale (2)
Involved parenting	Adolescents & parents	Alabama Parenting Questionnaire (3), involved parenting and parental supervision/monitoring subscales.
Harsh parenting	Parents	Alabama Parenting Questionnaire (3), corporal punishment subscale.
Adolescent emotional problems	Adolescents	The Revised Child Anxiety and Depression Scales (RCADS), total score (4–6)
Adolescent behavior problems	Parents	Child Behavior Checklist 6-18, externalizing behavior (7)
Loneliness	Adolescents	UCLA-8 Loneliness scale, total score (8)
	Parents	Revised UCLA-6 Loneliness scale, total score (9)
Social support	Adolescents & parents	Medical Outcome Study Social Support Survey, emotional and affectionate sub-scales (10)
Parents wellbeing	Parents	WHO-5 Well-Being Index, total score (WHO-5) (11,12)
Parents psychological distress	Parents	The Patient Health Questionnaire – 9 (PHQ-9) , total score (13).
Parental stress	Parents	Parental Stress Scale, total score (14).

For peer review only

Table 2s. Other measures

Construct	Respondent	Measure
Enrolment	Facilitators	Percentage of participants who joined the study and have attended at least one group PLH session (individually for adolescents and caregivers, for both jointly at the dyad/family level)
Attendance	Facilitators	Percentage of group PLH sessions attended out of 6 (individually for adolescents and caregivers, for both jointly at the dyad/family level)<br>Percentage of catch-up PLH contact out of 6 (individually for adolescents and caregivers)
Adolescent healthy weight	Adolescents & parents	Body mass index (not expected to change but assessed for sample description).
Alcohol use	Parents	The Alcohol Use Disorder Identification Test (15).
Posttraumatic stress	Parents	PTSD checklist for DSM-5 (PCL-5), short form, total score
	Adolescent	Children's Revised Impact of Event Scale (16), total score and subscales
Sexual risk behaviors	Adolescents	Health Behavior in School-aged Children Study Sexual health scale, total score (17).
Adolescent-defined problems	Adolescent	The Top Problem Assessment (18–20).
Demographics	Adolescent & parents	Parent and adolescent age, gender, education, economic status.

For peer review only

REFERENCES

1. Lucchetti AE, Powers WG, Love DE. The Empirical Development of the Child-Parent Communication Apprehension Scale for Use With Young Adults. Journal of Family Communication [Internet]. 2002 Jul;2(3):109–31. Available from: http://www.tandfonline.com/doi/abs/10.1207/S15327698JFC0203_1

2. Epstein NB, Baldwin LM, Bishop DS. The McMaster family assessment device. Journal of Marital and Family Therapy [Internet]. 1983 Apr;9(2):171–80. Available from: https://onlinelibrary.wiley.com/doi/10.1111/j.1752-0606.1983.tb01497.x

3. Frick PJ. Alabama Parenting Questionnaire. Unpublished rating scale, University of Alabama. 1991;

4. Chorpita BF, Moffitt CE, Gray J. Psychometric properties of the Revised Child Anxiety and Depression Scale in a clinical sample. Behaviour Research and Therapy [Internet]. 2005 Mar;43(3):309–22. Available from: https://linkinghub.elsevier.com/retrieve/pii/S0005796704000695

5. Krause K, Midgley N, Edbrooke-Childs J, Wolpert M. A comprehensive mapping of outcomes following psychotherapy for adolescent depression: The perspectives of young people, their parents and therapists. European Child and Adolescent Psychiatry [Internet]. 2021;30(11):1779–91. Available from: https://doi.org/10.1007/s00787-020-01648-8

6. Krause KR, Chung S, Adewuya AO, Albano AM, Babins-Wagner R, Birkinshaw L, et al. International consensus on a standard set of outcome measures for child and youth anxiety, depression, obsessive-compulsive disorder, and post-traumatic stress disorder. The Lancet Psychiatry [Internet]. 2021 Jan;8(1):76–86. Available from: https://linkinghub.elsevier.com/retrieve/pii/S2215036620303564

7. Achenbach TM, Rescorla L. The Manual for the ASEBA School-Age Forms & Profiles. Burlington; 2001.

8. Roberts RE, Lewinsohn PM, Seeley JR. A Brief Measure of Loneliness Suitable for Use with Adolescents. Psychological Reports [Internet]. 1993 Jun;72(3_suppl):1379–91. Available from: http://journals.sagepub.com/doi/10.2466/pr0.1993.72.3c.1379

9. Wongpakaran N, Wongpakaran T, Pinyopornpanish M, Simcharoen S, Suradom C, Varnado P, et al. Development and validation of a 6-item Revised UCLA Loneliness Scale (RULS-6) using Rasch analysis. British Journal of Health Psychology [Internet]. 2020 May 30;25(2):233–56. Available from: https://onlinelibrary.wiley.com/doi/10.1111/bjhp.12404

10. Sherbourne CD, Stewart AL. The MOS social support survey. Social Science and Medicine. 1991;32(6):705–14.

11. Topp CW, Østergaard SD, Søndergaard S, Bech P. The WHO-5 Well-Being Index: A Systematic Review of the Literature. Psychotherapy and Psychosomatics [Internet]. 2015;84(3):167–76. Available from: https://www.karger.com/Article/FullText/376585

12. WHO. Wellbeing Measures in Primary Health Care/The Depcare Project. Copenhagen; 1998.

13. Kroenke K, Spitzer RL, Williams JBW. The PHQ-9: validity of a brief depression

For peer review only - http://bmjopen.bmj.com/site/about/guidelines.xhtml

severity measure. Journal of General Internal Medicine [Internet]. 2001 Sep;16(9):606–13. Available from: http://link.springer.com/10.1046/j.1525-1497.2001.016009606.x

14. Berry JO, Jones WH. The parental stress scale: Initial psychometric evidence. Journal of Social and Personal Relationships. 1995;12(3):463–72.

15. Saunders JB, Aasland OG, Babor TF, De la Fuente JR, Grant M. Development of the Alcohol Use Disorders Identification Test (AUDIT): WHO Collaborative Project on Early Detection of Persons with Harmful Alcohol Consumption-II. Addiction. 1993;88(6):791–804.

16. Perrin S, Meiser-Stedman R, Smith P. The Children's Revised Impact of Event Scale (CRIES): Validity as a Screening Instrument for PTSD. Behavioural and Cognitive Psychotherapy [Internet]. 2005 Oct 14;33(4):487–98. Available from: https://www.cambridge.org/core/product/identifier/S1352465805002419/type/journal_article

17. Young H, Költo A, Reis M, Saewyc EM, Moreau N, Burke L, et al. Sexual Health questions included in the Health Behaviour in School-aged Children (HBSC) Study: An international methodological pilot investigation. BMC Medical Research Methodology. 2016;16(1):1–12.

18. Milgram L, Tonarely NA, Ehrenreich-May J. Youth top problems and early treatment response to the unified protocols for transdiagnostic treatment of emotional disorders in children and adolescents. Child Psychiatry & Human Development [Internet]. 2021 Mar 17; Available from: http://link.springer.com/10.1007/s10578-021-01151-4

19. Weisz JR, Chorpita BF, Frye A, Ng MY, Lau N, Bearman SK, et al. Youth top problems: Using idiographic, consumer-guided assessment to identify treatment needs and to track change during psychotherapy. Journal of Consulting and Clinical Psychology. 2011;79(3):369–80.

20. Weisz JR. Testing standard and modular designs for psychotherapy treating depression, anxiety, and conduct problems in youth. Archives of General Psychiatry [Internet]. 2012 Mar 1;69(3):274. Available from: http://archpsyc.jamanetwork.com/article.aspx?doi=10.1001/archgenpsychiatry.2011.147

For peer review only - http://bmjopen.bmj.com/site/about/guidelines.xhtml

STANDARD PROTOCOL ITEMS: RECOMMENDATIONS FOR INTERVENTIONAL TRIALS

SPIRIT 2013 Checklist: Recommended items to address in a clinical trial protocol and related documents*

Section/item	Item No	Description	Reported in the manuscript on page
**Administrative information**			
Title	1	Descriptive title identifying the study design, population, interventions, and, if applicable, trial acronym	1
Trial registration	2a	Trial identifier and registry name. If not yet registered, name of intended registry	3
	2b	All items from the World Health Organization Trial Registration Data Set	NA
Protocol version	3	Date and version identifier	NA
Funding	4	Sources and types of financial, material, and other support	19
Roles and responsibilities	5a	Names, affiliations, and roles of protocol contributors	1,19
	5b	Name and contact information for the trial sponsor	19
	5c	Role of study sponsor and funders, if any, in study design; collection, management, analysis, and interpretation of data; writing of the report; and the decision to submit the report for publication, including whether they will have ultimate authority over any of these activities	19
	5d	Composition, roles, and responsibilities of the coordinating centre, steering committee, endpoint adjudication committee, data management team, and other individuals or groups overseeing the trial, if applicable (see Item 21a for data monitoring committee)	NA
**Introduction**			
Background and rationale	6a	Description of research question and justification for undertaking the trial, including summary of relevant studies (published and unpublished) examining benefits and harms for each intervention	5,6

For peer review only

	6b	Explanation for choice of comparators	NA
Objectives	7	Specific objectives or hypotheses	6,7
Trial design	8	Description of trial design including type of trial (eg, parallel group, crossover, factorial, single group), allocation ratio, and framework (eg, superiority, equivalence, noninferiority, exploratory)	10,12
**Methods: Participants, interventions, and outcomes**			
Study setting	9	Description of study settings (eg, community clinic, academic hospital) and list of countries where data will be collected. Reference to where list of study sites can be obtained	6,7
Eligibility criteria	10	Inclusion and exclusion criteria for participants. If applicable, eligibility criteria for study centres and individuals who will perform the interventions (eg, surgeons, psychotherapists)	10,11
Interventions	11a	Interventions for each group with sufficient detail to allow replication, including how and when they will be administered	8,9
	11b	Criteria for discontinuing or modifying allocated interventions for a given trial participant (eg, drug dose change in response to harms, participant request, or improving/worsening disease)	13
	11c	Strategies to improve adherence to intervention protocols, and any procedures for monitoring adherence (eg, drug tablet return, laboratory tests)	8 (facilitator supervision)
	11d	Relevant concomitant care and interventions that are permitted or prohibited during the trial	NA
Outcomes	12	Primary, secondary, and other outcomes, including the specific measurement variable (eg, systolic blood pressure), analysis metric (eg, change from baseline, final value, time to event), method of aggregation (eg, median, proportion), and time point for each outcome. Explanation of the clinical relevance of chosen efficacy and harm outcomes is strongly recommended	11,12 and supplementary materials
Participant timeline	13	Time schedule of enrolment, interventions (including any run-ins and washouts), assessments, and visits for participants. A schematic diagram is highly recommended (see Figure)	8,9

For peer review only - http://bmjopen.bmj.com/site/about/guidelines.xhtml

Sample size	14	Estimated number of participants needed to achieve study objectives and how it was determined, including clinical and statistical assumptions supporting any sample size calculations	10
Recruitment	15	Strategies for achieving adequate participant enrolment to reach target sample size	10
**Methods: Assignment of interventions (for controlled trials)**			
Allocation:			
Sequence generation	16a	Method of generating the allocation sequence (eg, computer-generated random numbers), and list of any factors for stratification. To reduce predictability of a random sequence, details of any planned restriction (eg, blocking) should be provided in a separate document that is unavailable to those who enrol participants or assign interventions	NA
Allocation concealment mechanism	16b	Mechanism of implementing the allocation sequence (eg, central telephone; sequentially numbered, opaque, sealed envelopes), describing any steps to conceal the sequence until interventions are assigned	NA
Implementation	16c	Who will generate the allocation sequence, who will enrol participants, and who will assign participants to interventions	NA
Blinding (masking)	17a	Who will be blinded after assignment to interventions (eg, trial participants, care providers, outcome assessors, data analysts), and how	NA
	17b	If blinded, circumstances under which unblinding is permissible, and procedure for revealing a participant's allocated intervention during the trial	NA
**Methods: Data collection, management, and analysis**			
Data collection methods	18a	Plans for assessment and collection of outcome, baseline, and other trial data, including any related processes to promote data quality (eg, duplicate measurements, training of assessors) and a description of study instruments (eg, questionnaires, laboratory tests) along with their reliability and validity, if known. Reference to where data collection forms can be found, if not in the protocol	11,12

For peer review only - http://bmjopen.bmj.com/site/about/guidelines.xhtml

	18b	Plans to promote participant retention and complete follow-up, including list of any outcome data to be collected for participants who discontinue or deviate from intervention protocols	NA
Data management	19	Plans for data entry, coding, security, and storage, including any related processes to promote data quality (eg, double data entry; range checks for data values). Reference to where details of data management procedures can be found, if not in the protocol	11,12; 19, 20
Statistical methods	20a	Statistical methods for analysing primary and secondary outcomes. Reference to where other details of the statistical analysis plan can be found, if not in the protocol	11,12
	20b	Methods for any additional analyses (eg, subgroup and adjusted analyses)	11,12
	20c	Definition of analysis population relating to protocol non-adherence (eg, as randomised analysis), and any statistical methods to handle missing data (eg, multiple imputation)	NA
**Methods: Monitoring**			
Data monitoring	21a	Composition of data monitoring committee (DMC); summary of its role and reporting structure; statement of whether it is independent from the sponsor and competing interests; and reference to where further details about its charter can be found, if not in the protocol. Alternatively, an explanation of why a DMC is not needed	NA
	21b	Description of any interim analyses and stopping guidelines, including who will have access to these interim results and make the final decision to terminate the trial	NA
Harms	22	Plans for collecting, assessing, reporting, and managing solicited and spontaneously reported adverse events and other unintended effects of trial interventions or trial conduct	13
Auditing	23	Frequency and procedures for auditing trial conduct, if any, and whether the process will be independent from investigators and the sponsor	NA
**Ethics and dissemination**			

Research ethics approval	24	Plans for seeking research ethics committee/institutional review board (REC/IRB) approval	13
Protocol amendments	25	Plans for communicating important protocol modifications (eg, changes to eligibility criteria, outcomes, analyses) to relevant parties (eg, investigators, REC/IRBs, trial participants, trial registries, journals, regulators)	13
Consent or assent	26a	Who will obtain informed consent or assent from potential trial participants or authorised surrogates, and how (see Item 32)	10, 11, 13
	26b	Additional consent provisions for collection and use of participant data and biological specimens in ancillary studies, if applicable	NA
Confidentiality	27	How personal information about potential and enrolled participants will be collected, shared, and maintained in order to protect confidentiality before, during, and after the trial	13, 19, 20
Declaration of interests	28	Financial and other competing interests for principal investigators for the overall trial and each study site	20
Access to data	29	Statement of who will have access to the final trial dataset, and disclosure of contractual agreements that limit such access for investigators	19, 20
Ancillary and post-trial care	30	Provisions, if any, for ancillary and post-trial care, and for compensation to those who suffer harm from trial participation	NA
Dissemination policy	31a	Plans for investigators and sponsor to communicate trial results to participants, healthcare professionals, the public, and other relevant groups (eg, via publication, reporting in results databases, or other data sharing arrangements), including any publication restrictions	13, 20
	31b	Authorship eligibility guidelines and any intended use of professional writers	NA
	31c	Plans, if any, for granting public access to the full protocol, participant-level dataset, and statistical code	20

Appendices

For peer review only - http://bmjopen.bmj.com/site/about/guidelines.xhtml

Informed consent materials	32	Model consent form and other related documentation given to participants and authorised surrogates	NA<br>We plan to post these on the study website
Biological specimens	33	Plans for collection, laboratory evaluation, and storage of biological specimens for genetic or molecular analysis in the current trial and for future use in ancillary studies, if applicable	NA

*It is strongly recommended that this checklist be read in conjunction with the SPIRIT 2013 Explanation & Elaboration for important clarification on the items. Amendments to the protocol should be tracked and dated. The SPIRIT checklist is copyrighted by the SPIRIT Group under the Creative Commons "Attribution-NonCommercial-NoDerivs 3.0 Unported" license.

For peer review only
