# Supplementary material for: Family-focused intervention to promote adolescent mental health and well-being in Moldova and North Macedonia (FLOURISH): feasibility study protocol
Source: BMJ Open. 2023 Dec 10;13(12):e080400. doi: 10.1136/bmjopen-2023-080400 (PMC11148709; doi:10.1136/bmjopen-2023-080400)
Supplement: Reviewer comments [file bmjopen-2023-080400.reviewer_comments.pdf]

PEER REVIEW HISTORY

BMJ Open publishes all reviews undertaken for accepted manuscripts. Reviewers are asked to complete a checklist review form (**http://bmjopen.bmj.com/site/about/resources/checklist.pdf**) and are provided with free text boxes to elaborate on their assessment. These free text comments are reproduced below.

ARTICLE DETAILS

**TITLE (PROVISIONAL)**	Family-focused intervention to promote adolescent mental health and well-being in Moldova and North Macedonia (FLOURISH): Feasibility study protocol
**AUTHORS**	Shenderovich, Yulia; Piolanti, Antonio; Babii, Viorel; Calovska-Hertzog, Nevena; Evans, Rhiannon; Heinrichs, Nina; Isakov, Anita Burgund; Lesco, Galina; Moore, Graham; Mueller, Janina; Raleva, Marija; Shimbov, Bojan; Simon, Judith; Waller, Franziska; Wienand, Dennis; Foran, Heather

This article was not externally reviewed at BMJ Open. Protocols that have been independently assessed prior to submission to BMJ Open are usually fast-tracked to publication on the grounds that further substantial changes will not be possible. This independent assessment will usually be external, independent review for both a substantial grant award from a non-commercial or government funder and ethics approval.
